# Comparative RNAi Screens in *C. elegans* and *C. briggsae* Reveal the Impact of Developmental System Drift on Gene Function

**DOI:** 10.1371/journal.pgen.1004077

**Published:** 2014-02-06

**Authors:** Adrian J. Verster, Arun K. Ramani, Sheldon J. McKay, Andrew G. Fraser

**Affiliations:** 1 The Donnelly Centre, University of Toronto, Toronto, Ontario, Canada; 2 Department of Molecular Genetics, University of Toronto, Toronto, Ontario, Canada; 3 Cold Spring Harbor Laboratory, Cold Spring Harbor, New York, United States of America; University of California Davis, United States of America

## Abstract

Although two related species may have extremely similar phenotypes, the genetic networks underpinning this conserved biology may have diverged substantially since they last shared a common ancestor. This is termed Developmental System Drift (DSD) and reflects the plasticity of genetic networks. One consequence of DSD is that some orthologous genes will have evolved different *in vivo* functions in two such phenotypically similar, related species and will therefore have different loss of function phenotypes. Here we report an RNAi screen in *C. elegans* and *C. briggsae* to identify such cases. We screened 1333 genes in both species and identified 91 orthologues that have different RNAi phenotypes. Intriguingly, we find that recently evolved genes of unknown function have the fastest evolving *in vivo* functions and, in several cases, we identify the molecular events driving these changes. We thus find that DSD has a major impact on the evolution of gene function and we anticipate that the *C. briggsae* RNAi library reported here will drive future studies on comparative functional genomics screens in these nematodes.

## Author Summary

Although two related species may appear similar, the genetic pathways that underpin this shared biology may have drifted and changed. This phenomenon is known as Developmental System Drift (DSD). One consequence of DSD is that equivalent genes may play different roles in phenotypically similar, related species but there have been no systematic studies to examine this. How often do genes have different functions in similar species? Are certain genes more likely to change functions? Finally, what are the molecular changes that drive this? Here, we compare the effects of reducing the levels of over 1300 different genes in two species of nematode worm. These worms are very similar— they live in the same ecological niche, and have near-identical development and behavior. We find that over 25% of conserved genes have different functions in these two species, showing that DSD has a major impact on how gene function evolves. Intriguingly, we find that genes that have arisen recently are most likely to change functions and that this is often driven by changes in their expression. This is the first systematic comparison of loss of function phenotypes in related species and sheds light on how genetic pathways rewire during DSD.

## Introduction

As genomes evolve, new genes are born and older genes may adopt novel functions, fuse, or disappear altogether. What are the phenotypic consequences of this continual molecular change?

One striking consequence of the evolution of genomes is adaptation: novel genetic variants can underpin the evolution of novel organism-level phenotypes such as new anatomical structures or behaviors and, if these result in improved fitness, these can become fixed in the population through selection. At the molecular level, such novel organism-level phenotypes can arise through the evolution of entirely novel biochemical activities such as novel genes, new protein domains, or new classes of functional RNAs: for instance, metazoan genomes encode classes of proteins that are absent from single-celled eukaryotes and that participate in metazoan-specific processes (e.g. netrins in axon guidance, immunoglobulins and MHC complex subunits in the immune system). New organism-level phenotypes can also result from the rewiring of already existing activities such as the shuffling of existing domains into novel combinations (e.g. the rapidly evolving architectures of chromatin regulators [1]) or through changes in the regulation of expression of otherwise conserved genes — for example, evolution of *lin-48* expression affects salt tolerance in *C. elegans* [2], evolution of the *yellow* gene alters wing spots in different *Drosophila* species [3], and evolution at the *Pitx1* locus causes adaptive loss of pelvic spines in sticklebacks [4]. Adaptation is dependent on changes in the molecular phenotype of the organism — the functional activities encoded by the genome and the way they are regulated — which result in selectable changes in the phenotype of the organism.

At the other end of the spectrum from adaptation is neutral drift. Many genomic changes have no impact on the phenotype of the organism since they do not have any impact on the molecular phenotype, that is, on the functions encoded in the genome and their precise regulation. Such changes are therefore under no selection — while they may disappear or become fixed in a species, neither outcome is a consequence of their effect on phenotype.

**Figure 1:**
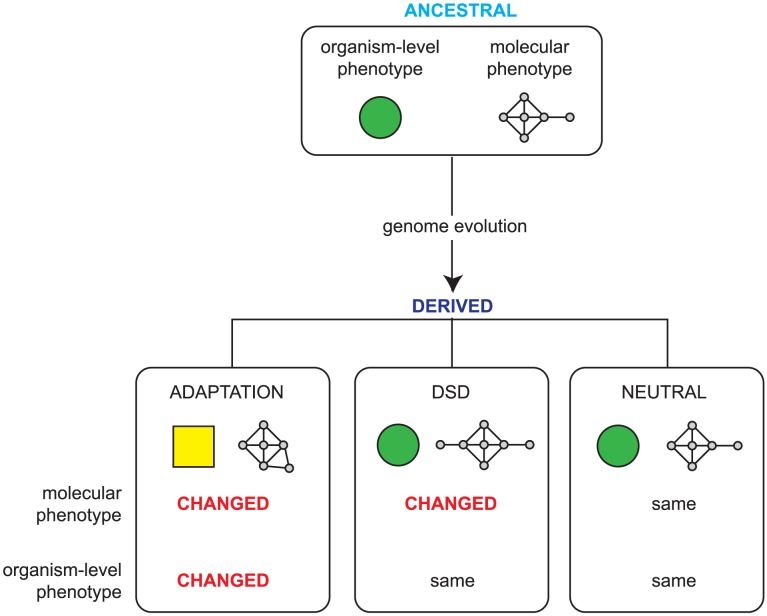
Possible outcomes of genome evolution. As a genome evolves, the accumulated mutations can be neutral, having no impact on the molecular phenotype (that is, the functions encoded in the genome and the ways that these are regulated), or they can lead to adaptation via changes in heritable phenotype due to changes in the molecular phenotype. Developmental System Drift (DSD) describes a third possibility: while the overall phenotype of the organism remains identical, the underlying genetic networks underpinning this phenotype have changed. A key outcome of this is that some orthologous genes play different *in vivo* roles in phenotypically identical, related species.

All changes in organism-level phenotype (such as those that result in adaptation) are thus underpinned by changes in molecular phenotype and, conversely, genomic changes that do not affect molecular phenotype cannot alter organism level phenotype and are therefore neutral. However, there is a third outcome, a phenomenon known as ‘Developmental System Drift’ (DSD) [5]. In DSD, shown schematically in Figure 1, two related species share an identical organism-level function that was also present in their last shared ancestor; however, since the species diverged, the genetic networks that underpin this function have drifted. Unlike in classical drift, molecular change in DSD is under strong stabilizing selection to preserve the phenotype of the organism. In DSD then, the molecular phenotype has changed, while the organism-level phenotype has remained unaltered; this is a reflection of the plasticity of genetic networks.

One effect of the changes in molecular phenotype that accompany DSD is that some orthologues evolve different roles in related organisms — these will therefore have different loss of function phenotypes. If we knew the entire set of orthologous genes that have different loss of function phenotypes in two related species that have very similar phenotypes, this would provide a global view of how gene function can drift while maintaining the same organism level phenotype — this is our goal here. Specifically, by examining how DSD affects gene function in a systematic manner, we would like to examine whether the *in vivo* function of certain classes of genes evolves faster than others and begin to explore the molecular changes the underpin the types of changes in gene function that nonetheless preserve the same overall organism-level phenotype.

*C. elegans* and *C. briggsae* are both free-living hermaphroditic nematodes that share the same ecological niche [6]. Their anatomical structures are strikingly similar and, up to the 350-cell stage of embryogenesis, the lineages and timings of cell division are nearly identical [7]. However, their genomes have diverged significantly in the ∼20 Mya since they last shared a common ancestor [8]: only ∼60% of their genes have 1∶1 orthologues, with many species-specific expansions, losses, and chromosomal rearrangements [9]. There is already good evidence that while *C. elegans* and *C. briggsae* have very similar biology, the genetic networks that control this are not the same, since while they can fertilize each other, the resulting interspecific hybrids die as embryos [10]. More specifically, a small number of genes is also known to play very different roles in otherwise identical processes — for example, while early embryogenesis is identical in both species, knocking down the Wnt-pathway effector *pop-1* by RNA-mediated interference (RNAi) causes opposite cell fate transformations in the two nematodes [11], [12]. Thus, while many of the organism-level phenotypes functions are highly conserved between these two worms, the genetic networks underpinning these functions may have diverged considerably. A systematic comparison of loss of function phenotypes between orthologous genes in these two related nematodes might thus shed light on how DSD affects gene function.

RNAi-based screens have been used extensively in *C. elegans* to identify the *in vivo* (i.e. organism-level) functions of each gene [13]–[16]. However, no analogous screens have been carried out in *C. briggsae*. In this paper, we describe the construction of a *C. briggsae* RNAi library of 1333 dsRNA-expressing bacterial strains analogous to the well-characterized *C. elegans* RNAi library [13], [14] — feeding any single bacterial strain to *C. briggsae* targets a single *C. briggsae* gene. The genes targeted in the library are the great majority of the *C. briggsae* 1∶1 orthologues of *C. elegans* genes that have a well-characterized RNAi phenotype (see Methods). Comparing the RNAi phenotypes of the *C. briggsae* gene with the RNAi phenotypes of its *C. elegans* orthologue thus allows identification of orthologues that have different loss of function phenotypes in these two worms indicating that they play different roles in the development and function of these anatomically highly similar animals.

In this paper we report the construction of a *C. briggsae* RNAi library and a screen to identify orthologues that have different RNAi phenotypes in *C. elegans* and *C. briggsae*. Our data indicate that while these two species have very similar morphology and behavior, many orthologous genes have different *in vivo* functions suggesting that DSD has a major impact on the evolution of gene function.

## Results

### Construction and screening of the *C. briggsae* RNAi library

RNAi is an extremely powerful tool for examining gene function in *C. elegans* [17]. RNAi allows the knock-down of any gene *in vivo* and thus can be used to rapidly identify the role any gene plays in the development and function of the worm, that is, its organism-level function. In *C. elegans*, RNAi can be induced by feeding worms with bacteria expressing dsRNA complementary to a gene of interest (so-called ‘RNAi by feeding’, [18], [19]) and a library of dsRNA-expressing bacteria has been constructed that allows the researcher to individually target over 80% of all predicted *C. elegans* genes [13], [14]. We wished to construct an analogous library for *C. briggsae* and use it to compare RNAi phenotypes of orthologous genes between species.

Constructing and screening a genome-scale RNAi library for *C. briggsae* is a huge undertaking. Since our principal goal was to identify genes that have different RNAi phenotypes in *C. elegans* and *C. briggsae*, the great majority of genes will be uninformative since they will have no readily detectable RNAi phenotype in either worm (∼85% of genes have no readily detectable phenotype in *C. elegans* [14], and this is likely to be broadly similar in *C. briggsae*). We thus decided to construct a library targeting only the set of 1437 *C. briggsae* genes that had direct 1∶1 orthologues with the 1640 genes which were previously shown to have a robust, readily detectable RNAi phenotype in *C. elegans* [14] (see Methods, Figure S1A). Although this excludes a small number of genes that have no apparent phenotype in *C. elegans* but that have a phenotype in *C. briggsae*, this set will nonetheless cover the great majority of genes that have phenotypes in *C. briggsae*. We made the library according to the same design principles as the *C. elegans* RNAi library [13], [14], and as far as possible targeted an orthologous region of the *C. briggsae* gene as was targeted by the *C. elegans* RNAi fragment (Figure S1B). In total, we were able to construct targeting strains for 93% (1333) of the 1437 targeted genes in *C. briggsae* (Methods, Figure S1A).

The central goal of this project is to compare the loss of function phenotypes of orthologous genes in *C. elegans* and *C. briggsae* — accurate identification of orthologues is thus critical. We initially used InParanoid 6.1 [20] to identify putative 1∶1 orthologues — these candidates are similar to candidates that would identified using reciprocal BLAST, and this is a reasonable place to start. To increase our confidence that the identified putative orthologues are indeed likely to be true orthologues, we carried out three sets of additional tests. First, we determined whether there are additional closely related genes in either genome, in which case orthology can be harder to assign, or whether the putative orthologues appear to be the sole related gene in either genome, in which case orthology is fairly unambiguous. For example, *K04G7.1* and *CBG16609* are reciprocal best hits and have a BLAST E-value of 0 in either direction; in *C. briggsae*, the next closest BLAST hit is *CBG20138*, with a E-value of 8*10^−4^, and in the other direction, the next closest *C. elegans* hit is *C01H6.2* with an E-value of 4.3. When the difference in E-value is greater than 20 on a log10 scale we called these unambiguous and 72% of our orthologue pairs fall into this class. Second, we checked whether the orthologue pairs identified via InParanoid, a graph-based method, were also identified using a tree-based method, which is a very different and complementary approach [21]. In this case we used TreeFam [22] and we found that 90% of our putative orthologues are identified as orthologues in TreeFam. Finally, we used synteny to resolve harder assignments. Alignments of the *C. elegans* and *C. briggsae* genome indicate that considerable proportions of these genomes are syntenic [9], that is, many segments can be identified in which gene order has been preserved in both species since the last common ancestor. Synteny can be used to aid in identification of likely true orthologues in complex cases (e.g. large families of closely related genes, or cases where orthologues have diverged greatly). We were able to find evidence of upstream or downstream synteny in 87% of cases. Together these results suggest that our orthologue identification is correct in the great majority of cases — 72% are unambiguous, and a further 27% of the putative orthologues can be confirmed either through TreeFam or synteny — thus 99% of our orthologues can be confirmed by other complementary approaches. These data are all summarized in Table S1.

**Figure 2:**
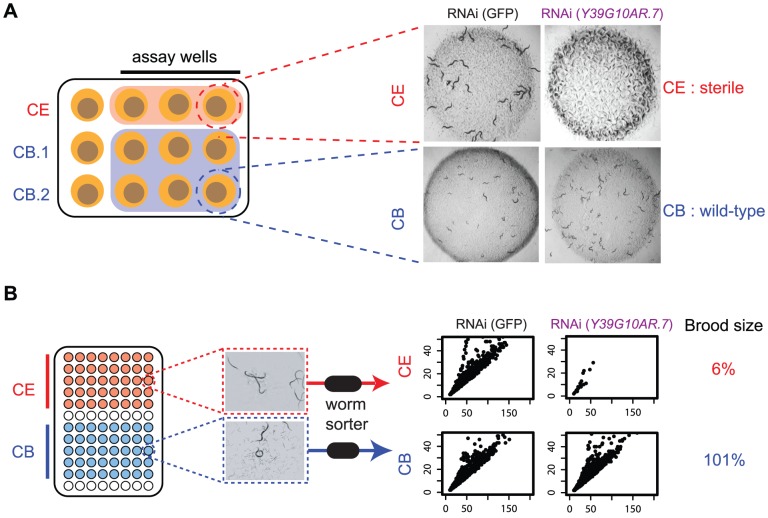
Outline of screening procedures. **A. Manual comparison of RNAi phenotypes in both species.** RNAi phenotypes are screened by eye on 12 well plates. For each gene being examined, three replicates of the *C. elegans* RNAi and six replicates of the *C. briggsae* RNAi clone are screened; in each replicate, phenotypes are examined in the progeny of a single adult worm that has been exposed to dsRNA-expressing bacteria for 72 hrs. Each plate was scored by two people at two time-points (24 hrs and 48 hrs after removal of adult), as described in Kamath *et al.* In the example shown, knockdown of *Y39G10AR.7* produces a sterile phenotype in *C. elegans* but not in *C. briggsae*. **B.** **Automated analysis of RNAi phenotypes.** RNAi was carried out in liquid cultures in 96-well plates as described in Methods. Each well was sampled with a commercially available worm sorter and we quantified the number of animals in each well, as well as the size (Time of Flight, TOF) and optical density (Extinction, Ext) of each animal. The scatter plots show Time of Flight (TOF, x-axis) and Extinction (EXT, y-axis) for the population in a representative well of either *C. elegans* or *C. briggsae* following RNAi targeting *Y39G10AR.7*. In addition, the corresponding brood size defect is shown (see Methods for calculation).

To screen the *C. briggsae* library, we followed an identical screening protocol to that used in the first genome-scale screens in *C. elegans* [13], [14] and assessed the same developmental and morphological phenotypes (see Methods for a complete list). However, while wild-type *C. briggsae* is capable of RNAi when the dsRNA is delivered by injection, RNAi by feeding is ineffective at least in part because of the inability of the *C. briggsae* SID-2 to actively uptake dsRNA [23]. This defect can be rescued by transgenic expression of *C. elegans sid-2* [24], however, and thus all our screening was not in wild-type *C. briggsae* but in a transgenic line expressing *C. elegans sid-2*. We note that this could produce some false positive results due to genetic interactions in the background we are using, such as synthetic lethality with the expression of SID-2, but this is likely to be only a minority of cases. To identify genes with different phenotypes in *C. elegans* and *C. briggsae*, we not only compared the phenotypes in *C. briggsae* to previously published data for *C. elegans* [14] but we also screened *C. elegans* side by side with *C. briggsae* as shown schematically in Figure 2A. The RNAi phenotypes of each pair of orthologues were compared in the two species at two time points by two independent observers; three *C. elegans* replicates and six *C. briggsae* replicates were examined in any single experiment. Any differences were repeated in an independent experiment, and genes where we detected a different phenotype in at least 3 out of 4 observations between the 2 observers and 2 experiments were considered as potential hits. Based on these criteria, we examined the loss of function phenotypes of 1333 orthologous genes by RNAi in *C. elegans* and *C. briggsae* and identified 679 orthologues that have different phenotypes in the two species (Figure S2A).

There are two major sources of false positives in this initial screen, which we try to deal with using secondary filters and rescreening. The first source of false positives in our primary screen is that RNAi is more efficient in the transgenic SID-2-expressing line of *C. briggsae* than in *C. elegans* — we generally get a stronger RNAi knockdown of *C.briggsae* genes as measured by qPCR (see Figure S3). Many genes thus have stronger RNAi phenotypes in *C. briggsae* (eg. *ytk-6* has a growth defect in *C. elegans* but is completely sterile in *C. briggsae*) but this does not reflect any true difference in *in vivo* function. To partly test this idea, we tested a 111 gene subset of the 508 genes that have a stronger phenotype in *C. briggsae* in the *lin-35(n745) C. elegans* strain, which has increased RNAi efficiency compared with wild-type *C. elegans*. We find that a substantial proportion of these genes (36%; 40/111) also have stronger phenotypes in *lin-35(n745)* worms than in wild-type *C. elegans* which provides some support for the view that the stronger phenotypes seen for many genes in *C. briggsae* may be due to an increased level of knockdown in *C. briggsae* than *C. elegans*. Crucially, however, this increased RNAi efficiency in *C. briggsae* means that in the cases where the RNAi phenotype is *weaker* in *C. briggsae*, this is not due to a weaker knockdown in *C. briggsae* (as shown by qPCR for a number of cases in Figure S3), rather that it reflects a genuine difference in the *in vivo* function. We thus focus the rest of the paper on studying genes whose phenotypes are weaker in *C. briggsae* than *C. elegans* and excluded all genes that had stronger RNAi phenotypes in *C. briggsae* from any downstream analysis.

The second source of false positives is that some of the *C. briggsae* RNAi library clones do not produce adequate knockdowns in *C. briggsae* — these genes will thus appear to have weaker phenotypes in *C. briggsae* than in *C. elegans*. To address this, we made independent RNAi clones targeting a different region of the gene to that used in the primary screen (where possible) and screened these. We re-examined the RNAi phenotypes of all 204 genes that had weaker phenotypes in *C. briggsae* in this way and found that 91 genes still showed reproducibly weaker phenotypes in *C. briggsae* with the independent clones (final breakdown of hits is shown in Figure S2B, genes are shown in Table S2). We note that while rescreening with independent targeting clones is fairly rigorous, it is still possible that both independent clones failed to generate good knockdown in *C. briggsae*. To assess how often this may happen, we used qPCR to examine levels of knockdown in *C. elegans* and *C. briggsae* for genes that have weaker phenotypes in *C. briggsae* — of the 8 genes examined, 7 showed similar or stronger knockdown in the SID-2 expressing transgenic *C. briggsae* than in *C. elegans* (and thus are true positives) and only a single example had weaker knockdown in *C. briggsae*. This last example, *tsr-1*, is a false positive in our dataset. We thus estimate that around 80–90% of our hits are true positives, but acknowledge that a few rare examples are false positives due to poor knockdown in *C. briggsae*.

As a final confirmation of the differences in RNAi phenotype seen using the manual phenotyping described above, we retested 50 of the hits from our manual screen and a random subset of 324 additional genes using a fully automated phenotyping method (shown schematically in Figure 2B). This is highly complementary to manual screening. The manual screening described above has many advantages — multiple time-points are examined, many phenotypes are scored at once and, for the purposes of this screen, it allowed us to assess RNAi phenotypes in *C. briggsae* using the exact same methodology used for the initial screens in *C. elegans*. One disadvantage, however, is that it is not fully quantitative and this affects sensitivity in two ways. Firstly, there is a limit to what the eye can detect at high throughput: while differentiating between a sterile worm and one with a normal brood size is trivial, it is hard to tell the difference between a worm that has 50% of normal brood size and one that has 35% normal brood size. Secondly, different worm strains and especially different worm species do not grow identically. The *C. briggsae sid-2*-expressing transgenic line that we use for all our experiments grows slightly more slowly than N2, and this inherent difference in growth rate can make identification of subtle differences in phenotype more difficult. For these reasons, we also carried out a fully automated quantitative screen using a commercially available worm sorter (Union Biometrica) which addresses both the issues of sensitivity and normalization for different growth of the two species.

In outline, RNAi experiments are set up in liquid culture in 96-well format. At the start of the experiment, each well contains a saturated culture of dsRNA-expressing bacteria and 10 L1 worms; phenotypes are examined after 96 hours by which time, in a normally growing culture, the initial L1 animals have grown to fertile adults, laid the next generation, and these will have hatched. Using the worm sorter, we quantify the number of worms in each well, as well as the sizes and optical densities of each worm in each well. These data allow us to precisely measure brood size as well as identify differences in growth rate, body size, and embryonic lethality (see Methods for more details in analysis). Crucially, by comparing the phenotypes seen after targeting a specific gene with phenotypes of worms growing in bacteria expressing a control non-targeting dsRNA, all phenotypes are normalized for any inherent differences in worm growth between the two species. Using this pipeline, we confirmed statistically significant differences in phenotype for 26 of the 50 tested manual phenotyping hits; 21 showed brood size differences and a further 5 showed differences in growth rate or embryonic lethality (see Methods for data processing details). We failed to see differences in phenotype for 24 — the majority of these show subtle phenotypic differences (e.g. cuticle defects, or movement defects) that are not readily detectable in the sorter and we believe this explains the difference in the two assays. Finally, we note that we see an additional 57 genes having significantly different effects on brood size in these two species using the automated pipeline, suggesting that the true number of genes with different phenotypes in these two species is significantly greater than was detected by manual phenotyping which has few false positives but a substantial false negative rate. All data from the automated pipeline are in Table S3.

In summary, we constructed an RNAi library of targeting 1333 *C. briggsae* genes. We used this library to compare the RNAi phenotypes of orthologues in *C. elegans* and *C. briggsae* using manual phenotyping and identified 91 genes that have different RNAi phenotypes in these two species that is likely to be due to a genuine difference in their *in vivo* function. The majority of these differences could be confirmed by a quantitative phenotyping method designed specifically to measure differences in brood size, lethality, and growth rate. This list of genes undoubtedly has some false positives due to inadequate RNAi knockdown in *C. briggsae* (e.g. the example of *tsr-1* above, or *pal-1* which has a detectable embryonic lethal phenotype in *C. briggsae* when using RNAi by soaking [25], but has no phenotype in our screen) — however, our qPCR analysis suggests that only ∼15% of our reported hits are such false positives and thus that the great majority of our hits are true positives. The rest of this paper is concerned with examining this set of genes to explore the molecular changes that underlie this difference.

### Genes with different phenotypes are enriched for transcription factors and recently evolved novel genes

**Figure 3:**
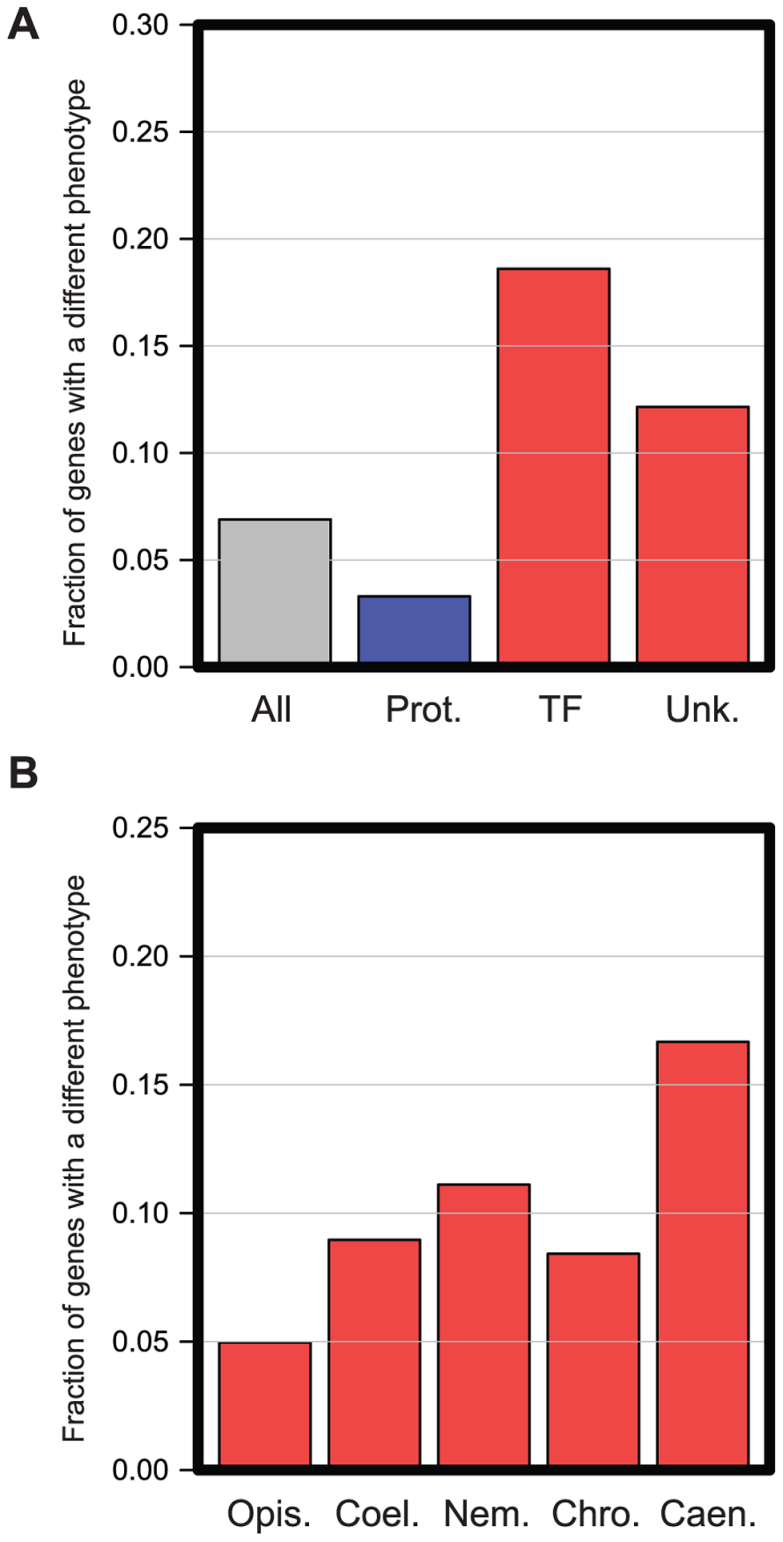
Functional enrichment in genes with different *in vivo* functions in *C. elegans* and *C. briggsae*. **A.** All 1333 genes analyzed were manually placed into the functional categories described in Kamath *et al.* [14]. The graph shows the proportion of genes that have different RNAi phenotypes in several different functional classes: all genes analyzed (‘All’), genes annotated to have roles in Protein Synthesis (Prot. Synth.), Transcription Factors (‘TF’), or genes of Unknown function (‘Unk’). Classes with significantly fewer genes with different RNAi phenotypes are shown in blue; those with statistically increased numbers are shown in red. Enrichments are significant with an FDR of 0.05 (Hypergeometric test). **B.** **RNAi phenotypes differ most for more recently evolved genes.** All 1333 genes analyzed were placed into five classes based on their evolutionary age as described in Methods. The most ancient genes could be dated back to the emergence of the *Opisthokta* lineage (‘Opis.’), then becoming progressively younger, we could date sets of genes back to the emergence of the *Coelomata* (‘Coel.’), *Nematoda* (Nem.), *Chromadorea* (‘Chro.’), and finally some genes had arisen so recently that they were only detectable in *Caenorhabditis* species (‘Caen.’). In each case, the graph shows the proportion of genes in each evolutionary class that had a different RNAi phenotype.

We identified 91 genes that have a different RNAi phenotype in *C. elegans* and *C. briggsae* — we refer to these from here on as the ‘Different Function’ genes. To begin to understand why these ‘Different Function’ genes have such differing *in vivo* roles, we initially assessed whether this set of genes was enriched for any specific molecular functions. We annotated genes into the functional categories previously used by Kamath *et al.* [14] and find that transcription factors and genes of unknown function are enriched among the ‘Different Function’ genes, while genes involved in protein synthesis are under-enriched (Figure 3A, *p*<0.01, Hypergeometric test). This indicates firstly that the basic machinery of the eukaryotic cell has changed very little in organismal function over time and, secondly, suggests that transcription factors appear to have more rapidly evolving organismal roles than other classes of gene. These two findings are unsurprising. The individual genes that encode for components of the basic eukaryotic cell machineries (e.g. DNA replication, transcription, translation etc.) are essential in organisms as divergent as worms and yeasts [14], [26], so finding great similarity in these genes between two related species is expected. Likewise, transcription networks are well-known to be extremely plastic across evolution [27] and thus finding an enrichment of transcription factors in the set of genes with different *in vivo* functions in *C. elegans* and *C. briggsae* is not unexpected. However, the finding that genes with different phenotypes are enriched for genes of unknown function is intriguing since almost all of these ‘unknown function’ genes have evolved *de novo* from previously non-coding DNA relatively recently and are often nematode-specific (see analysis below). This suggested that more recently evolved genes may have most rapidly changing *in vivo* roles and we examined this further.

To investigate more closely whether there was any correlation between the evolutionary age of a gene (i.e. when any such gene arose *de novo* from non-coding sequences) and the likelihood that it had a different *in vivo* function between *C. elegans* and *C. briggsae*, we carried out a phylogenetic analysis for each gene screened and date the emergence of these genes to their last common ancestor in a similar method to the ‘phylostratum’ approach [28] (see Methods). We find that the more recently a gene has arisen, the more likely it is to have a different phenotype between *C. elegans* and *C. briggsae*. Ancient genes (those that we were able to date to the emergence of the *Opisthokont* lineage) are the least likely to show a difference in phenotype (<5%, *p*<0.01 Hypergeometric test, Figure 3B) while extremely recently evolved genes (those which date to the emergence of the *Caenorhabditis* genus) are the most likely (>15%, *p*<0.01 Hypergeometric test, Figure 3B), suggesting that phylogenetically novel genes have a high rate of evolution of their *in vivo* functional roles.

These bulk analyses thus reveal that just as changes in transcriptional networks and the ‘invention’ of entirely novel classes of gene are major forces driving the evolution of novel organismal functions in adaptive evolution (for example, [29]), these classes of gene are those that have fastest evolving *in vivo* functions during DSD.

### Changes in gene function during DSD are often the result of promoter evolution

**Figure 4:**
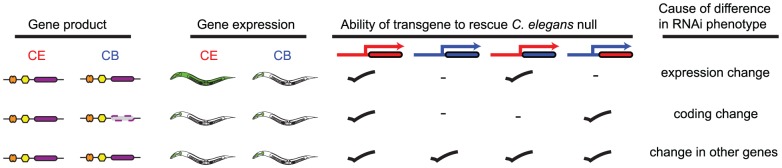
Schematic illustrating transgenic rescue approach. The transgenic rescue approach illustrated here shows the possible molecular events driving changes in gene function and how transgenic rescue with various hybrid rescue constructs would be interpreted to differentiate between these. In each case regions of constructs in red are derived from *C. elegans* while regions of construct in blue are derived from *C. briggsae*. Coding regions are shown as coloured boxes.

We found that the 91 genes that have significantly different *in vivo* functions in *C. elegans* and *C. briggsae* are enriched for both transcription factors and for recently evolved genes of known function. However, this does not tell us *why* they have different *in vivo* functions (and thus different RNAi phenotypes). There are three possible reasons that orthologous genes could have a different RNAi phenotype in *C. elegans* and *C. briggsae*, shown schematically in Figure 4: they might encode the same molecular function but be expressed in different tissues, the coding sequences might have diverged such that they have different molecular functions, or, while the orthologues are functionally identical both in terms of expression and encoded functions, changes in some other genes may have altered the level at which these orthologues are required in these two worms. We examined each possibility in turn.

We initially focused on testing whether genes with different RNAi phenotype in *C. elegans* and *C. briggsae* might have different expression patterns in these two species. This could be due to many different levels of gene regulation from transcriptional to post-transcriptional and translational control — for the purposes of these analyses, we focused on transcriptional control of gene expression since this is a major step of regulation of gene expression. In outline, we used PCR stitching [30] to generate pairs of constructs in which either the promoter of the *C. elegans* gene drives GFP expression or the syntenic region of the orthologous *C. briggsae* promoter drives expression of mWormCherry. In this way, we could make *C. elegans* worms transgenic for both constructs and rapidly identify cells that were either exclusively GFP or mWormCherry positive, indicating that the *C. elegans* and *C. briggsae* orthologues might be expressed in different cell types. In any cases where we found differences in *C. elegans*, we repeated the experiment in *C. briggsae* to test whether any differences in tissue expression were due to evolved changes in the promoters or any changes in *trans*-acting factors.

**Figure 5:**
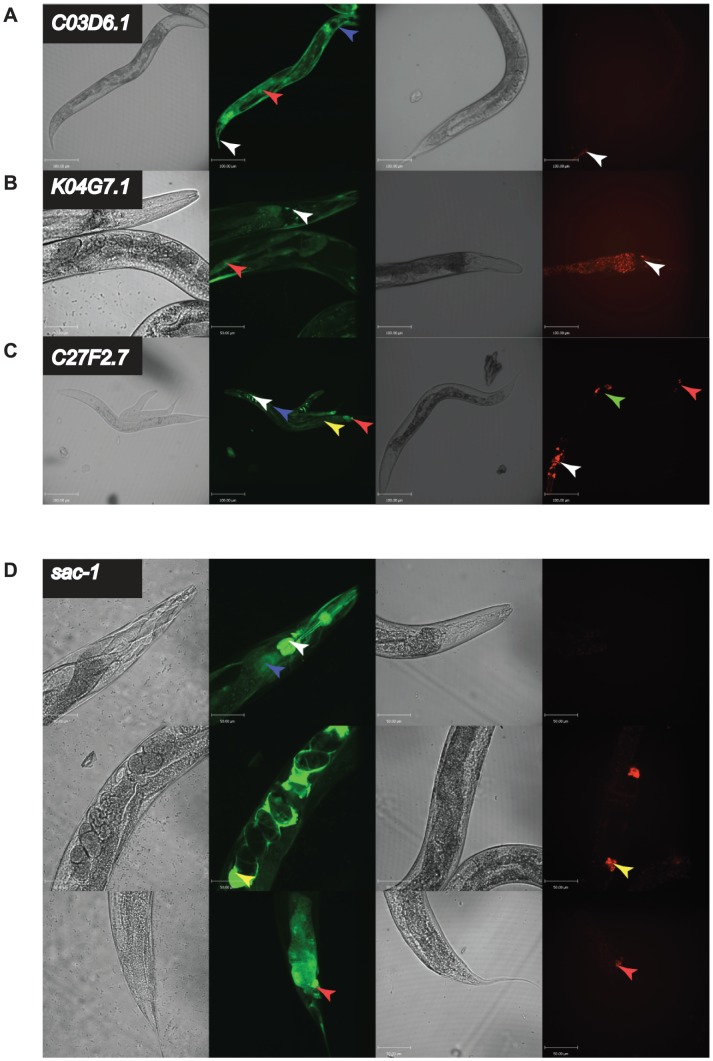
*in vivo* expression of a subset of genes with different RNAi phenotypes. *C03D6.1, K04G7.1*, *C27F2.7*, and *sac-1* had strongly different RNAi phenotypes in *C. elegans* and *C. briggsae*. We generated transgenic *C. elegans* strains (N2) expressing GFP under control of the promoter of the *C. elegans* orthologue or *C. briggsae* strains (AF16) expressing mWormCherry under control of the orthologous *C. briggsae* promoter for each gene. In each case, four panels are shown: DIC image of N2 worms transgenic for the *C. elegans* promoter driving GFP, fluorescence image of N2 worms transgenic for the *C. elegans* promoter driving GFP, DIC image of AF16 worms transgenic for the *C. briggsae* promoter driving mWormCherry expression, fluorescence image of AF16 worms transgenic for the *C. briggsae* promoter driving mWormCherry expression. Images are confocal projections at 200× magnification, and scale bars represent 100 µm, except for *C. elegans K04G7.1* which is at 400× magnification with a scale bar representing 50 µm. Images are representative of 3 independent lines. **A.** **Expression difference for** ***C03D6.1*** **.** Arrow heads indicate tail cells (white), intestine (red) and hypodermis (blue). **B.** **Expression difference for** *K04G7.1* **.** Arrow heads indicate head neurons (white) and body wall muscle (red). **C.** **Expression difference for** *C27F2.7* **.** Arrow heads indicate head neurons (white), hypodermis (blue), intestine (yellow), vulva (green) and tail neurons (red). **D.** **Expression difference for** *sac-1* **.** Shown are 3 confocal projections along the body of the animals 400× magnification. Scale bars represent 50 µm. Arrowheads indicate the intestine (blue), pharynx and pharyngeal neurons (white), spermatheca (yellow), and tail cells (red).

Since it would have been an impractical amount of work to do this analysis for all 91 orthologue pairs, we focused our effort on examining the expression patterns of the ‘Different Function’ genes of unknown function that are uniquely found in nematode genomes since this class of gene was enriched in our dataset. We analyzed expression patterns for 12 such worm-specific ‘Different Function’ genes; in addition, to sample other gene classes, we examined expression patterns of 10 random ‘Different Function’ genes in our dataset. We identified 3 worm-specific orthologues, *C03D6.1*, *K04G7.1*, and *C27F2.7*, that had clearly visible differences in expression pattern between *C. elegans* and *C. briggsae* (Figure 5A–C); in addition, one gene in our random set, *sac-1*, also had a different expression pattern in the two species (Figure 5D). In all four cases this was due to differences in the promoter and not to differences in *trans*-acting factors since the expression patterns seen in *C. elegans* could be faithfully recapitulated in *C. briggsae* (all data shown in Figure 5 are expression patterns in transgenic *C. briggsae*). Crucially, in all four cases, the difference in expression pattern is likely to explain the difference in phenotype since the tissue expression in *C. briggsae*, where the phenotype is weaker, is a restricted subset of the tissue expression in *C. elegans*. For example, *C03D6.1* has a strong growth defective RNAi phenotype in *C. elegans* and is expressed in the gut, the hypodermis, and a small number of tail cells; in *C. briggsae*, where its expression is restricted to only a handful of cells in the tail, it has no obvious phenotype at all.

These data strongly suggest that the reason for the differences in RNAi phenotypes between *C. elegans* and *C. briggsae* for the four genes examined here is that they are expressed in a very different set of tissues in these two animals, leading to a differential requirement for these genes for organismal viability. To test this prediction directly, we took a cross species rescue strategy. In outline, we examined the ability of a set of transgenes (shown schematically in Figure 4) to rescue the phenotype of a null *C. elegans* mutant and designed these to be able to test which parts of the *C. elegans* and *C. briggsae* genes are functionally interchangeable — the promoter, the coding region, neither, or both. Of the four orthologues that we could have tested, there was only a suitable null mutant for one of these, *sac-1*, and we focused our attention on this gene.

**Figure 6:**
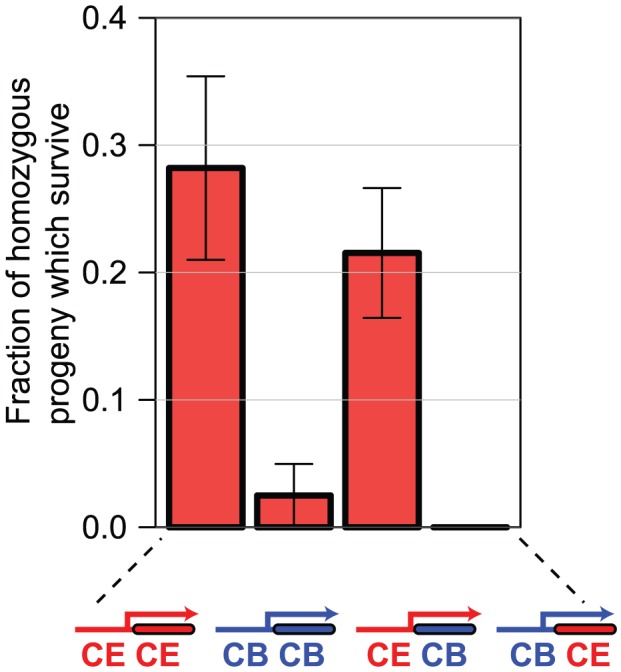
Differences in *sac-1* RNAi phenotype are due to differences in *sac-1* promoter function. We generated *C. elegans* lines transgenic either for the *C. elegans sac-1* ORF under control of the *C. elegans sac-1* promoter, the *C. briggsae sac-1* ORF under control of the *C. briggsae sac-1* promoter, or for the two hybrid constructs shown. In each case, we examined the ability of the transgenic array to rescue the developmental arrest phenotype of *sac-1(ok1602)* homozygous animals — the graph shows the percentage of animals that reached the adult stage that are homozygous for the *sac-1(ok1602)* allele, indicating rescue. Either the *C. elegans sac-1* ORF or the *C. briggsae sac-1* ORF under control of the *C. elegans sac-1* promoter could partially rescue; no rescue was seen for the remaining constructs, indicating that the *sac-1* promoter has diverged in the two species, while the *sac-1* coding regions appear to be functionally interchangeable.

We find that transgenic expression of the *C. elegans sac-1* ORF under control of the *C. elegans sac-1* promoter gives robust rescue of the growth arrest phenotype of *C. elegans* homozygous for the null allele *sac-1(ok1602)*, but that the *C. briggsae sac-1* ORF under control of the syntenic region of the *C. briggsae sac-1* promoter (see Figure S4) does not, indicating that these genes have indeed functionally diverged. When we use hybrid rescue constructs, we find that while the coding sequences are apparently functionally interchangeable, the promoters are not: only the *C. elegans* promoter drives expression in the correct tissues to rescue the *sac-1(ok1602)* phenotype (Figure 6). These data show that at least in the case of *sac-1* the difference in RNAi phenotype in *C. elegans* and *C. briggsae* is entirely due to promoter evolution.

### Orthologue pairs encoding more divergent protein sequences are more likely to have different RNAi phenotypes

We examined the expression patterns of 22 pairs of orthologues that have different RNAi phenotypes in *C. elegans* and *C. briggsae* and found that 4 of these have obviously different expression patterns, suggesting that promoter evolution underlies the differences in *in vivo* function that we observe for these genes. However, as shown in Figure 4 differences in *in vivo* function might also be due to evolution of coding sequences— if the *C. elegans* and *C. briggsae* orthologues encode different enzymatic activities, for example, this could result in different *in vivo* functions. Using a similar hybrid transgene rescue strategy to that for *sac-1* above, we tested whether coding sequences of *bli-4*, *bli-5*, *vha-5*, *flr-1*, *sma-3*, and *sem-5* were functionally interchangeable, or whether there was evidence that they had evolved functional differences. We selected these 6 genes since for each gene there was a null allele available in *C. elegans* that had a readily detectable phenotype; for most genes, there was no null allele available at the time, and thus we could not carry out similar tests for most of our dataset.

We found no clear examples where the difference in RNAi phenotype of orthologues in *C. elegans* and *C. briggsae* could be conclusively shown to be due to evolution of coding sequences. However, we only tested a very small number of cases and, in many of these cases, we failed to get strong enough rescue of the null phenotype by transgenic expression of the *C. elegans* coding sequence under the *C. elegans* promoter to allow us to distinguish between the ability of different hybrid transgenes to give different levels of rescue. These are therefore inconclusive experiments and, as more null alleles are being generated, it will be interesting to revisit this. We note however that bulk analyses of the protein sequences encoded by *C. elegans* and *C. briggsae* orthologues indicates that divergence of protein sequence between orthologues does appear to correlate with the likelihood that orthologues have different RNAi phenotypes. We compared the proteins encoded by orthologous ‘Different Function’ genes in *C. elegans* and *C. briggsae* and find that the ‘Different Function’ genes have drifted slightly more in sequence than the ‘Same Function’ genes as would be expected if changes in protein function have in part driven the evolution of different organismal functions for these genes. We find that the alignable regions are more divergent (as measured by the Ka or Ka/Ks metrics; see Figure S5A,B, *p*<0.01 Mann Whitney U test) and that both the number and the total length of non-alignable regions are slightly increased (Figure S5C,D, *p*<0.01 Mann Whitney U test). This is consistent with a model in which drift in the proteins encoded by orthologous genes might contribute to DSD, but this effect is modest at this level of bulk analysis. It is nonetheless predictive: the orthologues that differ most in sequence are substantially more likely to have different RNAi phenotypes than more similar orthologues and this is shown in Figure S5E.

We thus find that the greater the divergence in protein sequence between orthologues, the greater the likelihood that they will have different *in vivo* functions, as identified by different RNAi phenotypes. However, we have no conclusive evidence to show that this is causative rather than correlative: it could simply be that genes with differing *in vivo* roles have more rapidly diverging coding sequences and this is still an open question from our data.

### Orthologues may have different organismal roles due to changes in other genes

We tested whether changes in RNAi phenotype might be due either to changes in gene expression or to changes in the molecular functions of the encoded protein. We identified four genes with a different RNAi phenotype between *C. elegans* and *C. briggsae* which is likely to be due to changes in promoter sequence and for one of these, *sac-1*, we showed that to be the case. In addition, given the increased protein divergence between orthologues that have different RNAi phenotypes in the two worms, it appears that many of the molecular events that lead to changes in the level of requirement for a specific gene are likely to be linked to changes in the gene itself, either in its promoter or in its coding region. As shown in Figure 4, there is a final possibility: that orthologues in the two species might encode identical proteins and be expressed in an identical manner, yet still have very different RNAi phenotypes due to changes in other genes that alter the level at which the orthologues are required. In such cases, both the coding regions and the regulatory sequences are functionally interchangeable between the orthologues, but the RNAi phenotypes in the two species still differs. Similar cross-species transgenic approaches have been used to great effect between *C. elegans* and *C. briggsae*. For example, a similar cross species rescue experiment has been used to show that the different RNAi phenotype of *gld-1* lies in the overall genetic context of *C. elegans* and *C. briggsae* and not in the molecular function of *gld-1* [31], [32] and careful analysis of *unc-47* has revealed extensive compensatory evolution in the regulation of gene expression in these two species [33], [34].

**Figure 7:**
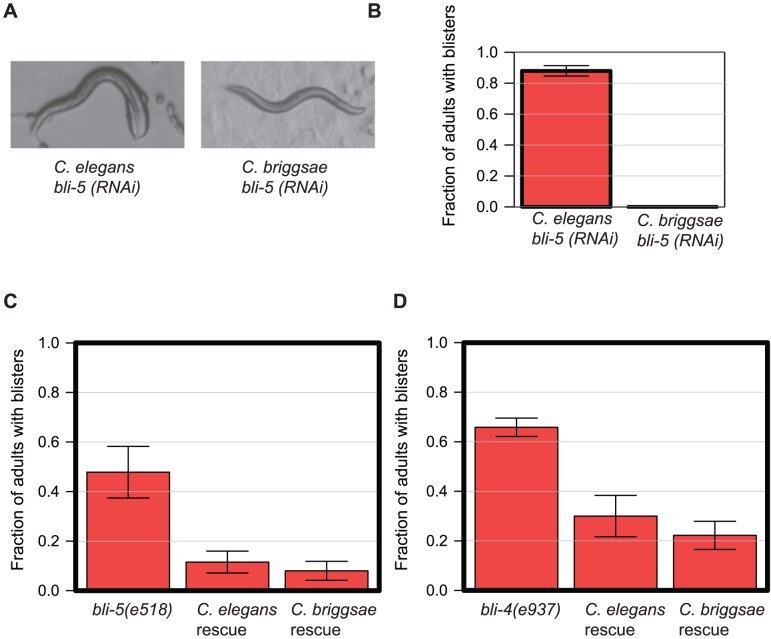
*bli-5* and *bli-4* have an identical gene function despite showing different RNAi phenotypes. **A.** RNAi phenotype of *bli-5* in *C. elegans* (N2) and *C. briggsae* (JU1018). **B.** Quantification of the phenotype shown in panel A. **C.** Rescue of the *bli-5(e518)* phenotype by either *C. elegans* or *C. briggsae bli-5* genes. We generated transgenic *bli-5(e518)* lines in which either *C. elegans bli-5* coding region was expressed under the control of *C. elegans bli-5* promoter (*C. elegans* rescue) or the *C. briggsae bli-5* under the control of the *C. briggsae bli-5* promoter (*C. briggsae* rescue). We examined adult animals and assessed the proportion with blistered cuticles; the results were combined across lines, with a minimum of 3 lines. Error bars represent the standard error on the binomial proportion. **D.** Similar data to panel C but instead showing rescue of the *bli-4(e937)* allele with analogous *C. elegans* and *C. briggsae bli-4* constructs.

We found two examples of orthologues that have differing *in vivo* functions in *C. elegans* and *C. briggsae* due to changes in other genes. *bli-4* and *bli-5* act together to regulate molting and have very different phenotypes in the two species studied — for example *bli-5* has a strong blistering phenotype in *C. elegans* but not in *C. briggsae* (Figure 7A,B). *bli-4* encodes a subtilisin-like serine protease [35] whereas *bli-5* encodes a kunitz family serine protease inhibitor thought to act with BLI-4 [36]. Given that these genes are hypothesized to act together to affect cuticle development, we wondered whether the difference in requirement for these two genes in *C. elegans* and *C. briggsae* might not be due to independent functional changes in *bli-4* and *bli-5*, but to changes in the requirement for this entire pathway between the two worms due to changes in other genes. Using transgenic rescue experiments we found that both the coding sequences and the promoters of *C. elegans* and *C. briggsae* are functionally interchangeable for both *bli-4* and *bli-5*: expression of the *C. briggsae bli-5* under control of the *C. briggsae bli-5* promoter gives as robust rescue of the *C. elegans bli-5(e518)* null mutant as expression of *C. elegans bli-5* coding region under control of the *C. elegans bli-5* promoter (Figure 7C); the same is true for rescue of the *C. elegans bli-4(e937)* mutant by *C. briggsae bli-4* (Figure 7D). Thus, at least in these two cases, we have found examples where the difference in the RNAi phenotype for orthologues in *C. elegans* and *C. briggsae* is not due to any difference in the genes themselves, but rather in the level of requirement for the pathway in which the genes act.

### Conservation of function can be maintained at the level of gene family and not gene family members

In the case of *bli-4* and *bli-5* above, these genes have differing RNAi phenotypes in the two species studied because of changes elsewhere in the genetic networks of these worms but one cannot trivially pinpoint these other changes. However, for a subset of genes with differing phenotypes one can make an educated guess — the set of genes that are members of multigene families. In these cases, it is possible that both worms have an essential requirement for a specific gene activity but that this is carried out by different members of the same gene family in the two worms. Although we have not followed this in depth, we have data that are consistent with this.

We first examined all 91 *C. briggsae* genes that had a weaker phenotype and searched for related genes in the *C. briggsae* genome (see Supplementary Methods for details) that might instead be carrying out the required molecular function. If this is indeed the case, these related genes would thus be expected to have a stronger phenotype in *C. briggsae*. There are 49 genes with a weaker phenotype in *C. briggsae* for which we were able to find one or more related genes in the *C. briggsae* genome that might have a similar molecular function. When we compared RNAi phenotypes in *C. elegans* and *C. briggsae* for these related genes, we find 5 examples where the *C. briggsae* gene has a stronger phenotype than *C. elegans* (Figure S6) — for example *rsp-3* is an SR protein which is 100% embryonic lethal in *C. elegans*, but not in *C. briggsae*, while *rsp-6*, a different SR protein, is 100% embryonic lethal in *C. briggsae* but not *C. elegans*. The family of *rsp* genes is known to have multiple functional overlaps in *C. elegans* [37], [38] and we suggest that not only is this true in *C. briggsae* but, crucially, that the relative importance of each family member differs in the two species. This is consistent with a model in which both *C. elegans* and *C. briggsae* require a specific molecular function, but that this function can be carried out by different members of the same family of genes in the two species.

In summary, we have a generated an RNAi library targeting 1333 *C. briggsae* genes; each targeted gene is the direct orthologue of a *C. elegans* gene known to have a clear detectable RNAi phenotype. We screened for genes that have major differences in *in vivo* function between the two nematodes but clearly many more refined RNAi screens are possible using this reagent and we anticipate that the availability of our library will help drive progress in this area of comparative evolutionary development. We identified 91 genes with obviously different *in vivo* functions and examining these genes reveals key features of the molecular events driving the changes in gene function that accompany DSD. In more focused studies, we showed that multiple genes with different *in vivo* functions have evolved different expression patterns and, in the case of *sac-1*, we showed that promoter evolution is indeed the cause of the change in *in vivo* function. This is only one example and we anticipate that our dataset, along with the RNAi library itself, will provide a rich source of other future detailed studies to pinpoint the molecular causes of the changes in *in vivo* function that we observe.

## Discussion

*C. elegans* and *C. briggsae* are phenotypically extremely similar. They live in the same ecological niche [6], they have near-identical development [7], and are sufficiently morphologically close that they can be crossed and can fertilize each other [10]. The resulting interspecies hybrids are not viable, however, indicating that while the biology of these two nematodes is nearly identical, the molecular pathways that underpin this conserved biology have diverged substantially, a phenomenon termed Developmental System Drift (DSD) [5].

One of the consequences of DSD is that some orthologous genes play different *in vivo* roles in the two species and thus their loss of function phenotypes will be different. Our goal in this study was to investigate the consequences of DSD on gene function in *C. elegans* and *C. briggsae*. Rather than examine one specific process in great detail, as has been done successfully before in these two species [39]–[41], we chose instead to carry out a broad screen to identify as many cases as possible of genes that have different *in vivo* roles due to DSD and hence to gain insight into the following questions. How many genes have changed their *in vivo* roles as these species diverged? Is this common or extremely rare? Do specific classes of genes change more frequently? Finally, can we identify any common features in the molecular events that underlie the changes in gene function that we identify? Addressing these questions gives insight both into how great an impact DSD has on the evolution of gene function and into how gene functions evolve during DSD.

We used RNAi to target over 1300 genes in both *C. elegans* and *C. briggsae*. Each of these genes has a readily detectable RNAi phenotype in *C. elegans* and thus we could identify genes whose RNAi phenotypes (and hence whose *in vivo* functions) differ between these two species as the result of DSD. Using a manual phenotyping method designed to screen for a broad range of phenotypes, we identified 91 orthologues that have obviously different RNAi phenotypes in these two species (the ‘Different Function’ genes). In parallel to this, we also screened 374 genes using an automated quantitative phenotyping method which allows detection of more subtle differences in brood size and growth rate. This more sensitive assay identified significant differences in phenotype for ∼21% of genes. Taken together, we estimate that over 25% of genes have different *in vivo* functions in *C. elegans* and *C. briggsae* as the result of DSD.

We note that this estimate is likely to be a substantial underestimate of the true rate at which gene functions are diverging during DSD for several reasons. Firstly, while we tried hard to eliminate false positives from our dataset, both through multiple rounds of rescreening and by re-designing additional RNAi clones for each potential hit, we have little means to estimate our false negative rate. This is likely to be significant: the screen was carried out at high throughput, the phenotypes examined were fairly crude and, at least in the case of the manual phenotyping, differences needed to be quite large for us to detect. All these factors will result in false negatives and thus the proportion of the genes that we screened which have truly different phenotypes is almost certainly higher than we report here. Secondly, because of the difference in RNAi efficacy in the two species, we could only detect biologically meaningful differences in RNAi phenotype if the phenotype was weaker in *C. briggsae* than in *C. elegans*. In all likelihood, there are as many genes that have a weaker phenotype in *C. elegans* than in *C. briggsae* as *vice versa*, we just cannot identify them in our screen. Finally, we screened an extremely selectively chosen gene set i.e. the set of genes that have a readily detectable RNAi phenotype in *C. elegans* (<15% of all *C. elegans* genes [14]) and that also have a 1∶1 orthologue in *C. briggsae*. While only ∼60% of genes have a 1∶1 orthologue in *C. elegans* and *C. briggsae* [9], our gene set is extremely highly conserved: ∼90% have 1∶1 orthologues between these two species. Furthermore, many of the genes we screened are known to be functionally conserved over extremely long evolutionary distances: for example, 60% of the genes giving lethal or sterile phenotypes in *C. elegans* are also essential for viability in *S. cerevisiae* [26]. The set of genes we screened are likely to be the most functionally conserved between *C. elegans* and *C. briggsae* of any genes in the genome. Taking this all together, our finding that during DSD over 25% of these have evolved different functional roles in the two species is surprisingly high and suggests that DSD has a major impact on the evolution of gene function.

What are the underlying molecular causes of the differences in gene function that we observe as differences in RNAi phenotype? We find that three main types of molecular events explain many of the changes in gene function that we identified.

Firstly, we find multiple examples in which orthologous genes that have different RNAi phenotypes also have different *in vivo* expression patterns. We examined the expression patterns of 22 such ‘Different Function’ genes in both species and find that 4 have a clearly different expression pattern in *C. elegans* and *C. briggsae* that is entirely due to promoter evolution. In all four cases, the species in which the RNAi phenotype is less penetrant (and thus the species which has a lower requirement for the function of that orthologue) is also the species in which the expression is far more restricted, suggesting that the difference in phenotype might indeed be explained by the difference in expression. In one case, *sac-1*, we tested this explicitly and showed that this is indeed true. Gene expression, as a result of promoter evolution, thus plays a significant role in the way genes change *in vivo* functions during DSD.

Secondly, certain types of gene are more likely than others to evolve different *in vivo* functions as a result of DSD. While most of the core conserved components of the eukaryotic cell (the ribosome, the proteasome etc.) tend to have the same functions in both species, transcription factors and recently evolved genes of unknown function often have different phenotypes. In the case of transcription factors, this result is perhaps expected: transcriptional networks are known to be extremely plastic and can rewire extensively while still having similar outputs and responses [42]. For the recently evolved genes, however, this is intriguing. None of them have orthologues outside nematodes and indeed many are specific to *Caenorhabditis* species, and few have any functional annotation. Why should a gene that is absolutely essential for *C. elegans* viability be more likely to be dispensable in *C. briggsae* if it evolved recently than if it is an ancient gene? What essential roles do these novel genes play in nematode biology and why do they seem to be changing so rapidly? Some carefully dissected examples already exist such as the example of *fog-2* and *she-1* in the independent evolution of hermaphroditism in these two species. *fog-2* is a recently evolved gene which has evolved a specific function in sperm development in *C. elegans*, while the non-orthologous F-box protein *she-1* plays the same role in *C. briggsae* [43]–[45]. The roles of such novel recently evolved genes in nematode biology and evolution are intriguing open questions that will require extensive follow-up studies.

Finally, our data suggest that the individual members of multigene families frequently adopt different *in vivo* roles during DSD. There are often multiple redundancies among members of gene families and we suggest that this results in the requirement for any single family member to be extremely fluid over time. For example, there are well described redundancies in the SR family of splicing regulators in *C. elegans* [37], [38]. We find that while *rsp-3* is essential for viability of *C. elegans*, targeting *rsp-3* in *C. briggsae* has little effect; conversely, targeting *rsp-6* in *C. briggsae* has a strong RNAi phenotype, but in *C. elegans rsp-6* has no obvious phenotype. In this example, while both worms require an *rsp* activity, in *C. elegans* the essential *rsp* is *rsp-3* whereas in *C. briggsae* it is *rsp-6* and we suggest this is a common feature of drift in gene function during DSD.

We note that all the three key molecular drivers of gene functional change during DSD — changes in gene expression, the rapid evolution of novel genes, and subfunctionalisation among related family members — are also central molecular drivers of changes in gene function that result in adaptation [4], [29], [46]. One explanation for this is that DSD and adaptation are unrelated and unlinked phenomena— for example, some evolved alterations in gene expression have advantageous phenotypic outcomes while others have no impact on phenotype and neither set of changes has any influence on the other. While this is completely plausible, there is an alternative view: that the reason that the molecular events that often underpin the changes in gene function that accompany both DSD and adaptation are very similar is that DSD and adaptation are intimately linked evolutionary phenomena.

One possible conceptual model for a link between DSD and adaptation comes from detailed studies of *in vitro* molecular evolution [47], [48]. In these studies, the evolution of a new phenotype (in this case, a new fold or activity) is rarely the result of a single adaptive mutation alone. Rather, a series of phenotypically neutral mutations (the molecular equivalent of DSD) results in a derived molecule that is phenotypically indistinguishable from the ancestor, but that is different with respect to its evolvability. While a final adaptive mutation results in a new adaptive phenotype in the derived molecule, making the same mutation has no effect on the phenotype of the ancestral form. The derived and ancestral molecules are thus functionally equivalent, but a single base change has radically different phenotypic consequences for adaptation in these two molecular species. In this way, at least at the level of adaptation of *in vitro* molecular phenotypes, neutral drift and adaptation are often intimately linked. We speculate that DSD and adaptation might be linked in an analogous manner at the level of whole organism phenotypes.

While the widespread changes in gene function that occur during DSD do not appear to have any direct impact on phenotype, they might have profound consequences on the effect of additional subsequent changes. The effect of DSD, viewed in this way, is that while two species such as *C. elegans* and *C. briggsae* are phenotypically extremely similar at present, the possible evolutionary trajectories of the two species are very different since the phenotypic outcomes of identical molecular changes can be very different in the two animals. Changes in gene function that would be deleterious in *C. elegans* might have no effect in *C. briggsae* (e.g. mutation or change in gene expression of *sac-1*) or, at the limit, might confer a selective advantage that would drive adaptation. This idea of a potential link between DSD and adaptation is still speculative but the finding we report here that similar molecular events underlie the evolution of gene function in both processes is consistent with this notion.

In summary, then, we used RNAi to identify genes with different *in vivo* functions in two extremely phenotypically similar nematode worms, *C. elegans* and *C. briggsae*. This study is the first systematic survey of the outcome of DSD on the *in vivo* functions of orthologous genes in any closely-related animal species and our data suggest that DSD has major consequences for the evolution of gene function. We anticipate that the dataset from our RNAi screen will help to drive deeper characterization of the molecular events underlying DSD and, just as the public availability of the *C. elegans* RNAi library was key for the systematic analysis of gene function in *C. elegans*, so the availability of the *C. briggsae* RNAi library will drive extensive comparative screens in these two related nematodes.

## Materials and Methods

### Construction of the *C. briggsae* RNAi library

We used InParanoid 6.1 [20] to identify *C. briggsae* genes that are putative 1∶1 orthologues of *C. elegans* with a reported RNAi phenotype [14]. To further validate these orthologue assignments, we also used orthology assignments from TreeFam, which use phylogeny relationships, and also synteny to resolve complex orthologue assignments. Full details are given in Table S1. In order to design the *C. briggsae* clones we identified the orthologous region in the *C. briggsae* genome to that targeted by the *C. elegans* RNAi clone using BLAST and used this as a seed region. Predicted clones that had at least 80% identity over 200 bp to additional *C. briggsae* genes were eliminated as having potential off target effects and manually redesigned. Secondary clones were designed by hand according to the principles above and were targeted to a separate group of exons to the first clone we used.

For cloning we digested L4440 with EcoRV (Fermentas) and then dephosphorylated with Shrimp Alkaline Phosphatase (Fermentas). PCR products were amplified from AF16 genomic DNA using Pfu (Fermentas) and then phosphorylated with PolyNucleotide Kinase (Neb) for blunt end cloning. The vector and PCR products were ligated together overnight and then transformed into HT115 bacteria. The colonies were screened using a T7 colony PCR, and positives were reassembled into the correct locations in 96 well plates, and then finally verified using an insert specific colony PCR.

### Manual screening of the *C. briggsae* RNAi library

*Caenorhabditis* species were maintained by feeding on OP50 on NGM plates at 20°C. Screening was done on 12 well agar plates as previously described [14]. We screened for a list of visible phenotypes which have been previously reported [14], listed here: Emb (embryonic lethal), Ste (sterile), Stp (sterile progeny), Gro (slow post-embryonic growth), Lva (larval arrest), Lvl (larval lethality), Adl (adult lethal), Bli (blistering of cuticle), Bmd (body morphological defect), Clr (clear), Dpy (dumpy), Egl (egg-laying defective), Him (high incidence of males), Lon (long), Mlt (moult defect), Muv (multivulva), Prz (paralysed), Pvl (protruding vulva), Rol (roller), Rup (ruptured), Sck (sick) and Unc (uncoordinated). Each orthologue pair was screened by 2 people in 2 fully independent experimental set-ups on separate weeks. Our confidence score is the number of observations of a phenotype difference out of 4 possible observations. Genes with at least 3 out of 4 observations of a different phenotype in the two species were potential hits and were tested in secondary screens. For these, we designed additional RNAi clones which targeted a different region of the *C. briggsae* gene where possible and screened these secondary RNAi clones in an identical way to the first screen. Genes were called as final hits if we saw a consistent phenotype difference using both the primary and secondary RNAi clones.

### Fitness assay

L1 animals were grown and filtered for purification as described above. RNAi clones were grown overnight at 37°C in LB media with 1 mM Carbenicillin and induced at a final concentration of 4 µM IPTG for one hour. After induction, bacterial cultures were spun down and resuspended in NGM containing 4 µM IPTG and 1 mM Carbenicillin. 10 µl of a ∼1 worm/µl solution were put into each well of a 96 well plate and then 40 µl of the bacterial suspension was added. Each row of the 96 well plate had 5 replicates of each RNAi clone for each species and 2 blank wells. In each plate non-targeting dsRNA-expressing bacteria (GFP) were also present as negative controls. After growing at 20°C with shaking at 200 rpm for 96 hours we quantified the number of progeny using a COPAS worm sorter; the length (measured as the Time of Flight -TOF) and optical darkness (measured as Extinction - EXT) of each counted animal are also recorded.

From these data, we calculated the relative brood number following RNAi as the ratio between the worm number in the targeted cultures and the worm number in cultures grown with non-targeting GFP RNAi bacterial controls. To assess differences in relative brood size, we calculated the log ratio of the relative brood sizes for *C. elegans* and *C. briggsae* for each targeted gene, and used the empirical distribution of 60 independent non-targeting GFP RNAi bacterial controls to determine a cutoff for statistical significance. In order to identify embryonic lethal phenotypes we counted objects with TOF less than 50 and EXT less than 30 (which identifies embryos) and calculated the ratio of the number of embryos to non-embryos for each RNAi and control experiment. By comparing the empirical distribution of these ratios in the control experiments to the targeting RNAi we were able to identify genes that resulted in embryonic lethality when knocked down by RNAi.

### qPCR

For each knock down, 50 L4 larvae were grown on a lawn of dsRNA-expressing bacteria on NGM plates containing 1 mM IPTG and 1 mM Carbenicillin for 72 hr. RNA was harvested using Trizol (Invitrogen) and was cleaned-up using an RNeasy kit (QIAGEN). Following a DNase I digestion (Invitrogen) we carried out first strand cDNA synthesis using superscript II (Invitrogen). We calculated the efficiency of the primers by dilution curves and ensured they were between 1.85 and 2.05. The qPCR was done in a CFX96 (Bio-Rad) using Sybr Green (Clonetech) according to the manufacturer's protocols. Relative expression was calculated using the Pfaffl efficiency correction [49] where each sample was normalized to the expression of *tbg-1*.

### Examination of *C. elegans* and *C. briggsae* gene phylogenetic age

In order to define the phylogenetic position of genes we took curated lists of orthologues to the *C. elegans* gene from Wormbase (WS233) [50]. We downloaded a phylogenetic tree from the NCBI taxonomy database [41], (downloaded on the 8^th^ of January 2013) for the species which have genomes available and found the last common ancestor as the point of emergence of each gene.

### GFP stitching and microscopy

PCR primers were designed to amplify 2 kb upstream of the translation start site or up until the next gene. *C. elegans* promoters were combined through PCR stitching to the coding sequence from GFP and *unc-54* 3′UTR from the vector pPD95.75, while *C. briggsae* promoters were stitched onto the coding sequence from mWormCherry and *unc-54* 3′ UTR from the vector pJH1774. Stitched PCR products were quantified on an agarose gel and then diluted to the same concentration and injected with pRF4 into *C. elegans* (N2) worms as a co-injection marker. F2 animals were isolated and then imaged on a custom Quorum confocal microscope. For each expression pattern we imaged a minimum of 3 lines to ensure we had consistent expression patterns. Any genes with obvious expression differences were then validated by injection into *C. briggsae* (AF16) in order to ensure that we get a consistent expression pattern.

### Transgenic rescue experiments

We created the rescue constructs shown schematically in Figure 4 by first generating constructs that encode a C-terminal GFP fusion for each ORF to be expressed using the pPD95.75 vector. For each of these we cloned the region upstream to either *C. elegans* or *C. briggsae* orthologues to make a total of 4 constructs, 2 containing DNA specific to one species, and the other 2 being hybrids between species (shown in Figure 4). The *bli-4* and *bli-5* constructs were injected at 15 ng/µl with pCFJ90 as a co-injection marker. We isolated F2 progeny which were positive for *myo-2*::mCherry and then we counted the proportion of RFP+ adult animals with blisters. The *sac-1* constructs were injected at 1 ng/µl with pRF4 as a co-injection marker into *sac-1(ok1602)* animals. Rol positive F2s were isolated and the proportion of homozygous adult rescued animals were scored by the absence of *myo-2* GFP signal from the hT2 balancer. A subset of animals were confirmed to be homozygous by single worm genotyping PCR.

### Examination of *C. elegans* and *C. briggsae* protein similarity

Orthologues between *C. elegans* and *C. briggsae* were defined using InParanoid 6.1 and their CDS sequences were downloaded from Wormbase (WS190). We translated these to protein sequences, aligned them using ClustalW 2.0 [51] and then projected these alignments back to the CDS sequences. We then used the Yn00 program from PAML (4.3) [52] to calculate Ka, Ks and the Ka/Ks ratios for *C. elegans* and *C. briggsae* orthologues. We measured evolutionary novel segments between *C. elegans* and *C. briggsae* by taking the protein alignments defined above and then identifying segments which did not align between the 2 species (minimum of 4 residues). We then counted the total number of such unique segments as well as the total residues involved.

### Predictability of phenotype differences

In this procedure we ranked orthologues by either the Ka metric. Then we randomly picked pairs of orthologues, one with a different phenotype and one with the same phenotype, and we asked whether the orthologue with a greater Ka was the orthologue with a different phenotype. If so we classified this as a positive prediction and put it into bins based on the rank difference of the Ka. This randomization procedure was repeated one million times and the results were plotted.

### Identifying functionally related genes

We identified genes which have weaker RNAi phenotypes in *C. briggsae* and then searched for related *C. briggsae* genes by using BLASTP; we considered any gene with a BLASTP hits with an E-value less than 10^−5^ as a possible related gene. We then constructed RNAi clones for the sets of related genes but excluded families with greater than five related members as being too complex. All RNAi clones were screened in *C. briggsae* side by side with RNAi experiments in *C. elegans* using clones targeting the *C. elegans* orthologues. In this way we compared the RNAi phenotypes of *C. elegans* and *C. briggsae* orthologues for small gene families that contain at least one member that had a weaker phenotype in *C. briggsae*.

## Supporting Information

Figure S1 Design of the *C. briggsae* RNAi library. **A.** 1640 genes with an RNAi phenotype in *C. elegans* were targeted and then filtered for having a 1∶1 orthologue in *C. briggsae*. **B.** *C. elegans* RNAi clones form the Ahringer library were mapped by BLAST to the *C. briggsae* genome and used as a seed region for RNAi clone design. Primers were designed around this region targeting the maximum number of bases of exons.
(EPS)

Figure S2 The breakdown of the final screening results we observed. **A.** Breakdown of primary RNAi screen. Genes were placed into three classes: those with an identical phenotype in both species (‘Identical’); those with a stronger phenotype in *C. briggsae* (JU1018) (‘Stronger’); and those with a weaker phenotype in *C. briggsae* (JU1018) (‘Weaker’). **B.** **Breakdown of the final result after rescreening with secondary RNAi constructs.**
(EPS)

Figure S3 Comparison of levels of knock-down achieved in *C. elegans* and *C. briggsae* using bacterial-mediated RNAi. 9 genes were individually targeted by RNAi in both *C. elegans* and *C. briggsae*, RNA was harvested after 72 hrs of treatment, and qPCR was used to examine levels of knockdown. One of the genes had an identical RNAi phenotype in the two nematodes, the other 8 had weaker RNAi phenotypes in *C. briggsae* as indicated in the figure. The data in the graph represent the means of three independent biological replicates; each biological replicate had two independent technical replicates. The error bars shown are the standard error and expression levels are expressed relative to the expression of *tbg-1*. Genes are from left to right *pqn-85,nekl-2,K04G7.1,csn-5,unc-62*, *mcm-7*, *sac-1*, *apr-1*, *tsr-1*.
(EPS)

Figure S4 The genomic contexts of *C. elegans sac-1* and *C. briggsae sac-1*. Tracks were downloaded from wormbase version WS235 and the cloned upstream region is highlighted in red.
(EPS)

Figure S5 Enrichment of metrics relating to protein divergence for genes with a different phenotype. **A.,B.** Ka (Non-synonymous substitutions per non-synonymous site) and Ka/Ks ratio was calculated from the Yn00 program of PAML 4.3 **C.,D.** The number of non-aligned regions is calculated by counting the number of gaps (minimum size of 4 residues) in the protein alignments, and the total number of residues in all of those gaps. **E.** Ka is predictive of a different phenotype when the difference in large. Genes were ranked by the Ka metric and random pairs were chosen, one with the same phenotype and one with a different phenotype. If a Ka in *C.elegans* predicted the gene with a different phenotype, then this is classified as a positive prediction.
(EPS)

Figure S6 Comparison of phenotypes of individual members of multigene families in *C. elegans* and *C.briggsae*. We searched for putative related family members of genes that had weaker RNAi phenotypes in *C. briggsae* by identifying any other *C. briggsae* genes that had a BLAST evalue cutoff of 10^−5^ or better, up to a maximum of 5 related proteins — larger families were excluded from our analysis due to their complexity. These genes and their *C.elegans* orthologs were aligned and a gene tree was constructed with the dnaml program in phylip. RNAi phenotypes are shown in bold at the tips of the tree; if no phenotype in indicated then the gene is wildtype. Abbreviations are as follows – Wt – wildtype, Gro – Growth defective, Emb – Embryonic Lethal, Lvl – Larval lethal.
(EPS)

Table S1 Validation of the orthology assignments. We used InParanoid 6.1 to identify putative orthologues between *C. elegans* and *C. briggsae*. To determine if there were additional possible alternative BLAST hits in either genome that could confound correct orthologue assignments, we examined the E-values of the next best hits in either genome. If the E-values of next best hits in either genome were greater than 10^20^ higher (i.e. worse match) than the best hits, we called this an unambiguous hit and gave this a confidence value of 3, indicating that these orthologues are the sole similar genes in either genome. If the situation was more complex, we examined both tree-based and synteny-based methods to resolve true orthology — if both synteny and tree-based methods confirm the initial orthologue assignment, it has a confidence value of 2, if it is supported by only one line of evidence, it scores only 1. If we can find no support for the orthologue assignment from either synteny or tree-based approaches, we score this as a zero — we note that only 9 genes in total fell in this category. Thus over 99% of all the InParanoid orthologue assignments could be validated by one or more independent orthology assignment methods. For the tree-based orthology, we used the precomputed data in TreeFam [22]. For synteny, we defined syntenic genes if the *C. briggsae* orthologue of either of the two upstream genes was found up to 2 genes adjacent to the *C. elegans* gene, or similarly for the downstream gene.
(PDF)

Table S2 Genes identified by eye as having a different phenotype between *C. elegans* and *C. briggsae*. The number of supporting observations in the primary screen (out of 4) and the secondary screen (out of 2) is shown. Functional annotations are from the manual annotation in Kamath *et al.*
(PDF)

Table S3 Quantitative analysis of brood size RNAi phenotypes for genes screened on the worm sorter in *C. elegans* and *C. briggsae*. Shown are the brood size scores as described in the methods section as well as an empirical p-value calculated from the distribution of GFP negative control RNAi replicates.
(PDF)
