# Supplementary material for: Comparative RNAi Screens in C. elegans and C. briggsae Reveal the Impact of Developmental System Drift on Gene Function
Source: PLoS Genet. 2014 Feb 6;10(2):e1004077. doi: 10.1371/journal.pgen.1004077 (PMC3916228; doi:10.1371/journal.pgen.1004077)
Supplement: Table S1 — Validation of the orthology assignments. We used InParanoid 6.1 to identify putative orthologues between C. elegans and C. briggsae. To determine if there were additional possible alternative BLAST hits in either genome that could confound correct orthologue assignments, we examined the E-values of the next best hits in either genome. If the E-values of next best hits in either genome were greater than 1020 higher (i.e. worse match) than the best hits, we called this an unambiguous hit and gave this a confidence value of 3, indicating that these orthologues are the sole similar genes in either genome. If the situation was more complex, we examined both tree-based and synteny-based methods to resolve true orthology — if both synteny and tree-based methods confirm the initial orthologue assignment, it has a confidence value of 2, if it is supported by only one line of evidence, it scores only 1. If we can find no support for the orthologue assignment from either synteny or tree-based approaches, we score this as a zero — we note that only 9 genes in total fell in this category. Thus over 99% of all the InParanoid orthologue assignments could be validated by one or more independent orthology assignment methods. For the tree-based orthology, we used the precomputed data in TreeFam [22]. For synteny, we defined syntenic genes if the C. briggsae orthologue of either of the two upstream genes was found up to 2 genes adjacent to the C. elegans gene, or similarly for the downstream gene. (PDF) [file pgen.1004077.s007.pdf]

Gene(WBID)	Gene(Common)	*C. briggsae* ortholog	Confidence Score	Difference in -log10 BLAST E-values	BLAST proteome result	Synteny result	Treefam result	Different Phenotype?
WBGene00016323	*C32E8.5*	CBG12001	3	114.79588	Unambiguous reciprocal BLAST	Supported by synteny	Confirmed by Treefam	Different Phenotype
WBGene00017852	*F27C1.2*	CBG12308	3	107.3679768	Unambiguous reciprocal BLAST	Supported by synteny	Confirmed by Treefam	Different Phenotype
WBGene00000814	*csn-2*	CBG22097	3	101.8239087	Unambiguous reciprocal BLAST	Supported by synteny	Confirmed by Treefam	Different Phenotype
WBGene00022631	*nekl-2*	CBG12695	3	107	Unambiguous reciprocal BLAST	Supported by synteny	Confirmed by Treefam	Different Phenotype
WBGene00000431	*ceh-6*	CBG12531	3	Inf	Unambiguous reciprocal BLAST	Supported by synteny	Confirmed by Treefam	Different Phenotype
WBGene00009264	*sac-1*	CBG24498	3	Inf	Unambiguous reciprocal BLAST	Supported by synteny	Confirmed by Treefam	Different Phenotype
WBGene00003904	*pabp-2*	CBG03741	3	71.82390874	Unambiguous reciprocal BLAST	Supported by synteny	Confirmed by Treefam	Different Phenotype
WBGene00003209	*mel-26*	CBG12451	3	Inf	Unambiguous reciprocal BLAST	Supported by synteny	Confirmed by Treefam	Different Phenotype
WBGene00007275	*C03D6.1*	CBG03701	3	114	Unambiguous reciprocal BLAST	Supported by synteny	Not confirmed by Treefam	Different Phenotype
WBGene00003123	*mag-1*	CBG18690	3	104.6690068	Unambiguous reciprocal BLAST	Supported by synteny	Confirmed by Treefam	Different Phenotype
WBGene00012235	*W04A4.6*	CBG13613	3	74.81291336	Unambiguous reciprocal BLAST	Supported by synteny	Not confirmed by Treefam	Different Phenotype
WBGene00021465	*Y39G10AR.7*	CBG03924	3	134.544068	Unambiguous reciprocal BLAST	Not supported by synteny	Confirmed by Treefam	Different Phenotype
WBGene00022027	*vps-20*	CBG05144	3	112.69897	Unambiguous reciprocal BLAST	Supported by synteny	Confirmed by Treefam	Different Phenotype
WBGene00022117	*Y71F9AL.12*	CBG04229	3	106.30103	Unambiguous reciprocal BLAST	Supported by synteny	Confirmed by Treefam	Different Phenotype
WBGene00020149	*T01D1.4*	CBG06970	3	60	Unambiguous reciprocal BLAST	Supported by synteny	Confirmed by Treefam	Different Phenotype
WBGene00006647	*tsr-1*	CBG20440	3	Inf	Unambiguous reciprocal BLAST	Not supported by synteny	Confirmed by Treefam	Different Phenotype
WBGene00017916	*F29A7.6*	CBG19575	3	44	Unambiguous reciprocal BLAST	Supported by synteny	Confirmed by Treefam	Different Phenotype
WBGene00015298	*C01F1.3*	CBG03656	3	Inf	Unambiguous reciprocal BLAST	Supported by synteny	Confirmed by Treefam	Different Phenotype
WBGene00018961	*F56D1.3*	CBG02407	3	86.18563658	Unambiguous reciprocal BLAST	Supported by synteny	Confirmed by Treefam	Different Phenotype
WBGene00019126	*F59E12.11*	CBG02460	3	133.30103	Unambiguous reciprocal BLAST	Supported by synteny	Confirmed by Treefam	Different Phenotype
WBGene00004198	*prx-13*	CBG13114	3	164.5228787	Unambiguous reciprocal BLAST	Supported by synteny	Confirmed by Treefam	Different Phenotype
WBGene00014066	*rev-1*	CBG03293	3	Inf	Unambiguous reciprocal BLAST	Supported by synteny	Confirmed by Treefam	Different Phenotype
WBGene00010941	*M176.2*	CBG13395	3	Inf	Unambiguous reciprocal BLAST	Supported by synteny	Confirmed by Treefam	Different Phenotype
WBGene00009504	*F37B12.1*	CBG03244	3	117.6532125	Unambiguous reciprocal BLAST	Supported by synteny	Not confirmed by Treefam	Different Phenotype
WBGene00018793	*F54C4.1*	CBG00465	3	102.4149733	Unambiguous reciprocal BLAST	Supported by synteny	Confirmed by Treefam	Different Phenotype
WBGene00003912	*pal-1*	CBG17980	3	115.5228787	Unambiguous reciprocal BLAST	Supported by synteny	Not confirmed by Treefam	Different Phenotype
WBGene00016442	*C35D10.5*	CBG17958	3	137.845098	Unambiguous reciprocal BLAST	Supported by synteny	Confirmed by Treefam	Different Phenotype
WBGene00016169	*C27F2.7*	CBG18209	3	Inf	Unambiguous reciprocal BLAST	Supported by synteny	Confirmed by Treefam	Different Phenotype
WBGene00001662	*gop-3*	CBG21181	3	Inf	Unambiguous reciprocal BLAST	Supported by synteny	Confirmed by Treefam	Different Phenotype
WBGene00015146	*abi-1*	CBG08994	3	Inf	Unambiguous reciprocal BLAST	Supported by synteny	Confirmed by Treefam	Different Phenotype
WBGene00004857	*sma-3*	CBG16541	3	Inf	Unambiguous reciprocal BLAST	Not supported by synteny	Confirmed by Treefam	Different Phenotype
WBGene00004450	*rpl-36*	CBG08350	3	66.42596873	Unambiguous reciprocal BLAST	Supported by synteny	Confirmed by Treefam	Different Phenotype
WBGene00019400	*K04G7.1*	CBG16609	3	Inf	Unambiguous reciprocal BLAST	Supported by synteny	Not confirmed by Treefam	Different Phenotype
WBGene00017358	*F10E9.7*	CBG16641	3	41.80163235	Unambiguous reciprocal BLAST	Supported by synteny	Not confirmed by Treefam	Different Phenotype
WBGene00019455	*K06H7.1*	CBG09232	3	76.52287875	Unambiguous reciprocal BLAST	Not supported by synteny	Not confirmed by Treefam	Different Phenotype
WBGene00004201	*prx-19*	CBG06892	3	161.8573325	Unambiguous reciprocal BLAST	Supported by synteny	Confirmed by Treefam	Different Phenotype
WBGene00014229	*ZK1128.3*	CBG09871	3	136.0511525	Unambiguous reciprocal BLAST	Supported by synteny	Not confirmed by Treefam	Different Phenotype
WBGene00004735	*sbp-1*	CBG13284	3	Inf	Unambiguous reciprocal BLAST	Supported by synteny	Confirmed by Treefam	Different Phenotype
WBGene00004705	*rsp-8*	CBG13299	3	47.30103	Unambiguous reciprocal BLAST	Supported by synteny	Confirmed by Treefam	Different Phenotype
WBGene00004700	*rsp-3*	CBG03563	3	21.77815125	Unambiguous reciprocal BLAST	Supported by synteny	Confirmed by Treefam	Different Phenotype
WBGene00018144	*F37C4.4*	CBG10738	3	151.1303338	Unambiguous reciprocal BLAST	Supported by synteny	Confirmed by Treefam	Different Phenotype
WBGene00000817	*csn-5*	CBG19889	3	Inf	Unambiguous reciprocal BLAST	Not supported by synteny	Confirmed by Treefam	Different Phenotype
WBGene00004374	*rme-2*	CBG03794	3	Inf	Unambiguous reciprocal BLAST	Not supported by synteny	Confirmed by Treefam	Different Phenotype
WBGene00002152	*iars-1*	CBG23754	3	Inf	Unambiguous reciprocal BLAST	Supported by synteny	Not confirmed by Treefam	Different Phenotype
WBGene00012704	*Y39C12A.1*	CBG06255	3	88.77815125	Unambiguous reciprocal BLAST	Supported by synteny	Confirmed by Treefam	Different Phenotype
WBGene00012885	*Y45F10D.4*	CBG22375	3	93.17609126	Unambiguous reciprocal BLAST	Supported by synteny	Confirmed by Treefam	Different Phenotype
WBGene00021365	*smgl-2*	CBG08291	3	Inf	Unambiguous reciprocal BLAST	Supported by synteny	Confirmed by Treefam	Different Phenotype
WBGene00020705	*T22H9.1*	CBG01378	3	178.7781513	Unambiguous reciprocal BLAST	Supported by synteny	Confirmed by Treefam	Different Phenotype
WBGene00003159	*mcm-7*	CBG21868	3	Inf	Unambiguous reciprocal BLAST	Supported by synteny	Confirmed by Treefam	Different Phenotype
WBGene00017769	*F25B4.6*	CBG09344	3	Inf	Unambiguous reciprocal BLAST	Supported by synteny	Confirmed by Treefam	Different Phenotype
WBGene00001345	*fos-1*	CBG09268	3	113	Unambiguous reciprocal BLAST	Supported by synteny	Confirmed by Treefam	Different Phenotype
WBGene00018492	*F46E10.11*	CBG18960	3	96.60205999	Unambiguous reciprocal BLAST	Supported by synteny	Confirmed by Treefam	Different Phenotype
WBGene00011538	*T06E6.1*	CBG05554	3	Inf	Unambiguous reciprocal BLAST	Not supported by synteny	Confirmed by Treefam	Different Phenotype
WBGene00013585	*cyp-42A1*	CBG20323	3	Inf	Unambiguous reciprocal BLAST	Supported by synteny	Confirmed by Treefam	Different Phenotype
WBGene00004786	*sex-1*	CBG17178	3	103.39794	Unambiguous reciprocal BLAST	Supported by synteny	Confirmed by Treefam	Different Phenotype
WBGene00004271	*rab-7*	CBG20853	3	115.4259687	Unambiguous reciprocal BLAST	Supported by synteny	Confirmed by Treefam	Different Phenotype
WBGene00004430	*rpl-18*	CBG22372	3	118.845098	Unambiguous reciprocal BLAST	Supported by synteny	Confirmed by Treefam	Different Phenotype
WBGene00017982	*F32D1.2*	CBG21867	3	27.79835464	Unambiguous reciprocal BLAST	Supported by synteny	Not confirmed by Treefam	Different Phenotype
WBGene00004197	*prx-12*	CBG17349	3	Inf	Unambiguous reciprocal BLAST	Supported by synteny	Confirmed by Treefam	Different Phenotype

WBGene00009259	*F29G6.3*	CBG17311	3	Inf	Unambiguous reciprocal BLAST	Supported by synteny	Not confirmed by Treefam	Different Phenotype
WBGene00001465	*flr-1*	CBG05329	3	Inf	Unambiguous reciprocal BLAST	Not supported by synteny	Confirmed by Treefam	Different Phenotype
WBGene00009626	*F42A8.1*	CBG00873	3	Inf	Unambiguous reciprocal BLAST	Supported by synteny	Confirmed by Treefam	Different Phenotype
WBGene00001086	*dpy-27*	CBG20143	3	Inf	Unambiguous reciprocal BLAST	Not supported by synteny	Confirmed by Treefam	Different Phenotype
WBGene00000255	*bli-5*	CBG21218	3	110.5228787	Unambiguous reciprocal BLAST	Not supported by synteny	Not confirmed by Treefam	Different Phenotype
WBGene00017853	*F27C1.3*	CBG12311	2	11.69897	Possible alternative BLAST hit	Supported by synteny	Confirmed by Treefam	Different Phenotype
WBGene00000254	*bli-4*	CBG12593	2	0	Possible alternative BLAST hit	Supported by synteny	Confirmed by Treefam	Different Phenotype
WBGene00000079	*adr-1*	CBG19440	2	0	Possible alternative BLAST hit	Supported by synteny	Confirmed by Treefam	Different Phenotype
WBGene00000156	*apr-1*	CBG08224	2	0	Possible alternative BLAST hit	Supported by synteny	Confirmed by Treefam	Different Phenotype
WBGene00001979	*hmp-2*	CBG19745	2	0	Possible alternative BLAST hit	Supported by synteny	Confirmed by Treefam	Different Phenotype
WBGene00016020	*sptl-1*	CBG06999	2	0	Possible alternative BLAST hit	Supported by synteny	Confirmed by Treefam	Different Phenotype
WBGene00008166	*saps-1*	CBG02933	2	0	Possible alternative BLAST hit	Supported by synteny	Confirmed by Treefam	Different Phenotype
WBGene00003651	*nhr-61*	CBG04240	2	0	Possible alternative BLAST hit	Supported by synteny	Confirmed by Treefam	Different Phenotype
WBGene00012803	*Y43F4B.5*	CBG03574	2	0	Possible alternative BLAST hit	Supported by synteny	Confirmed by Treefam	Different Phenotype
WBGene00006914	*vha-5*	CBG01592	2	0	Possible alternative BLAST hit	Supported by synteny	Confirmed by Treefam	Different Phenotype
WBGene00021626	*Y47D7A.14*	CBG24582	2	0	Possible alternative BLAST hit	Supported by synteny	Confirmed by Treefam	Different Phenotype
WBGene00008670	*F11A3.2*	CBG19294	2	0	Possible alternative BLAST hit	Supported by synteny	Confirmed by Treefam	Different Phenotype
WBGene00004855	*sma-1*	CBG23326	2	0	Possible alternative BLAST hit	Supported by synteny	Confirmed by Treefam	Different Phenotype
WBGene00004951	*spc-1*	CBG14139	2	0	Possible alternative BLAST hit	Supported by synteny	Confirmed by Treefam	Different Phenotype
WBGene00022201	*Y71H10B.1*	CBG02038	2	0	Possible alternative BLAST hit	Supported by synteny	Confirmed by Treefam	Different Phenotype
WBGene00001824	*hbl-1*	CBG14758	2	0	Possible alternative BLAST hit	Supported by synteny	Confirmed by Treefam	Different Phenotype
WBGene00004194	*prx-5*	CBG11123	2	0	Possible alternative BLAST hit	Supported by synteny	Confirmed by Treefam	Different Phenotype
WBGene00000675	*col-101*	CBG22542	2	0	Possible alternative BLAST hit	Supported by synteny	Confirmed by Treefam	Different Phenotype
WBGene00009880	*F49C12.11*	CBG21705	2	13.36172784	Possible alternative BLAST hit	Supported by synteny	Confirmed by Treefam	Different Phenotype
WBGene00001130	*dyn-1*	CBG07725	2	0	Possible alternative BLAST hit	Supported by synteny	Confirmed by Treefam	Different Phenotype
WBGene00001980	*hmr-1*	CBG07964	1	0	Possible alternative BLAST hit	Not supported by synteny	Confirmed by Treefam	Different Phenotype
WBGene00021468	*epg-2*	CBG03878	1	19.47712125	Possible alternative BLAST hit	Supported by synteny	Not confirmed by Treefam	Different Phenotype
WBGene00003044	*lir-1*	CBG13028	1	10.82390874	Possible alternative BLAST hit	Supported by synteny	Not confirmed by Treefam	Different Phenotype
WBGene00001974	*hmg-4*	CBG09136	1	0	Possible alternative BLAST hit	Supported by synteny	Not confirmed by Treefam	Different Phenotype
WBGene00006428	*tag-49*	CBG19991	1	0	Possible alternative BLAST hit	Not supported by synteny	Confirmed by Treefam	Different Phenotype
WBGene00016721	*C46G7.1*	CBG09471	1	0	Possible alternative BLAST hit	Supported by synteny	Not confirmed by Treefam	Different Phenotype
WBGene00003210	*mel-28*	CBG17984	1	0	Possible alternative BLAST hit	Not supported by synteny	Confirmed by Treefam	Different Phenotype
WBGene00004418	*rpl-7*	CBG03905	3	175.0791812	Unambiguous reciprocal BLAST	Supported by synteny	Confirmed by Treefam	
WBGene00002077	*imb-3*	CBG22062	3	Inf	Unambiguous reciprocal BLAST	Supported by synteny	Confirmed by Treefam	
WBGene00020915	*nol-5*	CBG14983	3	Inf	Unambiguous reciprocal BLAST	Supported by synteny	Confirmed by Treefam	
WBGene00004077	*pop-1*	CBG04236	3	116.9542425	Unambiguous reciprocal BLAST	Not supported by synteny	Confirmed by Treefam	
WBGene00021270	*Y23H5A.3*	CBG14989	3	177.8624721	Unambiguous reciprocal BLAST	Supported by synteny	Confirmed by Treefam	
WBGene00017735	*F23C8.6*	CBG03984	3	131.39794	Unambiguous reciprocal BLAST	Supported by synteny	Confirmed by Treefam	
WBGene00019698	*riok-1*	CBG04203	3	Inf	Unambiguous reciprocal BLAST	Supported by synteny	Confirmed by Treefam	
WBGene00003062	*lpd-6*	CBG04177	3	Inf	Unambiguous reciprocal BLAST	Not supported by synteny	Not confirmed by Treefam	
WBGene00020190	*T03F1.8*	CBG11972	3	119.39794	Unambiguous reciprocal BLAST	Supported by synteny	Confirmed by Treefam	
WBGene00019362	*cdk-2*	CBG14952	3	Inf	Unambiguous reciprocal BLAST	Supported by synteny	Confirmed by Treefam	
WBGene00016837	*C50F2.3*	CBG11979	3	Inf	Unambiguous reciprocal BLAST	Supported by synteny	Confirmed by Treefam	
WBGene00001832	*hcp-4*	CBG11973	3	60.23044892	Unambiguous reciprocal BLAST	Supported by synteny	Not confirmed by Treefam	
WBGene00003797	*npp-11*	CBG12010	3	169.154902	Unambiguous reciprocal BLAST	Supported by synteny	Confirmed by Treefam	
WBGene00004425	*rpl-13*	CBG12003	3	142.243038	Unambiguous reciprocal BLAST	Supported by synteny	Confirmed by Treefam	
WBGene00003950	*pbs-4*	CBG12037	3	131	Unambiguous reciprocal BLAST	Supported by synteny	Confirmed by Treefam	
WBGene00001231	*eif-3.H*	CBG04003	3	Inf	Unambiguous reciprocal BLAST	Supported by synteny	Confirmed by Treefam	
WBGene00007001	*tufm-2*	CBG12179	3	Inf	Unambiguous reciprocal BLAST	Supported by synteny	Confirmed by Treefam	
WBGene00017086	*E01A2.4*	CBG12065	3	136.1760913	Unambiguous reciprocal BLAST	Supported by synteny	Confirmed by Treefam	
WBGene00003061	*lpd-5*	CBG23930	3	119.5563025	Unambiguous reciprocal BLAST	Supported by synteny	Confirmed by Treefam	
WBGene00000779	*cpn-3*	CBG12084	3	51.52287875	Unambiguous reciprocal BLAST	Supported by synteny	Confirmed by Treefam	
WBGene00000140	*anc-1*	CBG12190	3	Inf	Unambiguous reciprocal BLAST	Supported by synteny	Confirmed by Treefam	
WBGene00004464	*rpn-8*	CBG12090	3	Inf	Unambiguous reciprocal BLAST	Supported by synteny	Not confirmed by Treefam	
WBGene00016607	*C43E11.9*	CBG12182	3	131.6734159	Unambiguous reciprocal BLAST	Not supported by synteny	Confirmed by Treefam	
WBGene00002694	*let-502*	CBG03960	3	Inf	Unambiguous reciprocal BLAST	Supported by synteny	Confirmed by Treefam	
WBGene00004436	*rpl-24.1*	CBG22273	3	81	Unambiguous reciprocal BLAST	Supported by synteny	Confirmed by Treefam	
WBGene00004415	*rpl-4*	CBG22235	3	Inf	Unambiguous reciprocal BLAST	Supported by synteny	Confirmed by Treefam	
WBGene00004479	*rps-10*	CBG22274	3	100.8293038	Unambiguous reciprocal BLAST	Supported by synteny	Confirmed by Treefam	
WBGene00018797	*F54D7.2*	CBG03950	3	Inf	Unambiguous reciprocal BLAST	Supported by synteny	Confirmed by Treefam	

WBGene00018909	*slx-1*	CBG23741	3	129.3891661	Unambiguous reciprocal BLAST	Supported by synteny	Confirmed by Treefam	
WBGene00003221	*mes-3*	CBG10773	3	31.47712125	Unambiguous reciprocal BLAST	Supported by synteny	Confirmed by Treefam	
WBGene00000502	*chp-1*	CBG10812	3	Inf	Unambiguous reciprocal BLAST	Supported by synteny	Confirmed by Treefam	
WBGene00015091	*B0261.1*	CBG12854	3	Inf	Unambiguous reciprocal BLAST	Supported by synteny	Confirmed by Treefam	
WBGene00003132	*mat-1*	CBG10827	3	Inf	Unambiguous reciprocal BLAST	Supported by synteny	Confirmed by Treefam	
WBGene00022460	*use-1*	CBG10814	3	110.90309	Unambiguous reciprocal BLAST	Supported by synteny	Confirmed by Treefam	
WBGene00018866	*F55A12.8*	CBG12327	3	Inf	Unambiguous reciprocal BLAST	Supported by synteny	Confirmed by Treefam	
WBGene00017855	*F27C1.6*	CBG12310	3	Inf	Unambiguous reciprocal BLAST	Supported by synteny	Confirmed by Treefam	
WBGene00022464	*Y110A7A.19*	CBG23745	3	Inf	Unambiguous reciprocal BLAST	Not supported by synteny	Confirmed by Treefam	
WBGene00004955	*spd-5*	CBG23743	3	Inf	Unambiguous reciprocal BLAST	Supported by synteny	Confirmed by Treefam	
WBGene00022458	*Y110A7A.8*	CBG10828	3	Inf	Unambiguous reciprocal BLAST	Supported by synteny	Confirmed by Treefam	
WBGene00020263	*T05E8.3*	CBG12480	3	Inf	Unambiguous reciprocal BLAST	Not supported by synteny	Confirmed by Treefam	
WBGene00015175	*srz-4*	CBG04062	3	57.60205999	Unambiguous reciprocal BLAST	Supported by synteny	Not confirmed by Treefam	
WBGene00003793	*npp-7*	CBG14918	3	Inf	Unambiguous reciprocal BLAST	Supported by synteny	Confirmed by Treefam	
WBGene00019076	*F59A3.3*	CBG12482	3	Inf	Unambiguous reciprocal BLAST	Supported by synteny	Confirmed by Treefam	
WBGene00004505	*rpt-5*	CBG12876	3	Inf	Unambiguous reciprocal BLAST	Supported by synteny	Confirmed by Treefam	
WBGene00019693	*ostd-1*	CBG14887	3	159.6283889	Unambiguous reciprocal BLAST	Supported by synteny	Confirmed by Treefam	
WBGene00006919	*vha-10*	CBG14904	3	82.77815125	Unambiguous reciprocal BLAST	Supported by synteny	Confirmed by Treefam	
WBGene00020558	*T19B4.5*	CBG14914	3	179.5185139	Unambiguous reciprocal BLAST	Supported by synteny	Confirmed by Treefam	
WBGene00004431	*rpl-19*	CBG12479	3	138.2130748	Unambiguous reciprocal BLAST	Supported by synteny	Confirmed by Treefam	
WBGene00018891	*F55F8.3*	CBG14923	3	Inf	Unambiguous reciprocal BLAST	Supported by synteny	Confirmed by Treefam	
WBGene00020383	*T09B4.9*	CBG12550	3	Inf	Unambiguous reciprocal BLAST	Supported by synteny	Confirmed by Treefam	
WBGene00020348	*T08B2.8*	CBG12563	3	104.559308	Unambiguous reciprocal BLAST	Supported by synteny	Confirmed by Treefam	
WBGene00015499	*C06A5.1*	CBG22096	3	Inf	Unambiguous reciprocal BLAST	Supported by synteny	Confirmed by Treefam	
WBGene00003059	*lpd-2*	CBG12576	3	169.2086205	Unambiguous reciprocal BLAST	Supported by synteny	Confirmed by Treefam	
WBGene00003878	*pept-3*	CBG20288	3	Inf	Unambiguous reciprocal BLAST	Supported by synteny	Confirmed by Treefam	
WBGene00004486	*rps-17*	CBG12566	3	89.09691001	Unambiguous reciprocal BLAST	Supported by synteny	Confirmed by Treefam	
WBGene00019005	*F57B10.8*	CBG12662	3	125.1139434	Unambiguous reciprocal BLAST	Not supported by synteny	Confirmed by Treefam	
WBGene00020417	*nuo-2*	CBG12670	3	Inf	Unambiguous reciprocal BLAST	Not supported by synteny	Confirmed by Treefam	
WBGene00018156	*ncbp-1*	CBG12621	3	Inf	Unambiguous reciprocal BLAST	Supported by synteny	Confirmed by Treefam	
WBGene00016944	*uri-1*	CBG04072	3	175.69897	Unambiguous reciprocal BLAST	Not supported by synteny	Confirmed by Treefam	
WBGene00022591	*cuti-1*	CBG12615	3	109.5882717	Unambiguous reciprocal BLAST	Supported by synteny	Confirmed by Treefam	
WBGene00019432	*knl-2*	CBG12632	3	Inf	Unambiguous reciprocal BLAST	Supported by synteny	Not confirmed by Treefam	
WBGene00006773	*unc-37*	CBG04050	3	Inf	Unambiguous reciprocal BLAST	Supported by synteny	Confirmed by Treefam	
WBGene00002257	*lbp-5*	CBG12714	3	23.24303805	Unambiguous reciprocal BLAST	Supported by synteny	Confirmed by Treefam	
WBGene00016740	*C48B6.2*	CBG12733	3	118.1249387	Unambiguous reciprocal BLAST	Supported by synteny	Confirmed by Treefam	
WBGene00003815	*nars-1*	CBG20478	3	Inf	Unambiguous reciprocal BLAST	Supported by synteny	Confirmed by Treefam	
WBGene00019322	*ahcy-1*	CBG12756	3	Inf	Unambiguous reciprocal BLAST	Supported by synteny	Confirmed by Treefam	
WBGene00009051	*nduf-6*	CBG20477	3	97.7201593	Unambiguous reciprocal BLAST	Supported by synteny	Confirmed by Treefam	
WBGene00012166	*nuo-6*	CBG10781	3	125.6127839	Unambiguous reciprocal BLAST	Supported by synteny	Confirmed by Treefam	
WBGene00009052	*ekl-1*	CBG20475	3	Inf	Unambiguous reciprocal BLAST	Supported by synteny	Confirmed by Treefam	
WBGene00001648	*goa-1*	CBG24698	3	Inf	Unambiguous reciprocal BLAST	Not supported by synteny	Confirmed by Treefam	
WBGene00001949	*hlh-2*	CBG21941	3	101.4771213	Unambiguous reciprocal BLAST	Supported by synteny	Confirmed by Treefam	
WBGene00007042	*pbrm-1*	CBG11867	3	Inf	Unambiguous reciprocal BLAST	Supported by synteny	Confirmed by Treefam	
WBGene00009004	*pfd-6*	CBG21902	3	64.54406804	Unambiguous reciprocal BLAST	Supported by synteny	Confirmed by Treefam	
WBGene00009915	*F52A8.1*	CBG11905	3	54.82390874	Unambiguous reciprocal BLAST	Supported by synteny	Confirmed by Treefam	
WBGene00009254	*capg-1*	CBG11864	3	Inf	Unambiguous reciprocal BLAST	Not supported by synteny	Confirmed by Treefam	
WBGene00008990	*smgl-1*	CBG08249	3	Inf	Unambiguous reciprocal BLAST	Supported by synteny	Confirmed by Treefam	
WBGene00010560	*iftb-1*	CBG08214	3	132.69897	Unambiguous reciprocal BLAST	Supported by synteny	Confirmed by Treefam	
WBGene00010562	*cdc-48.3*	CBG08216	3	Inf	Unambiguous reciprocal BLAST	Supported by synteny	Confirmed by Treefam	
WBGene00009141	*ncbp-2*	CBG22194	3	49.04575749	Unambiguous reciprocal BLAST	Supported by synteny	Confirmed by Treefam	
WBGene00012030	*T25G3.3*	CBG11892	3	Inf	Unambiguous reciprocal BLAST	Supported by synteny	Confirmed by Treefam	
WBGene00001595	*gld-1*	CBG00303	3	Inf	Unambiguous reciprocal BLAST	Supported by synteny	Confirmed by Treefam	
WBGene00006386	*taf-5*	CBG23733	3	Inf	Unambiguous reciprocal BLAST	Supported by synteny	Confirmed by Treefam	
WBGene00008413	*D2030.3*	CBG11961	3	Inf	Unambiguous reciprocal BLAST	Not supported by synteny	Confirmed by Treefam	
WBGene00001647	*gna-2*	CBG19292	3	Inf	Unambiguous reciprocal BLAST	Supported by synteny	Confirmed by Treefam	
WBGene00008414	*D2030.4*	CBG11960	3	86.94816836	Unambiguous reciprocal BLAST	Supported by synteny	Confirmed by Treefam	
WBGene00000869	*cyc-1*	CBG08212	3	Inf	Unambiguous reciprocal BLAST	Supported by synteny	Confirmed by Treefam	
WBGene00004439	*rpl-25.2*	CBG04080	3	22	Unambiguous reciprocal BLAST	Supported by synteny	Confirmed by Treefam	
WBGene00003788	*npp-2*	CBG04087	3	Inf	Unambiguous reciprocal BLAST	Supported by synteny	Confirmed by Treefam	

WBGene00006844	*unc-120*	CBG12542	3	167.60206	Unambiguous reciprocal BLAST	Supported by synteny	Confirmed by Treefam		
WBGene00002196	*kin-10*	CBG04088	3	157.1326256	Unambiguous reciprocal BLAST	Supported by synteny	Confirmed by Treefam		
WBGene00008386	*D1081.8*	CBG12532	3	Inf	Unambiguous reciprocal BLAST	Supported by synteny	Confirmed by Treefam		
WBGene00013958	*ZK265.6*	CBG24963	3	103.4259687	Unambiguous reciprocal BLAST	Supported by synteny	Confirmed by Treefam		
WBGene00010496	*sec-12*	CBG12530	3	Inf	Unambiguous reciprocal BLAST	Supported by synteny	Confirmed by Treefam		
WBGene00012116	*del-4*	CBG00319	3	Inf	Unambiguous reciprocal BLAST	Not supported by synteny	Not confirmed by Treefam		
WBGene00003925	*pas-4*	CBG12349	3	127.845098	Unambiguous reciprocal BLAST	Supported by synteny	Confirmed by Treefam		
WBGene00007972	*prp-4*	CBG12355	3	Inf	Unambiguous reciprocal BLAST	Supported by synteny	Confirmed by Treefam		
WBGene00009671	*mfap-1*	CBG12497	3	Inf	Unambiguous reciprocal BLAST	Supported by synteny	Confirmed by Treefam		
WBGene00009454	*F36A2.7*	CBG12375	3	30.6180481	Unambiguous reciprocal BLAST	Supported by synteny	Not confirmed by Treefam		
WBGene00009342	*fasn-1*	CBG12408	3	Inf	Unambiguous reciprocal BLAST	Supported by synteny	Confirmed by Treefam		
WBGene00003052	*lmn-1*	CBG12363	3	Inf	Unambiguous reciprocal BLAST	Supported by synteny	Confirmed by Treefam		
WBGene00009664	*F43G9.1*	CBG12504	3	Inf	Unambiguous reciprocal BLAST	Supported by synteny	Confirmed by Treefam		
WBGene00009672	*F43G9.12*	CBG12496	3	Inf	Unambiguous reciprocal BLAST	Supported by synteny	Confirmed by Treefam		
WBGene00007971	*rpb-3*	CBG12350	3	Inf	Unambiguous reciprocal BLAST	Supported by synteny	Confirmed by Treefam		
WBGene00004953	*spd-2*	CBG12404	3	Inf	Unambiguous reciprocal BLAST	Supported by synteny	Not confirmed by Treefam		
WBGene00004484	*rps-15*	CBG12376	3	105.544068	Unambiguous reciprocal BLAST	Supported by synteny	Confirmed by Treefam		
WBGene00003953	*pbs-7*	CBG12343	3	123.30103	Unambiguous reciprocal BLAST	Supported by synteny	Confirmed by Treefam		
WBGene00008781	*F14B4.3*	CBG19469	3	Inf	Unambiguous reciprocal BLAST	Supported by synteny	Confirmed by Treefam		
WBGene00007646	*nkb-1*	CBG03745	3	Inf	Unambiguous reciprocal BLAST	Supported by synteny	Confirmed by Treefam		
WBGene00009266	*F30A10.9*	CBG24495	3	123.7781513	Unambiguous reciprocal BLAST	Supported by synteny	Confirmed by Treefam		
WBGene00009477	*tag-214*	CBG12417	3	Inf	Unambiguous reciprocal BLAST	Supported by synteny	Confirmed by Treefam		
WBGene00008061	*C41G7.3*	CBG24491	3	45.17609126	Unambiguous reciprocal BLAST	Supported by synteny	Not confirmed by Treefam		
WBGene00008107	*aspm-1*	CBG09326	3	Inf	Unambiguous reciprocal BLAST	Not supported by synteny	Confirmed by Treefam		
WBGene00000371	*cco-1*	CBG03796	3	93.78612018	Unambiguous reciprocal BLAST	Supported by synteny	Confirmed by Treefam		
WBGene00006352	*sur-6*	CBG03807	3	Inf	Unambiguous reciprocal BLAST	Supported by synteny	Confirmed by Treefam		
WBGene00004426	*rpl-14*	CBG03771	3	83.38021124	Unambiguous reciprocal BLAST	Supported by synteny	Confirmed by Treefam		
WBGene00004312	*rba-1*	CBG03711	3	Inf	Unambiguous reciprocal BLAST	Supported by synteny	Confirmed by Treefam		
WBGene00003036	*lin-53*	CBG03710	3	Inf	Unambiguous reciprocal BLAST	Supported by synteny	Confirmed by Treefam		
WBGene00009159	*F26E4.4*	CBG03802	3	158.8846066	Unambiguous reciprocal BLAST	Supported by synteny	Not confirmed by Treefam		
WBGene00004437	*rpl-24.2*	CBG03702	3	103	Unambiguous reciprocal BLAST	Supported by synteny	Confirmed by Treefam		
WBGene00006379	*sys-1*	CBG03820	3	Inf	Unambiguous reciprocal BLAST	Supported by synteny	Not confirmed by Treefam		
WBGene00003000	*lin-11*	CBG12236	3	Inf	Unambiguous reciprocal BLAST	Not supported by synteny	Confirmed by Treefam		
WBGene00001226	*eif-3.C*	CBG03821	3	Inf	Unambiguous reciprocal BLAST	Supported by synteny	Confirmed by Treefam		
WBGene00004476	*rps-7*	CBG21343	3	136.4819201	Unambiguous reciprocal BLAST	Supported by synteny	Confirmed by Treefam		
WBGene00000843	*cup-2*	CBG02198	3	155.39794	Unambiguous reciprocal BLAST	Supported by synteny	Confirmed by Treefam		
WBGene00003397	*mom-5*	CBG03824	3	Inf	Unambiguous reciprocal BLAST	Supported by synteny	Confirmed by Treefam		
WBGene00011944	*T23D8.3*	CBG03822	3	Inf	Unambiguous reciprocal BLAST	Supported by synteny	Confirmed by Treefam		
WBGene00003803	*npp-17*	CBG03784	3	Inf	Unambiguous reciprocal BLAST	Supported by synteny	Confirmed by Treefam		
WBGene00016393	*C34B2.8*	CBG12806	3	116.071882	Unambiguous reciprocal BLAST	Supported by synteny	Confirmed by Treefam		
WBGene00009118	*F25H2.4*	CBG02246	3	108.39794	Unambiguous reciprocal BLAST	Not supported by synteny	Confirmed by Treefam		
WBGene00009122	*tct-1*	CBG02259	3	126.4771213	Unambiguous reciprocal BLAST	Supported by synteny	Confirmed by Treefam		
WBGene00003926	*pas-5*	CBG02261	3	134.154902	Unambiguous reciprocal BLAST	Supported by synteny	Confirmed by Treefam		
WBGene00044321	*tag-264*	CBG12459	3	Inf	Unambiguous reciprocal BLAST	Supported by synteny	Confirmed by Treefam		
WBGene00010326	*F59C6.5*	CBG02223	3	Inf	Unambiguous reciprocal BLAST	Supported by synteny	Confirmed by Treefam		
WBGene00018849	*F55A3.3*	CBG12204	3	Inf	Unambiguous reciprocal BLAST	Supported by synteny	Not confirmed by Treefam		
WBGene00013128	*Y52B11A.9*	CBG23783	3	Inf	Unambiguous reciprocal BLAST	Supported by synteny	Confirmed by Treefam		
WBGene00013605	*Y95D11A.1*	CBG20410	3	66.30103	Unambiguous reciprocal BLAST	Supported by synteny	Confirmed by Treefam		
WBGene00018625	*prp-17*	CBG20408	3	Inf	Unambiguous reciprocal BLAST	Supported by synteny	Confirmed by Treefam		
WBGene00012950	*Y47H9C.7*	CBG15840	3	Inf	Unambiguous reciprocal BLAST	Not supported by synteny	Confirmed by Treefam		
WBGene00006707	*ubc-12*	CBG18691	3	25.69897	Unambiguous reciprocal BLAST	Supported by synteny	Confirmed by Treefam		
WBGene00007603	*C15C6.4*	CBG23842	3	111.3891661	Unambiguous reciprocal BLAST	Supported by synteny	Confirmed by Treefam		
WBGene00003921	*par-6*	CBG08713	3	Inf	Unambiguous reciprocal BLAST	Supported by synteny	Confirmed by Treefam		
WBGene00006889	*pfd-3*	CBG13600	3	113.4983106	Unambiguous reciprocal BLAST	Supported by synteny	Confirmed by Treefam		
WBGene00012484	*car-1*	CBG07987	3	Inf	Unambiguous reciprocal BLAST	Supported by synteny	Confirmed by Treefam		
WBGene00003989	*pfn-1*	CBG04366	3	26.14612804	Unambiguous reciprocal BLAST	Supported by synteny	Confirmed by Treefam		
WBGene00003948	*pbs-2*	CBG07938	3	Inf	Unambiguous reciprocal BLAST	Supported by synteny	Confirmed by Treefam		
WBGene00006700	*uba-2*	CBG13604	3	Inf	Unambiguous reciprocal BLAST	Supported by synteny	Confirmed by Treefam		
WBGene00001746	*gsk-3*	CBG07972	3	Inf	Unambiguous reciprocal BLAST	Supported by synteny	Confirmed by Treefam		
WBGene00007215	*C01A2.3*	CBG20396	3	Inf	Unambiguous reciprocal BLAST	Supported by synteny	Confirmed by Treefam		

WBGene00012735	*sptf-3*	CBG20386	3	70.69897	Unambiguous reciprocal BLAST	Not supported by synteny	Confirmed by Treefam	
WBGene00013021	*Y48G10A.4*	CBG08018	3	Inf	Unambiguous reciprocal BLAST	Supported by synteny	Not confirmed by Treefam	
WBGene00004915	*snr-2*	CBG20368	3	67.57403127	Unambiguous reciprocal BLAST	Supported by synteny	Confirmed by Treefam	
WBGene00006595	*top-1*	CBG20349	3	Inf	Unambiguous reciprocal BLAST	Supported by synteny	Confirmed by Treefam	
WBGene00006936	*vars-2*	CBG08035	3	Inf	Unambiguous reciprocal BLAST	Supported by synteny	Confirmed by Treefam	
WBGene00013598	*vps-28*	CBG19782	3	141.7269987	Unambiguous reciprocal BLAST	Supported by synteny	Confirmed by Treefam	
WBGene00007217	*C01A2.5*	CBG20397	3	161.30103	Unambiguous reciprocal BLAST	Not supported by synteny	Confirmed by Treefam	
WBGene00004489	*rps-20*	CBG08754	3	79.50514998	Unambiguous reciprocal BLAST	Supported by synteny	Confirmed by Treefam	
WBGene00006382	*taf-1*	CBG13620	3	Inf	Unambiguous reciprocal BLAST	Supported by synteny	Confirmed by Treefam	
WBGene00009825	*F47G4.4*	CBG18869	3	Inf	Unambiguous reciprocal BLAST	Supported by synteny	Confirmed by Treefam	
WBGene00006565	*tfg-1*	CBG18822	3	125.60206	Unambiguous reciprocal BLAST	Supported by synteny	Confirmed by Treefam	
WBGene00013406	*Y63D3A.7*	CBG18827	3	55.09691001	Unambiguous reciprocal BLAST	Supported by synteny	Confirmed by Treefam	
WBGene00009368	*F33H2.5*	CBG19758	3	Inf	Unambiguous reciprocal BLAST	Supported by synteny	Confirmed by Treefam	
WBGene00000158	*apg-1*	CBG08748	3	Inf	Unambiguous reciprocal BLAST	Supported by synteny	Confirmed by Treefam	
WBGene00002324	*let-49*	CBG19704	3	117.3424227	Unambiguous reciprocal BLAST	Supported by synteny	Confirmed by Treefam	
WBGene00010766	*K11B4.1*	CBG19712	3	Inf	Unambiguous reciprocal BLAST	Supported by synteny	Confirmed by Treefam	
WBGene00009287	*F31C3.5*	CBG19768	3	102.1398791	Unambiguous reciprocal BLAST	Not supported by synteny	Confirmed by Treefam	
WBGene00009369	*F33H2.6*	CBG19724	3	149.0791812	Unambiguous reciprocal BLAST	Supported by synteny	Confirmed by Treefam	
WBGene00004495	*rps-26*	CBG19764	3	69.11394335	Unambiguous reciprocal BLAST	Supported by synteny	Confirmed by Treefam	
WBGene00000088	*aex-5*	CBG19714	3	Inf	Unambiguous reciprocal BLAST	Supported by synteny	Confirmed by Treefam	
WBGene00001030	*dnj-12*	CBG19766	3	Inf	Unambiguous reciprocal BLAST	Supported by synteny	Confirmed by Treefam	
WBGene00003951	*pbs-5*	CBG19741	3	Inf	Unambiguous reciprocal BLAST	Supported by synteny	Confirmed by Treefam	
WBGene00010579	*K05C4.2*	CBG19742	3	130.4548449	Unambiguous reciprocal BLAST	Supported by synteny	Confirmed by Treefam	
WBGene00002047	*icp-1*	CBG03880	3	144.69897	Unambiguous reciprocal BLAST	Supported by synteny	Confirmed by Treefam	
WBGene00021334	*vps-4*	CBG22083	3	Inf	Unambiguous reciprocal BLAST	Supported by synteny	Confirmed by Treefam	
WBGene00021466	*Y39G10AR.8*	CBG03884	3	Inf	Unambiguous reciprocal BLAST	Supported by synteny	Confirmed by Treefam	
WBGene00003156	*mcm-4*	CBG03890	3	Inf	Unambiguous reciprocal BLAST	Not supported by synteny	Confirmed by Treefam	
WBGene00004978	*spg-7*	CBG14947	3	Inf	Unambiguous reciprocal BLAST	Not supported by synteny	Confirmed by Treefam	
WBGene00022025	*Y65B4A.1*	CBG18539	3	24.30103	Unambiguous reciprocal BLAST	Supported by synteny	Not confirmed by Treefam	
WBGene00021638	*Y47G6A.9*	CBG14946	3	88.68124124	Unambiguous reciprocal BLAST	Supported by synteny	Confirmed by Treefam	
WBGene00021843	*Y54E10BR.4*	CBG04192	3	Inf	Unambiguous reciprocal BLAST	Supported by synteny	Confirmed by Treefam	
WBGene00021845	*rpb-7*	CBG04195	3	117.9330532	Unambiguous reciprocal BLAST	Supported by synteny	Confirmed by Treefam	
WBGene00000794	*crn-1*	CBG14945	3	Inf	Unambiguous reciprocal BLAST	Supported by synteny	Confirmed by Treefam	
WBGene00000413	*cdt-1*	CBG14978	3	Inf	Unambiguous reciprocal BLAST	Supported by synteny	Confirmed by Treefam	
WBGene00021829	*Y54E10A.7*	CBG04173	3	121.3117539	Unambiguous reciprocal BLAST	Supported by synteny	Confirmed by Treefam	
WBGene00002079	*xpo-2*	CBG22063	3	Inf	Unambiguous reciprocal BLAST	Supported by synteny	Confirmed by Treefam	
WBGene00004920	*snr-7*	CBG04114	3	31	Unambiguous reciprocal BLAST	Supported by synteny	Confirmed by Treefam	
WBGene00003080	*lsm-6*	CBG22150	3	34.87506126	Unambiguous reciprocal BLAST	Supported by synteny	Confirmed by Treefam	
WBGene00022119	*Y71F9AL.17*	CBG04223	3	Inf	Unambiguous reciprocal BLAST	Not supported by synteny	Confirmed by Treefam	
WBGene00017746	*F23F1.5*	CBG07011	3	Inf	Unambiguous reciprocal BLAST	Supported by synteny	Confirmed by Treefam	
WBGene00000497	*chs-2*	CBG06966	3	Inf	Unambiguous reciprocal BLAST	Supported by synteny	Confirmed by Treefam	
WBGene00020796	*T25D3.2*	CBG06974	3	Inf	Unambiguous reciprocal BLAST	Supported by synteny	Confirmed by Treefam	
WBGene00015185	*mrpl-41*	CBG07017	3	111.7781513	Unambiguous reciprocal BLAST	Supported by synteny	Confirmed by Treefam	
WBGene00021073	*nol-1*	CBG07065	3	Inf	Unambiguous reciprocal BLAST	Supported by synteny	Confirmed by Treefam	
WBGene00004188	*prp-21*	CBG07063	3	Inf	Unambiguous reciprocal BLAST	Supported by synteny	Confirmed by Treefam	
WBGene00021095	*mlt-8*	CBG07073	3	Inf	Unambiguous reciprocal BLAST	Supported by synteny	Not confirmed by Treefam	
WBGene00021133	*tomm-22*	CBG07066	3	64.77815125	Unambiguous reciprocal BLAST	Supported by synteny	Confirmed by Treefam	
WBGene00021074	*W07E6.2*	CBG07064	3	Inf	Unambiguous reciprocal BLAST	Supported by synteny	Confirmed by Treefam	
WBGene00017210	*F07E5.5*	CBG20443	3	173	Unambiguous reciprocal BLAST	Not supported by synteny	Confirmed by Treefam	
WBGene00017280	*F09D1.1*	CBG04341	3	Inf	Unambiguous reciprocal BLAST	Not supported by synteny	Confirmed by Treefam	
WBGene00017245	*F08D12.1*	CBG19592	3	Inf	Unambiguous reciprocal BLAST	Supported by synteny	Confirmed by Treefam	
WBGene00021292	*Y25C1A.5*	CBG19635	3	Inf	Unambiguous reciprocal BLAST	Supported by synteny	Confirmed by Treefam	
WBGene00021715	*Y49F6B.2*	CBG17763	3	134.2552725	Unambiguous reciprocal BLAST	Supported by synteny	Confirmed by Treefam	
WBGene00004467	*rpn-11*	CBG23950	3	Inf	Unambiguous reciprocal BLAST	Supported by synteny	Confirmed by Treefam	
WBGene00019220	*pfd-2*	CBG03668	3	71.75587486	Unambiguous reciprocal BLAST	Supported by synteny	Confirmed by Treefam	
WBGene00022739	*toe-1*	CBG03684	3	Inf	Unambiguous reciprocal BLAST	Supported by synteny	Confirmed by Treefam	
WBGene00015297	*sco-1*	CBG03655	3	Inf	Unambiguous reciprocal BLAST	Supported by synteny	Confirmed by Treefam	
WBGene00006497	*tag-151*	CBG04291	3	Inf	Unambiguous reciprocal BLAST	Supported by synteny	Confirmed by Treefam	
WBGene00001438	*fkh-6*	CBG03678	3	142.9420081	Unambiguous reciprocal BLAST	Supported by synteny	Confirmed by Treefam	
WBGene00022742	*ZK430.7*	CBG03683	3	Inf	Unambiguous reciprocal BLAST	Supported by synteny	Confirmed by Treefam	

WBGene00018016	*lrr-1*	CBG02302	3	Inf	Unambiguous reciprocal BLAST	Supported by synteny	Confirmed by Treefam	
WBGene00020866	*T27F7.1*	CBG02295	3	118.30103	Unambiguous reciprocal BLAST	Supported by synteny	Confirmed by Treefam	
WBGene00016163	*C27D9.1*	CBG20621	3	Inf	Unambiguous reciprocal BLAST	Not supported by synteny	Confirmed by Treefam	
WBGene00003949	*pbs-3*	CBG02289	3	139.4771213	Unambiguous reciprocal BLAST	Supported by synteny	Confirmed by Treefam	
WBGene00004434	*rpl-22*	CBG02309	3	65.47712125	Unambiguous reciprocal BLAST	Supported by synteny	Confirmed by Treefam	
WBGene00007012	*mdt-4*	CBG02282	3	165.3802112	Unambiguous reciprocal BLAST	Not supported by synteny	Not confirmed by Treefam	
WBGene00018270	*F41C3.4*	CBG20627	3	32.07918125	Unambiguous reciprocal BLAST	Not supported by synteny	Not confirmed by Treefam	
WBGene00002068	*ify-1*	CBG02465	3	75.96108193	Unambiguous reciprocal BLAST	Not supported by synteny	Confirmed by Treefam	
WBGene00017289	*F09E5.11*	CBG02376	3	139.7403627	Unambiguous reciprocal BLAST	Supported by synteny	Confirmed by Treefam	
WBGene00001511	*fzy-1*	CBG02417	3	Inf	Unambiguous reciprocal BLAST	Supported by synteny	Confirmed by Treefam	
WBGene00017282	*F09E5.2*	CBG02380	3	Inf	Unambiguous reciprocal BLAST	Supported by synteny	Confirmed by Treefam	
WBGene00000969	*dhs-5*	CBG02403	3	Inf	Unambiguous reciprocal BLAST	Supported by synteny	Confirmed by Treefam	
WBGene00004034	*pkc-3*	CBG02381	3	Inf	Unambiguous reciprocal BLAST	Supported by synteny	Confirmed by Treefam	
WBGene00017132	*mel-47*	CBG02394	3	Inf	Unambiguous reciprocal BLAST	Supported by synteny	Not confirmed by Treefam	
WBGene00015941	*C18A3.3*	CBG02485	3	162.7160033	Unambiguous reciprocal BLAST	Not supported by synteny	Confirmed by Treefam	
WBGene00006933	*vps-35*	CBG02534	3	Inf	Unambiguous reciprocal BLAST	Not supported by synteny	Confirmed by Treefam	
WBGene00006988	*zyg-1*	CBG02462	3	Inf	Unambiguous reciprocal BLAST	Supported by synteny	Confirmed by Treefam	
WBGene00001509	*fzo-1*	CBG02514	3	Inf	Unambiguous reciprocal BLAST	Supported by synteny	Confirmed by Treefam	
WBGene00019268	*H41C03.1*	CBG02523	3	Inf	Unambiguous reciprocal BLAST	Supported by synteny	Confirmed by Treefam	
WBGene00016319	*C32D5.12*	CBG02636	3	Inf	Unambiguous reciprocal BLAST	Supported by synteny	Not confirmed by Treefam	
WBGene00003009	*lin-23*	CBG02640	3	Inf	Unambiguous reciprocal BLAST	Supported by synteny	Confirmed by Treefam	
WBGene00017683	*F21H12.1*	CBG02573	3	Inf	Unambiguous reciprocal BLAST	Supported by synteny	Confirmed by Treefam	
WBGene00016243	*tag-319*	CBG02591	3	Inf	Unambiguous reciprocal BLAST	Supported by synteny	Confirmed by Treefam	
WBGene00004015	*phb-2*	CBG02605	3	Inf	Unambiguous reciprocal BLAST	Supported by synteny	Confirmed by Treefam	
WBGene00016245	*C30B5.4*	CBG02589	3	Inf	Unambiguous reciprocal BLAST	Supported by synteny	Confirmed by Treefam	
WBGene00017895	*vrk-1*	CBG02540	3	Inf	Unambiguous reciprocal BLAST	Supported by synteny	Confirmed by Treefam	
WBGene00002045	*icd-1*	CBG04320	3	112.146128	Unambiguous reciprocal BLAST	Supported by synteny	Confirmed by Treefam	
WBGene00004916	*snr-3*	CBG02671	3	48.60205999	Unambiguous reciprocal BLAST	Supported by synteny	Confirmed by Treefam	
WBGene00016961	*vps-32.1*	CBG04319	3	31.69897	Unambiguous reciprocal BLAST	Supported by synteny	Not confirmed by Treefam	
WBGene00016968	*epg-5*	CBG04313	3	Inf	Unambiguous reciprocal BLAST	Supported by synteny	Confirmed by Treefam	
WBGene00020499	*T14B4.2*	CBG11219	3	76.45593196	Unambiguous reciprocal BLAST	Supported by synteny	Confirmed by Treefam	
WBGene00017348	*F10E7.6*	CBG13069	3	67.36797679	Unambiguous reciprocal BLAST	Supported by synteny	Not confirmed by Treefam	
WBGene00022852	*ZK1127.5*	CBG11211	3	Inf	Unambiguous reciprocal BLAST	Supported by synteny	Confirmed by Treefam	
WBGene00020166	*T02G5.7*	CBG24740	3	Inf	Unambiguous reciprocal BLAST	Supported by synteny	Confirmed by Treefam	
WBGene00022851	*ZK1127.4*	CBG11215	3	148.2552725	Unambiguous reciprocal BLAST	Supported by synteny	Confirmed by Treefam	
WBGene00004447	*rpl-33*	CBG13066	3	81.54406804	Unambiguous reciprocal BLAST	Supported by synteny	Confirmed by Treefam	
WBGene00000390	*cdc-42*	CBG13013	3	38.30103	Unambiguous reciprocal BLAST	Supported by synteny	Confirmed by Treefam	
WBGene00003813	*nrf-6*	CBG13350	3	Inf	Unambiguous reciprocal BLAST	Supported by synteny	Confirmed by Treefam	
WBGene00015515	*spdl-1*	CBG19326	3	173.5622929	Unambiguous reciprocal BLAST	Not supported by synteny	Not confirmed by Treefam	
WBGene00011638	*ostb-1*	CBG03302	3	Inf	Unambiguous reciprocal BLAST	Supported by synteny	Confirmed by Treefam	
WBGene00006996	*zyg-11*	CBG13353	3	Inf	Unambiguous reciprocal BLAST	Supported by synteny	Confirmed by Treefam	
WBGene00011350	*T01H3.4*	CBG03297	3	Inf	Unambiguous reciprocal BLAST	Supported by synteny	Not confirmed by Treefam	
WBGene00007433	*C08B11.3*	CBG13351	3	Inf	Unambiguous reciprocal BLAST	Supported by synteny	Confirmed by Treefam	
WBGene00007014	*mdt-10*	CBG03308	3	79.92081875	Unambiguous reciprocal BLAST	Supported by synteny	Confirmed by Treefam	
WBGene00015513	*C06A8.2*	CBG12959	3	152.8239087	Unambiguous reciprocal BLAST	Supported by synteny	Confirmed by Treefam	
WBGene00004723	*sap-49*	CBG13349	3	Inf	Unambiguous reciprocal BLAST	Supported by synteny	Confirmed by Treefam	
WBGene00017546	*rpa-1*	CBG13026	3	Inf	Unambiguous reciprocal BLAST	Supported by synteny	Confirmed by Treefam	
WBGene00011637	*T09A5.9*	CBG03304	3	111.544068	Unambiguous reciprocal BLAST	Supported by synteny	Confirmed by Treefam	
WBGene00011634	*T09A5.5*	CBG03309	3	113.3424227	Unambiguous reciprocal BLAST	Supported by synteny	Not confirmed by Treefam	
WBGene00007434	*C08B11.6*	CBG13348	3	Inf	Unambiguous reciprocal BLAST	Supported by synteny	Confirmed by Treefam	
WBGene00006913	*vha-4*	CBG03298	3	109.60206	Unambiguous reciprocal BLAST	Supported by synteny	Confirmed by Treefam	
WBGene00004264	*qua-1*	CBG00717	3	153.30103	Unambiguous reciprocal BLAST	Supported by synteny	Confirmed by Treefam	
WBGene00001498	*fars-3*	CBG00621	3	Inf	Unambiguous reciprocal BLAST	Supported by synteny	Confirmed by Treefam	
WBGene00006994	*zyg-9*	CBG00620	3	Inf	Unambiguous reciprocal BLAST	Supported by synteny	Confirmed by Treefam	
WBGene00000379	*cct-4*	CBG00674	3	Inf	Unambiguous reciprocal BLAST	Supported by synteny	Confirmed by Treefam	
WBGene00010948	*M195.2*	CBG00704	3	163.69897	Unambiguous reciprocal BLAST	Supported by synteny	Confirmed by Treefam	
WBGene00008640	*F10B5.3*	CBG00742	3	Inf	Unambiguous reciprocal BLAST	Supported by synteny	Confirmed by Treefam	
WBGene00011759	*T13H5.5*	CBG04747	3	173	Unambiguous reciprocal BLAST	Supported by synteny	Confirmed by Treefam	
WBGene00007744	*C26D10.3*	CBG00693	3	Inf	Unambiguous reciprocal BLAST	Supported by synteny	Confirmed by Treefam	
WBGene00004304	*ran-3*	CBG00692	3	Inf	Unambiguous reciprocal BLAST	Not supported by synteny	Confirmed by Treefam	

WBGene00000377	*cct-1*	CBG00721	3	Inf	Unambiguous reciprocal BLAST	Supported by synteny	Confirmed by Treefam	
WBGene00011758	*T13H5.4*	CBG04748	3	Inf	Unambiguous reciprocal BLAST	Supported by synteny	Confirmed by Treefam	
WBGene00003821	*nst-1*	CBG00673	3	Inf	Unambiguous reciprocal BLAST	Supported by synteny	Confirmed by Treefam	
WBGene00006739	*ulp-4*	CBG00748	3	Inf	Unambiguous reciprocal BLAST	Supported by synteny	Confirmed by Treefam	
WBGene00001281	*emb-27*	CBG00731	3	Inf	Unambiguous reciprocal BLAST	Supported by synteny	Confirmed by Treefam	
WBGene00010044	*F54C9.9*	CBG00558	3	Inf	Unambiguous reciprocal BLAST	Supported by synteny	Confirmed by Treefam	
WBGene00009207	*F28C6.8*	CBG00545	3	85.47712125	Unambiguous reciprocal BLAST	Supported by synteny	Confirmed by Treefam	
WBGene00006059	*stc-1*	CBG00564	3	Inf	Unambiguous reciprocal BLAST	Supported by synteny	Confirmed by Treefam	
WBGene00000187	*arl-1*	CBG00557	3	54.30103	Unambiguous reciprocal BLAST	Supported by synteny	Confirmed by Treefam	
WBGene00008428	*D2085.3*	CBG00512	3	Inf	Unambiguous reciprocal BLAST	Supported by synteny	Confirmed by Treefam	
WBGene00011885	*T21B10.3*	CBG03215	3	Inf	Unambiguous reciprocal BLAST	Supported by synteny	Confirmed by Treefam	
WBGene00004397	*rol-6*	CBG03154	3	Inf	Unambiguous reciprocal BLAST	Supported by synteny	Confirmed by Treefam	
WBGene00000773	*cpf-1*	CBG00548	3	Inf	Unambiguous reciprocal BLAST	Supported by synteny	Confirmed by Treefam	
WBGene00004440	*rpl-26*	CBG00547	3	27.24303805	Unambiguous reciprocal BLAST	Supported by synteny	Confirmed by Treefam	
WBGene00011883	*T21B10.1*	CBG03213	3	170.7558749	Unambiguous reciprocal BLAST	Supported by synteny	Confirmed by Treefam	
WBGene00011311	*T01B7.5*	CBG03160	3	Inf	Unambiguous reciprocal BLAST	Supported by synteny	Confirmed by Treefam	
WBGene00004416	*rpl-5*	CBG00561	3	Inf	Unambiguous reciprocal BLAST	Supported by synteny	Confirmed by Treefam	
WBGene00000615	*col-38*	CBG00562	3	77.47712125	Unambiguous reciprocal BLAST	Supported by synteny	Confirmed by Treefam	
WBGene00011768	*oac-46*	CBG03197	3	Inf	Unambiguous reciprocal BLAST	Supported by synteny	Confirmed by Treefam	
WBGene00004174	*pqn-95*	CBG00905	3	127.9208188	Unambiguous reciprocal BLAST	Supported by synteny	Confirmed by Treefam	
WBGene00000286	*cal-2*	CBG03220	3	47.65321251	Unambiguous reciprocal BLAST	Supported by synteny	Confirmed by Treefam	
WBGene00001134	*eat-3*	CBG20228	3	Inf	Unambiguous reciprocal BLAST	Supported by synteny	Confirmed by Treefam	
WBGene00006433	*sdhb-1*	CBG00872	3	Inf	Unambiguous reciprocal BLAST	Supported by synteny	Confirmed by Treefam	
WBGene00012000	*T24H10.1*	CBG03263	3	Inf	Unambiguous reciprocal BLAST	Supported by synteny	Confirmed by Treefam	
WBGene00007683	*C18E9.2*	CBG03222	3	171.0280287	Unambiguous reciprocal BLAST	Supported by synteny	Confirmed by Treefam	
WBGene00007684	*C18E9.4*	CBG03221	3	70.57403127	Unambiguous reciprocal BLAST	Supported by synteny	Confirmed by Treefam	
WBGene00001229	*eif-3.F*	CBG20226	3	116.5740313	Unambiguous reciprocal BLAST	Supported by synteny	Confirmed by Treefam	
WBGene00009711	*F44G4.1*	CBG03232	3	Inf	Unambiguous reciprocal BLAST	Supported by synteny	Confirmed by Treefam	
WBGene00007686	*tomm-40*	CBG03225	3	Inf	Unambiguous reciprocal BLAST	Supported by synteny	Confirmed by Treefam	
WBGene00009505	*F37B12.3*	CBG03250	3	134.7596678	Unambiguous reciprocal BLAST	Supported by synteny	Confirmed by Treefam	
WBGene00013143	*Y53C12B.1*	CBG01041	3	Inf	Unambiguous reciprocal BLAST	Supported by synteny	Confirmed by Treefam	
WBGene00008455	*E02H1.1*	CBG00961	3	Inf	Unambiguous reciprocal BLAST	Supported by synteny	Confirmed by Treefam	
WBGene00013144	*Y53C12B.2*	CBG01042	3	Inf	Unambiguous reciprocal BLAST	Supported by synteny	Confirmed by Treefam	
WBGene00013140	*mop-25.2*	CBG01052	3	Inf	Unambiguous reciprocal BLAST	Supported by synteny	Confirmed by Treefam	
WBGene00006940	*wee-1.3*	CBG01053	3	Inf	Unambiguous reciprocal BLAST	Supported by synteny	Confirmed by Treefam	
WBGene00002497	*let-268*	CBG13377	3	Inf	Unambiguous reciprocal BLAST	Supported by synteny	Confirmed by Treefam	
WBGene00003939	*pax-3*	CBG00861	3	111.39794	Unambiguous reciprocal BLAST	Supported by synteny	Confirmed by Treefam	
WBGene00011334	*T01E8.6*	CBG20218	3	69.44715803	Unambiguous reciprocal BLAST	Supported by synteny	Confirmed by Treefam	
WBGene00000995	*die-1*	CBG02697	3	Inf	Unambiguous reciprocal BLAST	Supported by synteny	Confirmed by Treefam	
WBGene00011273	*R53.4*	CBG01005	3	108.0413927	Unambiguous reciprocal BLAST	Supported by synteny	Confirmed by Treefam	
WBGene00001207	*egl-43*	CBG01007	3	Inf	Unambiguous reciprocal BLAST	Supported by synteny	Confirmed by Treefam	
WBGene00009364	*F33H1.3*	CBG00888	3	169.0457575	Unambiguous reciprocal BLAST	Supported by synteny	Confirmed by Treefam	
WBGene00011275	*R53.6*	CBG01004	3	135.39794	Unambiguous reciprocal BLAST	Supported by synteny	Confirmed by Treefam	
WBGene00007413	*pro-2*	CBG02729	3	Inf	Unambiguous reciprocal BLAST	Supported by synteny	Confirmed by Treefam	
WBGene00010015	*atad-3*	CBG18727	3	Inf	Unambiguous reciprocal BLAST	Supported by synteny	Confirmed by Treefam	
WBGene00004704	*rsp-7*	CBG03040	3	98.07918125	Unambiguous reciprocal BLAST	Supported by synteny	Confirmed by Treefam	
WBGene00000933	*dap-3*	CBG03062	3	Inf	Unambiguous reciprocal BLAST	Supported by synteny	Confirmed by Treefam	
WBGene00004185	*pro-1*	CBG03075	3	Inf	Unambiguous reciprocal BLAST	Supported by synteny	Confirmed by Treefam	
WBGene00007412	*stip-1*	CBG02731	3	Inf	Unambiguous reciprocal BLAST	Supported by synteny	Confirmed by Treefam	
WBGene00010896	*M28.5*	CBG03041	3	62.60205999	Unambiguous reciprocal BLAST	Supported by synteny	Confirmed by Treefam	
WBGene00000798	*crn-5*	CBG03059	3	130.09691	Unambiguous reciprocal BLAST	Supported by synteny	Confirmed by Treefam	
WBGene00011527	*cchl-1*	CBG02944	3	Inf	Unambiguous reciprocal BLAST	Supported by synteny	Confirmed by Treefam	
WBGene00008256	*glb-12*	CBG03023	3	Inf	Unambiguous reciprocal BLAST	Supported by synteny	Not confirmed by Treefam	
WBGene00003367	*mix-1*	CBG03006	3	Inf	Unambiguous reciprocal BLAST	Supported by synteny	Confirmed by Treefam	
WBGene00011067	*vps-11*	CBG03015	3	Inf	Unambiguous reciprocal BLAST	Supported by synteny	Confirmed by Treefam	
WBGene00009353	*sdhd-1*	CBG02978	3	98.30103	Unambiguous reciprocal BLAST	Supported by synteny	Confirmed by Treefam	
WBGene00000411	*cdl-1*	CBG03013	3	Inf	Unambiguous reciprocal BLAST	Supported by synteny	Confirmed by Treefam	
WBGene00000293	*cap-2*	CBG03000	3	Inf	Unambiguous reciprocal BLAST	Supported by synteny	Confirmed by Treefam	
WBGene00002957	*let-858*	CBG02984	3	Inf	Unambiguous reciprocal BLAST	Supported by synteny	Confirmed by Treefam	
WBGene00003831	*nuo-1*	CBG02970	3	Inf	Unambiguous reciprocal BLAST	Supported by synteny	Confirmed by Treefam	

WBGene00011526	*T06D8.5*	CBG02945	3	Inf	Unambiguous reciprocal BLAST	Supported by synteny	Confirmed by Treefam	
WBGene00009701	*egg-3*	CBG20647	3	Inf	Unambiguous reciprocal BLAST	Not supported by synteny	Confirmed by Treefam	
WBGene00004465	*rpn-9*	CBG02942	3	Inf	Unambiguous reciprocal BLAST	Supported by synteny	Confirmed by Treefam	
WBGene00012148	*VF13D12L.1*	CBG03142	3	Inf	Unambiguous reciprocal BLAST	Supported by synteny	Confirmed by Treefam	
WBGene00010054	*F54D5.11*	CBG02864	3	Inf	Unambiguous reciprocal BLAST	Supported by synteny	Confirmed by Treefam	
WBGene00012204	*W02B12.9*	CBG02884	3	Inf	Unambiguous reciprocal BLAST	Supported by synteny	Confirmed by Treefam	
WBGene00008361	*D1043.1*	CBG02853	3	Inf	Unambiguous reciprocal BLAST	Supported by synteny	Confirmed by Treefam	
WBGene00007192	*B0491.5*	CBG02921	3	Inf	Unambiguous reciprocal BLAST	Supported by synteny	Confirmed by Treefam	
WBGene00009688	*F44E5.1*	CBG03126	3	33.1949766	Unambiguous reciprocal BLAST	Supported by synteny	Confirmed by Treefam	
WBGene00013267	*Y57A10A.27*	CBG20930	3	174.9542425	Unambiguous reciprocal BLAST	Supported by synteny	Not confirmed by Treefam	
WBGene00003789	*npp-3*	CBG02744	3	Inf	Unambiguous reciprocal BLAST	Supported by synteny	Confirmed by Treefam	
WBGene00001679	*gpb-1*	CBG03131	3	Inf	Unambiguous reciprocal BLAST	Supported by synteny	Confirmed by Treefam	
WBGene00014153	*vab-23*	CBG02749	3	131.1249387	Unambiguous reciprocal BLAST	Supported by synteny	Confirmed by Treefam	
WBGene00003154	*mcm-2*	CBG18436	3	Inf	Unambiguous reciprocal BLAST	Not supported by synteny	Confirmed by Treefam	
WBGene00009661	*patr-1*	CBG03125	3	Inf	Unambiguous reciprocal BLAST	Supported by synteny	Not confirmed by Treefam	
WBGene00009246	*F29C12.4*	CBG02783	3	Inf	Unambiguous reciprocal BLAST	Supported by synteny	Confirmed by Treefam	
WBGene00010266	*dct-18*	CBG02759	3	130.30103	Unambiguous reciprocal BLAST	Not supported by synteny	Confirmed by Treefam	
WBGene00012602	*Y38E10A.24*	CBG07913	3	38.22184875	Unambiguous reciprocal BLAST	Supported by synteny	Not confirmed by Treefam	
WBGene00010267	*lips-9*	CBG02760	3	Inf	Unambiguous reciprocal BLAST	Supported by synteny	Confirmed by Treefam	
WBGene00013004	*Y48E1B.5*	CBG20758	3	Inf	Unambiguous reciprocal BLAST	Supported by synteny	Confirmed by Treefam	
WBGene00012984	*Y48B6A.13*	CBG20661	3	Inf	Unambiguous reciprocal BLAST	Supported by synteny	Confirmed by Treefam	
WBGene00012187	*rpb-11*	CBG20834	3	67.85387196	Unambiguous reciprocal BLAST	Supported by synteny	Confirmed by Treefam	
WBGene00012978	*Y48B6A.1*	CBG20902	3	Inf	Unambiguous reciprocal BLAST	Not supported by synteny	Confirmed by Treefam	
WBGene00006964	*xrn-2*	CBG20900	3	Inf	Unambiguous reciprocal BLAST	Supported by synteny	Confirmed by Treefam	
WBGene00004456	*rpl-43*	CBG20901	3	57.65321251	Unambiguous reciprocal BLAST	Supported by synteny	Confirmed by Treefam	
WBGene00012230	*cacn-1*	CBG20998	3	Inf	Unambiguous reciprocal BLAST	Supported by synteny	Confirmed by Treefam	
WBGene00011043	*R05H10.2*	CBG20878	3	Inf	Unambiguous reciprocal BLAST	Supported by synteny	Confirmed by Treefam	
WBGene00013168	*arp-1*	CBG03145	3	Inf	Unambiguous reciprocal BLAST	Not supported by synteny	Confirmed by Treefam	
WBGene00007999	*tag-297*	CBG11063	3	Inf	Unambiguous reciprocal BLAST	Not supported by synteny	Not confirmed by Treefam	
WBGene00001225	*eif-3.B*	CBG04247	3	Inf	Unambiguous reciprocal BLAST	Supported by synteny	Confirmed by Treefam	
WBGene00006381	*tac-1*	CBG04258	3	104.3853509	Unambiguous reciprocal BLAST	Supported by synteny	Not confirmed by Treefam	
WBGene00021546	*Y43H11AL.2*	CBG07028	3	Inf	Unambiguous reciprocal BLAST	Supported by synteny	Confirmed by Treefam	
WBGene00001793	*gsy-1*	CBG18401	3	Inf	Unambiguous reciprocal BLAST	Supported by synteny	Confirmed by Treefam	
WBGene00004166	*pqn-85*	CBG07027	3	Inf	Unambiguous reciprocal BLAST	Supported by synteny	Confirmed by Treefam	
WBGene00018782	*cct-3*	CBG21460	3	Inf	Unambiguous reciprocal BLAST	Not supported by synteny	Confirmed by Treefam	
WBGene00017328	*F10C5.2*	CBG15202	3	Inf	Unambiguous reciprocal BLAST	Supported by synteny	Confirmed by Treefam	
WBGene00021061	*W06E11.1*	CBG15253	3	Inf	Unambiguous reciprocal BLAST	Supported by synteny	Confirmed by Treefam	
WBGene00006781	*unc-45*	CBG15283	3	Inf	Unambiguous reciprocal BLAST	Supported by synteny	Confirmed by Treefam	
WBGene00003931	*pat-4*	CBG15792	3	Inf	Unambiguous reciprocal BLAST	Not supported by synteny	Confirmed by Treefam	
WBGene00044318	*tag-267*	CBG15252	3	96.23044892	Unambiguous reciprocal BLAST	Supported by synteny	Not confirmed by Treefam	
WBGene00004491	*rps-22*	CBG11742	3	23.39794001	Unambiguous reciprocal BLAST	Supported by synteny	Confirmed by Treefam	
WBGene00003917	*par-2*	CBG15155	3	Inf	Unambiguous reciprocal BLAST	Supported by synteny	Not confirmed by Treefam	
WBGene00004498	*rps-29*	CBG15290	3	32.69019608	Unambiguous reciprocal BLAST	Supported by synteny	Confirmed by Treefam	
WBGene00000263	*F23H11.5*	CBG15171	3	58.49136169	Unambiguous reciprocal BLAST	Supported by synteny	Confirmed by Treefam	
WBGene00022106	*lgc-46*	CBG11729	3	Inf	Unambiguous reciprocal BLAST	Supported by synteny	Confirmed by Treefam	
WBGene00021021	*W04B5.4*	CBG15659	3	130.5228787	Unambiguous reciprocal BLAST	Supported by synteny	Confirmed by Treefam	
WBGene00020105	*R148.7*	CBG20303	3	20.65321251	Unambiguous reciprocal BLAST	Supported by synteny	Not confirmed by Treefam	
WBGene00006725	*ubl-1*	CBG22466	3	73.77815125	Unambiguous reciprocal BLAST	Supported by synteny	Confirmed by Treefam	
WBGene00007927	*C34C12.8*	CBG09795	3	156.7781513	Unambiguous reciprocal BLAST	Not supported by synteny	Confirmed by Treefam	
WBGene00003930	*pat-3*	CBG03601	3	Inf	Unambiguous reciprocal BLAST	Supported by synteny	Confirmed by Treefam	
WBGene00011367	*T02C12.2*	CBG20195	3	Inf	Unambiguous reciprocal BLAST	Supported by synteny	Confirmed by Treefam	
WBGene00011111	*snfc-5*	CBG18075	3	Inf	Unambiguous reciprocal BLAST	Supported by synteny	Confirmed by Treefam	
WBGene00012858	*Y44F5A.1*	CBG24890	3	Inf	Unambiguous reciprocal BLAST	Not supported by synteny	Confirmed by Treefam	
WBGene00011605	*T08A11.2*	CBG21228	3	Inf	Unambiguous reciprocal BLAST	Supported by synteny	Confirmed by Treefam	
WBGene00011109	*R07E5.1*	CBG18072	3	Inf	Unambiguous reciprocal BLAST	Supported by synteny	Confirmed by Treefam	
WBGene00004392	*rnr-2*	CBG20205	3	Inf	Unambiguous reciprocal BLAST	Not supported by synteny	Confirmed by Treefam	
WBGene00003157	*mcm-5*	CBG12303	3	Inf	Unambiguous reciprocal BLAST	Supported by synteny	Confirmed by Treefam	
WBGene00009649	*F43C1.5*	CBG21243	3	53.75966784	Unambiguous reciprocal BLAST	Supported by synteny	Not confirmed by Treefam	
WBGene00011115	*R07E5.7*	CBG18066	3	99.26324143	Unambiguous reciprocal BLAST	Not supported by synteny	Confirmed by Treefam	
WBGene00010428	*dcn-1*	CBG18096	3	136.3521825	Unambiguous reciprocal BLAST	Supported by synteny	Confirmed by Treefam	

WBGene00003073	*lars-1*	CBG20159	3	Inf	Unambiguous reciprocal BLAST	Supported by synteny	Confirmed by Treefam	
WBGene00007627	*ccdc-55*	CBG20153	3	Inf	Unambiguous reciprocal BLAST	Supported by synteny	Confirmed by Treefam	
WBGene00001259	*emb-5*	CBG18001	3	Inf	Unambiguous reciprocal BLAST	Supported by synteny	Confirmed by Treefam	
WBGene00004387	*rnp-4*	CBG18074	3	80.14612804	Unambiguous reciprocal BLAST	Supported by synteny	Confirmed by Treefam	
WBGene00010905	*M88.2*	CBG18046	3	156.60206	Unambiguous reciprocal BLAST	Supported by synteny	Confirmed by Treefam	
WBGene00004874	*smc-4*	CBG18034	3	Inf	Unambiguous reciprocal BLAST	Supported by synteny	Confirmed by Treefam	
WBGene00011412	*T04A8.11*	CBG18006	3	150.4771213	Unambiguous reciprocal BLAST	Supported by synteny	Confirmed by Treefam	
WBGene00000206	*asb-1*	CBG18031	3	Inf	Unambiguous reciprocal BLAST	Supported by synteny	Confirmed by Treefam	
WBGene00004469	*rps-0*	CBG17994	3	Inf	Unambiguous reciprocal BLAST	Not supported by synteny	Confirmed by Treefam	
WBGene00007171	*B0393.6*	CBG17986	3	166.8239087	Unambiguous reciprocal BLAST	Supported by synteny	Confirmed by Treefam	
WBGene00011408	*T04A8.6*	CBG18012	3	116.5228787	Unambiguous reciprocal BLAST	Supported by synteny	Confirmed by Treefam	
WBGene00016439	*C35D10.1*	CBG17947	3	135.3117539	Unambiguous reciprocal BLAST	Supported by synteny	Confirmed by Treefam	
WBGene00006735	*ula-1*	CBG18190	3	Inf	Unambiguous reciprocal BLAST	Supported by synteny	Confirmed by Treefam	
WBGene00016676	*C45G9.5*	CBG23022	3	179.0280287	Unambiguous reciprocal BLAST	Supported by synteny	Not confirmed by Treefam	
WBGene00019628	*pup-2*	CBG03540	3	Inf	Unambiguous reciprocal BLAST	Supported by synteny	Confirmed by Treefam	
WBGene00001262	*emb-8*	CBG03543	3	Inf	Unambiguous reciprocal BLAST	Supported by synteny	Confirmed by Treefam	
WBGene00020092	*pcf-11*	CBG16521	3	Inf	Unambiguous reciprocal BLAST	Supported by synteny	Confirmed by Treefam	
WBGene00016443	*C35D10.6*	CBG17960	3	105.69897	Unambiguous reciprocal BLAST	Supported by synteny	Confirmed by Treefam	
WBGene00020423	*T10F2.4*	CBG21324	3	Inf	Unambiguous reciprocal BLAST	Supported by synteny	Confirmed by Treefam	
WBGene00021900	*Y54H5A.2*	CBG21314	3	57.82390874	Unambiguous reciprocal BLAST	Supported by synteny	Confirmed by Treefam	
WBGene00006736	*ulp-1*	CBG21323	3	Inf	Unambiguous reciprocal BLAST	Supported by synteny	Confirmed by Treefam	
WBGene00016170	*C27F2.8*	CBG16531	3	Inf	Unambiguous reciprocal BLAST	Supported by synteny	Confirmed by Treefam	
WBGene00018963	*ucr-1*	CBG08976	3	Inf	Unambiguous reciprocal BLAST	Supported by synteny	Confirmed by Treefam	
WBGene00001661	*gop-2*	CBG21182	3	Inf	Unambiguous reciprocal BLAST	Supported by synteny	Confirmed by Treefam	
WBGene00019836	*R02F2.7*	CBG05202	3	Inf	Unambiguous reciprocal BLAST	Not supported by synteny	Confirmed by Treefam	
WBGene00022598	*ztf-8*	CBG21193	3	Inf	Unambiguous reciprocal BLAST	Supported by synteny	Not confirmed by Treefam	
WBGene00003371	*mlc-3*	CBG24046	3	55.82390874	Unambiguous reciprocal BLAST	Supported by synteny	Confirmed by Treefam	
WBGene00003901	*paa-1*	CBG05190	3	Inf	Unambiguous reciprocal BLAST	Supported by synteny	Confirmed by Treefam	
WBGene00018609	*F48E8.2*	CBG05193	3	Inf	Unambiguous reciprocal BLAST	Supported by synteny	Confirmed by Treefam	
WBGene00004481	*rps-12*	CBG08991	3	89.39794001	Unambiguous reciprocal BLAST	Supported by synteny	Confirmed by Treefam	
WBGene00006943	*wrm-1*	CBG09006	3	Inf	Unambiguous reciprocal BLAST	Supported by synteny	Confirmed by Treefam	
WBGene00001869	*him-10*	CBG09087	3	170.0413927	Unambiguous reciprocal BLAST	Supported by synteny	Confirmed by Treefam	
WBGene00000182	*arf-1.2*	CBG09004	3	20.95424251	Unambiguous reciprocal BLAST	Supported by synteny	Confirmed by Treefam	
WBGene00015143	*B0336.3*	CBG09003	3	Inf	Unambiguous reciprocal BLAST	Supported by synteny	Confirmed by Treefam	
WBGene00004858	*sma-4*	CBG09090	3	Inf	Unambiguous reciprocal BLAST	Not supported by synteny	Confirmed by Treefam	
WBGene00004435	*rpl-23*	CBG09001	3	97.61978876	Unambiguous reciprocal BLAST	Supported by synteny	Confirmed by Treefam	
WBGene00017162	*ddx-23*	CBG09074	3	Inf	Unambiguous reciprocal BLAST	Supported by synteny	Confirmed by Treefam	
WBGene00015487	*C05D11.10*	CBG19669	3	111.3117539	Unambiguous reciprocal BLAST	Supported by synteny	Confirmed by Treefam	
WBGene00002855	*let-721*	CBG19676	3	Inf	Unambiguous reciprocal BLAST	Supported by synteny	Confirmed by Treefam	
WBGene00006516	*vps-16*	CBG19671	3	Inf	Unambiguous reciprocal BLAST	Supported by synteny	Confirmed by Treefam	
WBGene00001972	*hmg-1.2*	CBG17468	3	144	Unambiguous reciprocal BLAST	Supported by synteny	Confirmed by Treefam	
WBGene00015809	*C16A3.4*	CBG19691	3	Inf	Unambiguous reciprocal BLAST	Supported by synteny	Confirmed by Treefam	
WBGene00006515	*tag-170*	CBG19670	3	137.60206	Unambiguous reciprocal BLAST	Supported by synteny	Confirmed by Treefam	
WBGene00016015	*C23G10.8*	CBG24263	3	Inf	Unambiguous reciprocal BLAST	Supported by synteny	Confirmed by Treefam	
WBGene00001005	*dlc-1*	CBG19682	3	39.64781748	Unambiguous reciprocal BLAST	Supported by synteny	Confirmed by Treefam	
WBGene00015486	*C05D11.9*	CBG19668	3	Inf	Unambiguous reciprocal BLAST	Supported by synteny	Confirmed by Treefam	
WBGene00004472	*rps-3*	CBG24262	3	179.0413927	Unambiguous reciprocal BLAST	Supported by synteny	Confirmed by Treefam	
WBGene00020437	*T12A2.2*	CBG17485	3	Inf	Unambiguous reciprocal BLAST	Supported by synteny	Confirmed by Treefam	
WBGene00015811	*C16A3.6*	CBG19689	3	162.90309	Unambiguous reciprocal BLAST	Supported by synteny	Confirmed by Treefam	
WBGene00015810	*C16A3.5*	CBG19690	3	117.462398	Unambiguous reciprocal BLAST	Supported by synteny	Confirmed by Treefam	
WBGene00004781	*set-1*	CBG19678	3	23.17609126	Unambiguous reciprocal BLAST	Supported by synteny	Confirmed by Treefam	
WBGene00019380	*K04C2.2*	CBG18218	3	Inf	Unambiguous reciprocal BLAST	Supported by synteny	Confirmed by Treefam	
WBGene00015734	*C13B9.3*	CBG15197	3	Inf	Unambiguous reciprocal BLAST	Supported by synteny	Confirmed by Treefam	
WBGene00002169	*isw-1*	CBG16574	3	Inf	Unambiguous reciprocal BLAST	Supported by synteny	Confirmed by Treefam	
WBGene00020068	*cra-1*	CBG16540	3	Inf	Unambiguous reciprocal BLAST	Supported by synteny	Confirmed by Treefam	
WBGene00000276	*byn-1*	CBG16479	3	Inf	Unambiguous reciprocal BLAST	Supported by synteny	Confirmed by Treefam	
WBGene00000443	*ceh-20*	CBG16494	3	Inf	Unambiguous reciprocal BLAST	Supported by synteny	Confirmed by Treefam	
WBGene00020601	*T20B12.3*	CBG09137	3	Inf	Unambiguous reciprocal BLAST	Supported by synteny	Confirmed by Treefam	
WBGene00018151	*F37C12.3*	CBG08349	3	50.69897	Unambiguous reciprocal BLAST	Supported by synteny	Confirmed by Treefam	
WBGene00020636	*T20H4.5*	CBG24154	3	154.2340832	Unambiguous reciprocal BLAST	Supported by synteny	Confirmed by Treefam	

WBGene00015162	*B0361.8*	CBG15348	3	Inf	Unambiguous reciprocal BLAST	Supported by synteny	Confirmed by Treefam	
WBGene00019401	*nuo-4*	CBG16606	3	Inf	Unambiguous reciprocal BLAST	Supported by synteny	Confirmed by Treefam	
WBGene00018154	*exos-9*	CBG16619	3	Inf	Unambiguous reciprocal BLAST	Supported by synteny	Confirmed by Treefam	
WBGene00004483	*rps-14*	CBG08351	3	105.4149733	Unambiguous reciprocal BLAST	Not supported by synteny	Confirmed by Treefam	
WBGene00006542	*tbp-1*	CBG09138	3	Inf	Unambiguous reciprocal BLAST	Supported by synteny	Confirmed by Treefam	
WBGene00017268	*F08F8.2*	CBG15296	3	Inf	Unambiguous reciprocal BLAST	Supported by synteny	Confirmed by Treefam	
WBGene00015164	*ykt-6*	CBG15347	3	124.09691	Unambiguous reciprocal BLAST	Supported by synteny	Confirmed by Treefam	
WBGene00004417	*rpl-6*	CBG16622	3	144.8260748	Unambiguous reciprocal BLAST	Supported by synteny	Confirmed by Treefam	
WBGene00020604	*T20B12.7*	CBG09135	3	130.7403627	Unambiguous reciprocal BLAST	Supported by synteny	Confirmed by Treefam	
WBGene00020600	*T20B12.1*	CBG09139	3	Inf	Unambiguous reciprocal BLAST	Supported by synteny	Confirmed by Treefam	
WBGene00004490	*rps-21*	CBG16614	3	58.36797679	Unambiguous reciprocal BLAST	Supported by synteny	Confirmed by Treefam	
WBGene00018149	*F37C12.1*	CBG16611	3	Inf	Unambiguous reciprocal BLAST	Supported by synteny	Confirmed by Treefam	
WBGene00000479	*cgh-1*	CBG09177	3	Inf	Unambiguous reciprocal BLAST	Supported by synteny	Confirmed by Treefam	
WBGene00001209	*egl-45*	CBG00021	3	Inf	Unambiguous reciprocal BLAST	Supported by synteny	Confirmed by Treefam	
WBGene00022793	*ZK686.3*	CBG00031	3	Inf	Unambiguous reciprocal BLAST	Supported by synteny	Confirmed by Treefam	
WBGene00015591	*C08C3.4*	CBG00026	3	149.69897	Unambiguous reciprocal BLAST	Supported by synteny	Confirmed by Treefam	
WBGene00022803	*ZK688.9*	CBG12266	3	24.12493874	Unambiguous reciprocal BLAST	Not supported by synteny	Confirmed by Treefam	
WBGene00022792	*ZK686.2*	CBG00032	3	Inf	Unambiguous reciprocal BLAST	Supported by synteny	Confirmed by Treefam	
WBGene00016202	*kle-2*	CBG12291	3	Inf	Unambiguous reciprocal BLAST	Not supported by synteny	Confirmed by Treefam	
WBGene00003063	*lpd-7*	CBG09152	3	Inf	Unambiguous reciprocal BLAST	Supported by synteny	Confirmed by Treefam	
WBGene00018846	*eef-1B.1*	CBG22942	3	53	Unambiguous reciprocal BLAST	Supported by synteny	Confirmed by Treefam	
WBGene00004420	*rpl-9*	CBG09153	3	133.5563025	Unambiguous reciprocal BLAST	Supported by synteny	Confirmed by Treefam	
WBGene00003024	*lin-39*	CBG09167	3	97.69897	Unambiguous reciprocal BLAST	Supported by synteny	Confirmed by Treefam	
WBGene00022816	*fbn-1*	CBG00005	3	Inf	Unambiguous reciprocal BLAST	Supported by synteny	Confirmed by Treefam	
WBGene00004918	*snr-5*	CBG12250	3	34	Unambiguous reciprocal BLAST	Supported by synteny	Confirmed by Treefam	
WBGene00004449	*rpl-35*	CBG12256	3	68.67209786	Unambiguous reciprocal BLAST	Supported by synteny	Confirmed by Treefam	
WBGene00017319	*F09G8.3*	CBG16652	3	Inf	Unambiguous reciprocal BLAST	Supported by synteny	Confirmed by Treefam	
WBGene00002231	*knl-1*	CBG24615	3	32.57403127	Unambiguous reciprocal BLAST	Not supported by synteny	Not confirmed by Treefam	
WBGene00019678	*K12H4.3*	CBG22980	3	Inf	Unambiguous reciprocal BLAST	Supported by synteny	Confirmed by Treefam	
WBGene00000448	*ceh-26*	CBG22984	3	Inf	Unambiguous reciprocal BLAST	Supported by synteny	Confirmed by Treefam	
WBGene00003952	*pbs-6*	CBG24778	3	Inf	Unambiguous reciprocal BLAST	Not supported by synteny	Confirmed by Treefam	
WBGene00019680	*K12H4.5*	CBG22978	3	62.74036269	Unambiguous reciprocal BLAST	Not supported by synteny	Confirmed by Treefam	
WBGene00006840	*unc-116*	CBG18164	3	Inf	Unambiguous reciprocal BLAST	Not supported by synteny	Confirmed by Treefam	
WBGene00004726	*sas-4*	CBG24501	3	Inf	Unambiguous reciprocal BLAST	Not supported by synteny	Not confirmed by Treefam	
WBGene00019679	*K12H4.4*	CBG22979	3	110.2671717	Unambiguous reciprocal BLAST	Supported by synteny	Confirmed by Treefam	
WBGene00017356	*F10E9.4*	CBG16639	3	122.7231037	Unambiguous reciprocal BLAST	Supported by synteny	Not confirmed by Treefam	
WBGene00000143	*apc-2*	CBG22973	3	Inf	Unambiguous reciprocal BLAST	Supported by synteny	Confirmed by Treefam	
WBGene00004125	*mdt-30*	CBG22958	3	98.26717173	Unambiguous reciprocal BLAST	Supported by synteny	Not confirmed by Treefam	
WBGene00017044	*D2007.4*	CBG16676	3	115.447158	Unambiguous reciprocal BLAST	Supported by synteny	Confirmed by Treefam	
WBGene00004187	*prp-8*	CBG16670	3	Inf	Unambiguous reciprocal BLAST	Supported by synteny	Confirmed by Treefam	
WBGene00004433	*rpl-21*	CBG16683	3	114.0413927	Unambiguous reciprocal BLAST	Supported by synteny	Confirmed by Treefam	
WBGene00004460	*rpn-3*	CBG18132	3	Inf	Unambiguous reciprocal BLAST	Supported by synteny	Confirmed by Treefam	
WBGene00022864	*ZK1236.5*	CBG18136	3	70.22184875	Unambiguous reciprocal BLAST	Supported by synteny	Not confirmed by Treefam	
WBGene00016250	*C30C11.4*	CBG18133	3	Inf	Unambiguous reciprocal BLAST	Supported by synteny	Confirmed by Treefam	
WBGene00016249	*C30C11.1*	CBG18131	3	82.97312785	Unambiguous reciprocal BLAST	Supported by synteny	Confirmed by Treefam	
WBGene00015133	*B0303.15*	CBG15344	3	135.5228787	Unambiguous reciprocal BLAST	Supported by synteny	Confirmed by Treefam	
WBGene00015525	*rha-2*	CBG15333	3	Inf	Unambiguous reciprocal BLAST	Supported by synteny	Confirmed by Treefam	
WBGene00019305	*snap-29*	CBG06912	3	Inf	Unambiguous reciprocal BLAST	Supported by synteny	Confirmed by Treefam	
WBGene00004391	*rnr-1*	CBG10154	3	Inf	Unambiguous reciprocal BLAST	Not supported by synteny	Confirmed by Treefam	
WBGene00011143	*R08D7.2*	CBG06841	3	Inf	Unambiguous reciprocal BLAST	Supported by synteny	Confirmed by Treefam	
WBGene00001227	*eif-3.D*	CBG06842	3	Inf	Unambiguous reciprocal BLAST	Supported by synteny	Confirmed by Treefam	
WBGene00002081	*ina-1*	CBG06805	3	Inf	Unambiguous reciprocal BLAST	Supported by synteny	Confirmed by Treefam	
WBGene00011142	*R08D7.1*	CBG06840	3	Inf	Unambiguous reciprocal BLAST	Supported by synteny	Confirmed by Treefam	
WBGene00008514	*F02A9.4*	CBG06810	3	Inf	Unambiguous reciprocal BLAST	Supported by synteny	Confirmed by Treefam	
WBGene00001385	*far-1*	CBG06812	3	37.60205999	Unambiguous reciprocal BLAST	Supported by synteny	Confirmed by Treefam	
WBGene00001570	*gei-13*	CBG10038	3	Inf	Unambiguous reciprocal BLAST	Supported by synteny	Confirmed by Treefam	
WBGene00010038	*rheb-1*	CBG10088	3	72.77815125	Unambiguous reciprocal BLAST	Supported by synteny	Confirmed by Treefam	
WBGene00000235	*baf-1*	CBG10084	3	59.45863785	Unambiguous reciprocal BLAST	Supported by synteny	Confirmed by Treefam	
WBGene00007400	*C07A9.2*	CBG10019	3	105.5910646	Unambiguous reciprocal BLAST	Supported by synteny	Confirmed by Treefam	
WBGene00001094	*dars-1*	CBG10076	3	Inf	Unambiguous reciprocal BLAST	Supported by synteny	Confirmed by Treefam	

WBGene00012059	*T26G10.1*	CBG10097	3	Inf	Unambiguous reciprocal BLAST	Supported by synteny	Confirmed by Treefam	
WBGene00001284	*emb-30*	CBG10093	3	Inf	Unambiguous reciprocal BLAST	Supported by synteny	Confirmed by Treefam	
WBGene00014224	*ZK1098.7*	CBG10064	3	84.92081875	Unambiguous reciprocal BLAST	Supported by synteny	Confirmed by Treefam	
WBGene00000405	*cdk-1*	CBG10007	3	Inf	Unambiguous reciprocal BLAST	Supported by synteny	Confirmed by Treefam	
WBGene00004180	*pri-1*	CBG10030	3	Inf	Unambiguous reciprocal BLAST	Supported by synteny	Not confirmed by Treefam	
WBGene00006910	*vha-1*	CBG10001	3	23.34678749	Unambiguous reciprocal BLAST	Not supported by synteny	Confirmed by Treefam	
WBGene00000833	*cts-1*	CBG09810	3	Inf	Unambiguous reciprocal BLAST	Supported by synteny	Confirmed by Treefam	
WBGene00011867	*chc-1*	CBG09806	3	Inf	Unambiguous reciprocal BLAST	Supported by synteny	Confirmed by Treefam	
WBGene00014011	*ZK632.2*	CBG09993	3	Inf	Unambiguous reciprocal BLAST	Supported by synteny	Confirmed by Treefam	
WBGene00011806	*ekl-6*	CBG09896	3	Inf	Unambiguous reciprocal BLAST	Supported by synteny	Confirmed by Treefam	
WBGene00011814	*T16H12.4*	CBG09887	3	Inf	Unambiguous reciprocal BLAST	Supported by synteny	Confirmed by Treefam	
WBGene00006783	*unc-47*	CBG09800	3	Inf	Unambiguous reciprocal BLAST	Supported by synteny	Confirmed by Treefam	
WBGene00012359	*W09D10.1*	CBG18321	3	Inf	Unambiguous reciprocal BLAST	Supported by synteny	Confirmed by Treefam	
WBGene00003225	*mev-1*	CBG24607	3	116.9472936	Unambiguous reciprocal BLAST	Supported by synteny	Confirmed by Treefam	
WBGene00001002	*div-1*	CBG23795	3	Inf	Unambiguous reciprocal BLAST	Supported by synteny	Confirmed by Treefam	
WBGene00011559	*umps-1*	CBG24564	3	Inf	Unambiguous reciprocal BLAST	Supported by synteny	Confirmed by Treefam	
WBGene00010844	*M03C11.7*	CBG18389	3	Inf	Unambiguous reciprocal BLAST	Supported by synteny	Confirmed by Treefam	
WBGene00010476	*rnf-113*	CBG18314	3	171.7781513	Unambiguous reciprocal BLAST	Supported by synteny	Confirmed by Treefam	
WBGene00012977	*Y48A6C.4*	CBG13183	3	Inf	Unambiguous reciprocal BLAST	Not supported by synteny	Not confirmed by Treefam	
WBGene00004302	*ran-1*	CBG18317	3	132.60206	Unambiguous reciprocal BLAST	Supported by synteny	Confirmed by Treefam	
WBGene00012966	*exos-1*	CBG18230	3	139.0669468	Unambiguous reciprocal BLAST	Supported by synteny	Confirmed by Treefam	
WBGene00012964	*Y48A6B.3*	CBG18231	3	92.69897	Unambiguous reciprocal BLAST	Not supported by synteny	Confirmed by Treefam	
WBGene00013237	*Y56A3A.19*	CBG13312	3	56.90308999	Unambiguous reciprocal BLAST	Supported by synteny	Confirmed by Treefam	
WBGene00007703	*gbf-1*	CBG13224	3	Inf	Unambiguous reciprocal BLAST	Supported by synteny	Confirmed by Treefam	
WBGene00013544	*Y75B8A.7*	CBG23016	3	Inf	Unambiguous reciprocal BLAST	Supported by synteny	Confirmed by Treefam	
WBGene00001235	*elb-1*	CBG13236	3	59.49485002	Unambiguous reciprocal BLAST	Supported by synteny	Confirmed by Treefam	
WBGene00012756	*Y41C4A.9*	CBG13237	3	Inf	Unambiguous reciprocal BLAST	Supported by synteny	Confirmed by Treefam	
WBGene00006937	*wah-1*	CBG13330	3	Inf	Unambiguous reciprocal BLAST	Supported by synteny	Confirmed by Treefam	
WBGene00012554	*Y37D8A.16*	CBG15761	3	78.39794001	Unambiguous reciprocal BLAST	Not supported by synteny	Not confirmed by Treefam	
WBGene00013558	*Y75B8A.25*	CBG22546	3	65.47712125	Unambiguous reciprocal BLAST	Not supported by synteny	Not confirmed by Treefam	
WBGene00012550	*Y37D8A.10*	CBG15767	3	118.1356626	Unambiguous reciprocal BLAST	Supported by synteny	Confirmed by Treefam	
WBGene00012556	*Y37D8A.18*	CBG15759	3	100.0413927	Unambiguous reciprocal BLAST	Supported by synteny	Confirmed by Treefam	
WBGene00013029	*glrx-5*	CBG11774	3	55	Unambiguous reciprocal BLAST	Not supported by synteny	Confirmed by Treefam	
WBGene00013737	*tpst-1*	CBG03560	3	Inf	Unambiguous reciprocal BLAST	Supported by synteny	Confirmed by Treefam	
WBGene00013038	*ani-1*	CBG11691	3	Inf	Unambiguous reciprocal BLAST	Not supported by synteny	Confirmed by Treefam	
WBGene00012553	*cco-2*	CBG15765	3	119.69897	Unambiguous reciprocal BLAST	Supported by synteny	Confirmed by Treefam	
WBGene00013040	*Y49E10.21*	CBG11693	3	107.6720979	Unambiguous reciprocal BLAST	Supported by synteny	Not confirmed by Treefam	
WBGene00012714	*abce-1*	CBG22999	3	Inf	Unambiguous reciprocal BLAST	Supported by synteny	Confirmed by Treefam	
WBGene00002229	*klp-19*	CBG01568	3	Inf	Unambiguous reciprocal BLAST	Not supported by synteny	Confirmed by Treefam	
WBGene00000774	*cpf-2*	CBG03568	3	126.8908555	Unambiguous reciprocal BLAST	Supported by synteny	Confirmed by Treefam	
WBGene00003047	*lis-1*	CBG21225	3	Inf	Unambiguous reciprocal BLAST	Supported by synteny	Confirmed by Treefam	
WBGene00014177	*frg-1*	CBG15774	3	164.6892102	Unambiguous reciprocal BLAST	Supported by synteny	Confirmed by Treefam	
WBGene00003829	*nud-1*	CBG24281	3	Inf	Unambiguous reciprocal BLAST	Not supported by synteny	Confirmed by Treefam	
WBGene00006391	*taf-9*	CBG18251	3	106.3467875	Unambiguous reciprocal BLAST	Supported by synteny	Confirmed by Treefam	
WBGene00011734	*T12D8.6*	CBG18252	3	50.57403127	Unambiguous reciprocal BLAST	Supported by synteny	Confirmed by Treefam	
WBGene00001236	*elc-1*	CBG11707	3	44	Unambiguous reciprocal BLAST	Supported by synteny	Confirmed by Treefam	
WBGene00011729	*set-16*	CBG18244	3	Inf	Unambiguous reciprocal BLAST	Supported by synteny	Confirmed by Treefam	
WBGene00000875	*cyk-4*	CBG18263	3	Inf	Unambiguous reciprocal BLAST	Supported by synteny	Confirmed by Treefam	
WBGene00004075	*pod-1*	CBG15309	3	Inf	Unambiguous reciprocal BLAST	Supported by synteny	Confirmed by Treefam	
WBGene00022348	*Y82E9BR.16*	CBG11705	3	Inf	Unambiguous reciprocal BLAST	Supported by synteny	Confirmed by Treefam	
WBGene00021800	*Y53G8AL.2*	CBG20301	3	Inf	Unambiguous reciprocal BLAST	Supported by synteny	Confirmed by Treefam	
WBGene00022185	*Y71H2AM.20*	CBG09818	3	Inf	Unambiguous reciprocal BLAST	Supported by synteny	Confirmed by Treefam	
WBGene00022169	*Y71H2AM.4*	CBG09820	3	80.42324587	Unambiguous reciprocal BLAST	Supported by synteny	Confirmed by Treefam	
WBGene00022182	*Y71H2AM.17*	CBG09815	3	158.39794	Unambiguous reciprocal BLAST	Supported by synteny	Confirmed by Treefam	
WBGene00019298	*K02D7.1*	CBG13501	3	Inf	Unambiguous reciprocal BLAST	Supported by synteny	Confirmed by Treefam	
WBGene00003932	*pat-6*	CBG22549	3	Inf	Unambiguous reciprocal BLAST	Supported by synteny	Confirmed by Treefam	
WBGene00022045	*tag-313*	CBG13496	3	70.10551018	Unambiguous reciprocal BLAST	Supported by synteny	Confirmed by Treefam	
WBGene00004493	*rps-24*	CBG13547	3	78.30103	Unambiguous reciprocal BLAST	Not supported by synteny	Confirmed by Treefam	
WBGene00001071	*dpy-9*	CBG22547	3	109.30103	Unambiguous reciprocal BLAST	Supported by synteny	Not confirmed by Treefam	
WBGene00004981	*spl-1*	CBG10544	3	Inf	Unambiguous reciprocal BLAST	Supported by synteny	Confirmed by Treefam	

WBGene00019821	*R02D3.3*	CBG01668	3	Inf	Unambiguous reciprocal BLAST	Supported by synteny	Confirmed by Treefam	
WBGene00020296	*T07A9.8*	CBG13492	3	164.30103	Unambiguous reciprocal BLAST	Supported by synteny	Confirmed by Treefam	
WBGene00019823	*fnta-1*	CBG01666	3	Inf	Unambiguous reciprocal BLAST	Supported by synteny	Confirmed by Treefam	
WBGene00001579	*gex-2*	CBG01612	3	Inf	Unambiguous reciprocal BLAST	Supported by synteny	Confirmed by Treefam	
WBGene00022046	*Y66H1A.4*	CBG13541	3	50.81291336	Unambiguous reciprocal BLAST	Supported by synteny	Confirmed by Treefam	
WBGene00018932	*F56B3.8*	CBG23904	3	Inf	Unambiguous reciprocal BLAST	Supported by synteny	Confirmed by Treefam	
WBGene00018928	*F56B3.2*	CBG10721	3	144.30103	Unambiguous reciprocal BLAST	Supported by synteny	Confirmed by Treefam	
WBGene00016636	*C44B12.1*	CBG13961	3	101.4866666	Unambiguous reciprocal BLAST	Supported by synteny	Not confirmed by Treefam	
WBGene00009521	*F38A1.8*	CBG13942	3	Inf	Unambiguous reciprocal BLAST	Supported by synteny	Confirmed by Treefam	
WBGene00018776	*F53H1.1*	CBG16678	3	Inf	Unambiguous reciprocal BLAST	Not supported by synteny	Not confirmed by Treefam	
WBGene00016638	*C44B12.5*	CBG13962	3	105.8129134	Unambiguous reciprocal BLAST	Supported by synteny	Not confirmed by Treefam	
WBGene00003947	*pbs-1*	CBG13586	3	143.5228787	Unambiguous reciprocal BLAST	Supported by synteny	Confirmed by Treefam	
WBGene00019778	*M57.2*	CBG15026	3	Inf	Unambiguous reciprocal BLAST	Supported by synteny	Confirmed by Treefam	
WBGene00015413	*C04C3.3*	CBG21051	3	Inf	Unambiguous reciprocal BLAST	Supported by synteny	Confirmed by Treefam	
WBGene00021952	*vha-19*	CBG21042	3	Inf	Unambiguous reciprocal BLAST	Not supported by synteny	Confirmed by Treefam	
WBGene00000123	*ama-1*	CBG05355	3	Inf	Unambiguous reciprocal BLAST	Supported by synteny	Confirmed by Treefam	
WBGene00004432	*rpl-20*	CBG01745	3	132.7533277	Unambiguous reciprocal BLAST	Supported by synteny	Confirmed by Treefam	
WBGene00017119	*E04A4.5*	CBG01742	3	126.3679768	Unambiguous reciprocal BLAST	Supported by synteny	Not confirmed by Treefam	
WBGene00017925	*F29B9.11*	CBG01726	3	50.50965048	Unambiguous reciprocal BLAST	Supported by synteny	Not confirmed by Treefam	
WBGene00018898	*F55F10.1*	CBG01673	3	Inf	Unambiguous reciprocal BLAST	Not supported by synteny	Confirmed by Treefam	
WBGene00006706	*ubc-9*	CBG01720	3	90.69897	Unambiguous reciprocal BLAST	Supported by synteny	Confirmed by Treefam	
WBGene00017924	*F29B9.10*	CBG01725	3	52.27300127	Unambiguous reciprocal BLAST	Supported by synteny	Confirmed by Treefam	
WBGene00015330	*C02B10.5*	CBG19977	3	73	Unambiguous reciprocal BLAST	Supported by synteny	Confirmed by Treefam	
WBGene00002247	*lam-1*	CBG20003	3	Inf	Unambiguous reciprocal BLAST	Not supported by synteny	Confirmed by Treefam	
WBGene00016955	*C55C3.5*	CBG19878	3	Inf	Unambiguous reciprocal BLAST	Not supported by synteny	Confirmed by Treefam	
WBGene00001244	*elo-6*	CBG19953	3	Inf	Unambiguous reciprocal BLAST	Not supported by synteny	Confirmed by Treefam	
WBGene00001411	*fem-1*	CBG19924	3	Inf	Unambiguous reciprocal BLAST	Supported by synteny	Confirmed by Treefam	
WBGene00021156	*Y4C6B.2*	CBG19965	3	Inf	Unambiguous reciprocal BLAST	Not supported by synteny	Confirmed by Treefam	
WBGene00004804	*skn-1*	CBG19887	3	174.6320232	Unambiguous reciprocal BLAST	Supported by synteny	Not confirmed by Treefam	
WBGene00001243	*elo-5*	CBG19955	3	Inf	Unambiguous reciprocal BLAST	Supported by synteny	Confirmed by Treefam	
WBGene00019264	*H35B03.2*	CBG19874	3	Inf	Unambiguous reciprocal BLAST	Supported by synteny	Confirmed by Treefam	
WBGene00019154	*glf-1*	CBG19828	3	Inf	Unambiguous reciprocal BLAST	Supported by synteny	Confirmed by Treefam	
WBGene00004494	*rps-25*	CBG09459	3	78.54406804	Unambiguous reciprocal BLAST	Supported by synteny	Confirmed by Treefam	
WBGene00020683	*T22D1.4*	CBG05717	3	Inf	Unambiguous reciprocal BLAST	Supported by synteny	Confirmed by Treefam	
WBGene00006917	*vha-8*	CBG05372	3	156.4960066	Unambiguous reciprocal BLAST	Supported by synteny	Confirmed by Treefam	
WBGene00020687	*ruvb-2*	CBG05719	3	Inf	Unambiguous reciprocal BLAST	Supported by synteny	Confirmed by Treefam	
WBGene00015927	*dyci-1*	CBG05374	3	Inf	Unambiguous reciprocal BLAST	Not supported by synteny	Confirmed by Treefam	
WBGene00004458	*rpn-1*	CBG05716	3	Inf	Unambiguous reciprocal BLAST	Not supported by synteny	Confirmed by Treefam	
WBGene00001862	*him-3*	CBG02239	3	45.17609126	Unambiguous reciprocal BLAST	Not supported by synteny	Confirmed by Treefam	
WBGene00000585	*cogc-2*	CBG00414	3	Inf	Unambiguous reciprocal BLAST	Supported by synteny	Confirmed by Treefam	
WBGene00018350	*F42C5.10*	CBG05835	3	Inf	Unambiguous reciprocal BLAST	Supported by synteny	Confirmed by Treefam	
WBGene00000292	*cap-1*	CBG05812	3	Inf	Unambiguous reciprocal BLAST	Supported by synteny	Confirmed by Treefam	
WBGene00004477	*rps-8*	CBG05833	3	146.8129134	Unambiguous reciprocal BLAST	Supported by synteny	Confirmed by Treefam	
WBGene00004411	*rpc-1*	CBG05787	3	Inf	Unambiguous reciprocal BLAST	Supported by synteny	Confirmed by Treefam	
WBGene00016376	*sec-10*	CBG17671	3	Inf	Unambiguous reciprocal BLAST	Supported by synteny	Confirmed by Treefam	
WBGene00016374	*swd-2.2*	CBG17669	3	Inf	Unambiguous reciprocal BLAST	Supported by synteny	Confirmed by Treefam	
WBGene00016698	*C46A5.1*	CBG17666	3	104.5228787	Unambiguous reciprocal BLAST	Supported by synteny	Not confirmed by Treefam	
WBGene00002074	*ima-3*	CBG05912	3	Inf	Unambiguous reciprocal BLAST	Supported by synteny	Confirmed by Treefam	
WBGene00016844	*C50F7.4*	CBG00458	3	Inf	Unambiguous reciprocal BLAST	Not supported by synteny	Confirmed by Treefam	
WBGene00017989	*nol-10*	CBG05918	3	Inf	Unambiguous reciprocal BLAST	Supported by synteny	Confirmed by Treefam	
WBGene00017997	*F33D4.5*	CBG05941	3	Inf	Unambiguous reciprocal BLAST	Supported by synteny	Confirmed by Treefam	
WBGene00004471	*rps-2*	CBG17709	3	164.2304489	Unambiguous reciprocal BLAST	Supported by synteny	Not confirmed by Treefam	
WBGene00017643	*czw-1*	CBG17717	3	Inf	Unambiguous reciprocal BLAST	Supported by synteny	Confirmed by Treefam	
WBGene00017642	*F20D12.2*	CBG17718	3	Inf	Unambiguous reciprocal BLAST	Supported by synteny	Confirmed by Treefam	
WBGene00016793	*arp-11*	CBG17701	3	Inf	Unambiguous reciprocal BLAST	Supported by synteny	Confirmed by Treefam	
WBGene00004101	*hgrs-1*	CBG01567	3	Inf	Unambiguous reciprocal BLAST	Supported by synteny	Confirmed by Treefam	
WBGene00000183	*arf-3*	CBG17727	3	20.52287875	Unambiguous reciprocal BLAST	Supported by synteny	Confirmed by Treefam	
WBGene00020827	*T26A8.4*	CBG18471	3	125.6734159	Unambiguous reciprocal BLAST	Supported by synteny	Confirmed by Treefam	
WBGene00001051	*cks-1*	CBG15632	3	26	Unambiguous reciprocal BLAST	Supported by synteny	Confirmed by Treefam	
WBGene00001566	*acdh-13*	CBG18508	3	Inf	Unambiguous reciprocal BLAST	Supported by synteny	Confirmed by Treefam	

WBGene00009013	*F21D5.8*	CBG16434	3	86.34242268	Unambiguous reciprocal BLAST	Supported by synteny	Confirmed by Treefam	
WBGene00017075	*D2096.8*	CBG21270	3	139.4771213	Unambiguous reciprocal BLAST	Supported by synteny	Confirmed by Treefam	
WBGene00006374	*syx-4*	CBG08733	3	55.69897	Unambiguous reciprocal BLAST	Not supported by synteny	Confirmed by Treefam	
WBGene00017238	*F08B4.7*	CBG19656	3	54.47712125	Unambiguous reciprocal BLAST	Supported by synteny	Confirmed by Treefam	
WBGene00017237	*pole-2*	CBG19657	3	Inf	Unambiguous reciprocal BLAST	Supported by synteny	Confirmed by Treefam	
WBGene00008363	*D1046.2*	CBG21722	3	130.5228787	Unambiguous reciprocal BLAST	Supported by synteny	Confirmed by Treefam	
WBGene00009542	*F38E11.5*	CBG21666	3	Inf	Unambiguous reciprocal BLAST	Not supported by synteny	Not confirmed by Treefam	
WBGene00012433	*Y11D7A.9*	CBG15066	3	Inf	Unambiguous reciprocal BLAST	Not supported by synteny	Confirmed by Treefam	
WBGene00007880	*C33A12.1*	CBG20020	3	102.6690068	Unambiguous reciprocal BLAST	Supported by synteny	Confirmed by Treefam	
WBGene00004463	*rpn-7*	CBG21707	3	Inf	Unambiguous reciprocal BLAST	Supported by synteny	Confirmed by Treefam	
WBGene00009882	*vha-17*	CBG21703	3	51.41497335	Unambiguous reciprocal BLAST	Supported by synteny	Confirmed by Treefam	
WBGene00009881	*F49C12.12*	CBG21702	3	55.30103	Unambiguous reciprocal BLAST	Not supported by synteny	Confirmed by Treefam	
WBGene00010638	*K07F5.14*	CBG03469	3	171.39794	Unambiguous reciprocal BLAST	Supported by synteny	Confirmed by Treefam	
WBGene00009132	*F25H8.2*	CBG03454	3	167.455932	Unambiguous reciprocal BLAST	Supported by synteny	Confirmed by Treefam	
WBGene00011747	*sna-2*	CBG15011	3	Inf	Unambiguous reciprocal BLAST	Supported by synteny	Confirmed by Treefam	
WBGene00001395	*fat-3*	CBG03479	3	Inf	Unambiguous reciprocal BLAST	Supported by synteny	Confirmed by Treefam	
WBGene00008147	*C47E12.2*	CBG03436	3	Inf	Unambiguous reciprocal BLAST	Supported by synteny	Confirmed by Treefam	
WBGene00005663	*sars-1*	CBG03435	3	Inf	Unambiguous reciprocal BLAST	Supported by synteny	Confirmed by Treefam	
WBGene00008151	*C47E12.7*	CBG03442	3	Inf	Unambiguous reciprocal BLAST	Supported by synteny	Confirmed by Treefam	
WBGene00001188	*egl-20*	CBG03476	3	Inf	Unambiguous reciprocal BLAST	Supported by synteny	Confirmed by Treefam	
WBGene00003576	*ndc-80*	CBG23215	3	Inf	Unambiguous reciprocal BLAST	Not supported by synteny	Confirmed by Treefam	
WBGene00008506	*tkt-1*	CBG03385	3	Inf	Unambiguous reciprocal BLAST	Supported by synteny	Confirmed by Treefam	
WBGene00010556	*rack-1*	CBG03402	3	Inf	Unambiguous reciprocal BLAST	Supported by synteny	Confirmed by Treefam	
WBGene00001001	*dis-3*	CBG13499	3	Inf	Unambiguous reciprocal BLAST	Not supported by synteny	Confirmed by Treefam	
WBGene00011223	*R10H10.4*	CBG08380	3	29.34678749	Unambiguous reciprocal BLAST	Supported by synteny	Confirmed by Treefam	
WBGene00013857	*ZC168.3*	CBG17646	3	Inf	Unambiguous reciprocal BLAST	Supported by synteny	Confirmed by Treefam	
WBGene00003064	*lpd-8*	CBG24700	3	149.5185139	Unambiguous reciprocal BLAST	Supported by synteny	Confirmed by Treefam	
WBGene00004474	*rps-5*	CBG06011	3	151.0457575	Unambiguous reciprocal BLAST	Supported by synteny	Confirmed by Treefam	
WBGene00004199	*prx-14*	CBG06028	3	142.4819201	Unambiguous reciprocal BLAST	Supported by synteny	Confirmed by Treefam	
WBGene00011128	*R07H5.8*	CBG06032	3	Inf	Unambiguous reciprocal BLAST	Supported by synteny	Confirmed by Treefam	
WBGene00007443	*pfd-1*	CBG06021	3	28.17609126	Unambiguous reciprocal BLAST	Supported by synteny	Confirmed by Treefam	
WBGene00002344	*let-70*	CBG06002	3	66	Unambiguous reciprocal BLAST	Supported by synteny	Confirmed by Treefam	
WBGene00011481	*imp-2*	CBG06013	3	Inf	Unambiguous reciprocal BLAST	Supported by synteny	Confirmed by Treefam	
WBGene00003081	*lsm-7*	CBG05961	3	52.77815125	Unambiguous reciprocal BLAST	Supported by synteny	Confirmed by Treefam	
WBGene00010847	*M04B2.4*	CBG06141	3	Inf	Unambiguous reciprocal BLAST	Supported by synteny	Confirmed by Treefam	
WBGene00009588	*F40F11.3*	CBG06121	3	44.69897	Unambiguous reciprocal BLAST	Supported by synteny	Not confirmed by Treefam	
WBGene00004755	*sec-24.1*	CBG06129	3	Inf	Unambiguous reciprocal BLAST	Supported by synteny	Not confirmed by Treefam	
WBGene00014086	*ZK809.3*	CBG06101	3	152.462398	Unambiguous reciprocal BLAST	Supported by synteny	Confirmed by Treefam	
WBGene00009587	*F40F11.2*	CBG06120	3	Inf	Unambiguous reciprocal BLAST	Supported by synteny	Not confirmed by Treefam	
WBGene00003218	*mep-1*	CBG06138	3	Inf	Unambiguous reciprocal BLAST	Supported by synteny	Confirmed by Treefam	
WBGene00008722	*F12F6.7*	CBG06130	3	Inf	Unambiguous reciprocal BLAST	Supported by synteny	Confirmed by Treefam	
WBGene00010231	*F58B3.4*	CBG06109	3	Inf	Unambiguous reciprocal BLAST	Supported by synteny	Confirmed by Treefam	
WBGene00009039	*F22B3.8*	CBG03127	3	Inf	Unambiguous reciprocal BLAST	Not supported by synteny	Confirmed by Treefam	
WBGene00004480	*rps-11*	CBG06118	3	111	Unambiguous reciprocal BLAST	Supported by synteny	Confirmed by Treefam	
WBGene00011740	*T12G3.5*	CBG06212	3	142.6946052	Unambiguous reciprocal BLAST	Supported by synteny	Confirmed by Treefam	
WBGene00004192	*prx-2*	CBG06104	3	166.90309	Unambiguous reciprocal BLAST	Supported by synteny	Confirmed by Treefam	
WBGene00005015	*spt-5*	CBG06217	3	Inf	Unambiguous reciprocal BLAST	Supported by synteny	Confirmed by Treefam	
WBGene00003920	*par-5*	CBG06174	3	24.30103	Unambiguous reciprocal BLAST	Supported by synteny	Confirmed by Treefam	
WBGene00008683	*repo-1*	CBG06223	3	161.7781513	Unambiguous reciprocal BLAST	Supported by synteny	Confirmed by Treefam	
WBGene00008684	*mig-32*	CBG06226	3	Inf	Unambiguous reciprocal BLAST	Supported by synteny	Confirmed by Treefam	
WBGene00010890	*ddb-1*	CBG06232	3	Inf	Unambiguous reciprocal BLAST	Supported by synteny	Confirmed by Treefam	
WBGene00002368	*let-99*	CBG10071	3	Inf	Unambiguous reciprocal BLAST	Not supported by synteny	Confirmed by Treefam	
WBGene00004492	*rps-23*	CBG03324	3	93.12493874	Unambiguous reciprocal BLAST	Supported by synteny	Confirmed by Treefam	
WBGene00016581	*C42C1.3*	CBG06264	3	75.77815125	Unambiguous reciprocal BLAST	Supported by synteny	Confirmed by Treefam	
WBGene00004756	*sec-24.2*	CBG03317	3	Inf	Unambiguous reciprocal BLAST	Supported by synteny	Confirmed by Treefam	
WBGene00009211	*F28D1.1*	CBG03319	3	Inf	Unambiguous reciprocal BLAST	Supported by synteny	Confirmed by Treefam	
WBGene00016583	*tag-335*	CBG06266	3	Inf	Unambiguous reciprocal BLAST	Supported by synteny	Confirmed by Treefam	
WBGene00014083	*ZK795.3*	CBG13507	3	Inf	Unambiguous reciprocal BLAST	Supported by synteny	Confirmed by Treefam	
WBGene00000705	*col-131*	CBG05547	3	110.3679768	Unambiguous reciprocal BLAST	Not supported by synteny	Confirmed by Treefam	
WBGene00004315	*rbd-1*	CBG03493	3	Inf	Unambiguous reciprocal BLAST	Supported by synteny	Confirmed by Treefam	

WBGene00007987	*C36H8.1*	CBG01600	3	33.69897	Unambiguous reciprocal BLAST	Not supported by synteny	Confirmed by Treefam	
WBGene00010437	*JC8.5*	CBG01804	3	159.3710679	Unambiguous reciprocal BLAST	Supported by synteny	Confirmed by Treefam	
WBGene00013373	*Y62E10A.2*	CBG01782	3	51.3357921	Unambiguous reciprocal BLAST	Supported by synteny	Confirmed by Treefam	
WBGene00004424	*rpl-12*	CBG01806	3	116.0791812	Unambiguous reciprocal BLAST	Supported by synteny	Confirmed by Treefam	
WBGene00004339	*rfc-3*	CBG01827	3	Inf	Unambiguous reciprocal BLAST	Supported by synteny	Confirmed by Treefam	
WBGene00009926	*noah-2*	CBG18772	3	Inf	Unambiguous reciprocal BLAST	Supported by synteny	Confirmed by Treefam	
WBGene00012887	*Y45F10D.7*	CBG22378	3	Inf	Unambiguous reciprocal BLAST	Supported by synteny	Confirmed by Treefam	
WBGene00009925	*F52B11.2*	CBG18762	3	132.2174839	Unambiguous reciprocal BLAST	Supported by synteny	Confirmed by Treefam	
WBGene00012888	*sas-6*	CBG22377	3	Inf	Unambiguous reciprocal BLAST	Supported by synteny	Confirmed by Treefam	
WBGene00004487	*rps-18*	CBG21507	3	109.39794	Unambiguous reciprocal BLAST	Supported by synteny	Confirmed by Treefam	
WBGene00013308	*nuo-3*	CBG21512	3	87.92941893	Unambiguous reciprocal BLAST	Supported by synteny	Confirmed by Treefam	
WBGene00013808	*sfa-1*	CBG00421	3	Inf	Unambiguous reciprocal BLAST	Supported by synteny	Not confirmed by Treefam	
WBGene00006698	*uaf-2*	CBG00419	3	155.7781513	Unambiguous reciprocal BLAST	Supported by synteny	Confirmed by Treefam	
WBGene00004914	*snr-1*	CBG00427	3	84	Unambiguous reciprocal BLAST	Supported by synteny	Confirmed by Treefam	
WBGene00008707	*F11E6.3*	CBG00436	3	101.69897	Unambiguous reciprocal BLAST	Supported by synteny	Confirmed by Treefam	
WBGene00006920	*vha-11*	CBG16826	3	Inf	Unambiguous reciprocal BLAST	Supported by synteny	Confirmed by Treefam	
WBGene00001240	*elo-2*	CBG00438	3	Inf	Unambiguous reciprocal BLAST	Supported by synteny	Confirmed by Treefam	
WBGene00004497	*rps-28*	CBG13580	3	39.97772361	Unambiguous reciprocal BLAST	Supported by synteny	Confirmed by Treefam	
WBGene00021514	*Y41D4B.11*	CBG13570	3	149.0791812	Unambiguous reciprocal BLAST	Supported by synteny	Confirmed by Treefam	
WBGene00021934	*cct-8*	CBG08425	3	Inf	Unambiguous reciprocal BLAST	Supported by synteny	Confirmed by Treefam	
WBGene00022301	*cpsf-1*	CBG16808	3	Inf	Unambiguous reciprocal BLAST	Supported by synteny	Confirmed by Treefam	
WBGene00004496	*rps-27*	CBG06446	3	55.04139269	Unambiguous reciprocal BLAST	Supported by synteny	Confirmed by Treefam	
WBGene00021000	*W03F9.2*	CBG06454	3	45.69897	Unambiguous reciprocal BLAST	Not supported by synteny	Confirmed by Treefam	
WBGene00000552	*cmd-1*	CBG01097	3	36.57403127	Unambiguous reciprocal BLAST	Supported by synteny	Not confirmed by Treefam	
WBGene00004266	*rab-1*	CBG01077	3	73	Unambiguous reciprocal BLAST	Supported by synteny	Confirmed by Treefam	
WBGene00020392	*knl-3*	CBG21814	3	51.69897	Unambiguous reciprocal BLAST	Supported by synteny	Confirmed by Treefam	
WBGene00020340	*T08B1.1*	CBG12142	3	Inf	Unambiguous reciprocal BLAST	Not supported by synteny	Confirmed by Treefam	
WBGene00018762	*F53E10.6*	CBG06617	3	76.95424251	Unambiguous reciprocal BLAST	Supported by synteny	Confirmed by Treefam	
WBGene00019353	*K03B4.1*	CBG08796	3	Inf	Unambiguous reciprocal BLAST	Supported by synteny	Not confirmed by Treefam	
WBGene00016508	*C37H5.5*	CBG08826	3	Inf	Unambiguous reciprocal BLAST	Supported by synteny	Confirmed by Treefam	
WBGene00016062	*C24G6.8*	CBG09375	3	Inf	Unambiguous reciprocal BLAST	Supported by synteny	Confirmed by Treefam	
WBGene00003927	*pas-6*	CBG09365	3	140.69897	Unambiguous reciprocal BLAST	Supported by synteny	Confirmed by Treefam	
WBGene00016989	*CD4.3*	CBG09363	3	121.243038	Unambiguous reciprocal BLAST	Supported by synteny	Confirmed by Treefam	
WBGene00000159	*aps-1*	CBG09266	3	70.36797679	Unambiguous reciprocal BLAST	Supported by synteny	Confirmed by Treefam	
WBGene00000802	*crt-1*	CBG09253	3	Inf	Unambiguous reciprocal BLAST	Supported by synteny	Confirmed by Treefam	
WBGene00001073	*dpy-11*	CBG18956	3	132.09691	Unambiguous reciprocal BLAST	Supported by synteny	Confirmed by Treefam	
WBGene00020425	*syx-18*	CBG18985	3	155.9208188	Unambiguous reciprocal BLAST	Supported by synteny	Confirmed by Treefam	
WBGene00020950	*W02F12.5*	CBG19001	3	Inf	Unambiguous reciprocal BLAST	Supported by synteny	Confirmed by Treefam	
WBGene00020910	*W01A11.2*	CBG18949	3	Inf	Unambiguous reciprocal BLAST	Supported by synteny	Confirmed by Treefam	
WBGene00001513	*gad-1*	CBG18933	3	Inf	Unambiguous reciprocal BLAST	Supported by synteny	Confirmed by Treefam	
WBGene00020517	*T15B7.2*	CBG19031	3	143.30103	Unambiguous reciprocal BLAST	Supported by synteny	Confirmed by Treefam	
WBGene00007029	*mys-1*	CBG19077	3	Inf	Unambiguous reciprocal BLAST	Supported by synteny	Confirmed by Treefam	
WBGene00015464	*C05C8.7*	CBG19118	3	Inf	Unambiguous reciprocal BLAST	Not supported by synteny	Confirmed by Treefam	
WBGene00015743	*C13F10.4*	CBG19103	3	Inf	Unambiguous reciprocal BLAST	Supported by synteny	Confirmed by Treefam	
WBGene00017313	*cpsf-2*	CBG19097	3	Inf	Unambiguous reciprocal BLAST	Supported by synteny	Confirmed by Treefam	
WBGene00017605	*F19F10.9*	CBG22442	3	Inf	Unambiguous reciprocal BLAST	Supported by synteny	Confirmed by Treefam	
WBGene00002078	*xpo-1*	CBG20576	3	Inf	Unambiguous reciprocal BLAST	Supported by synteny	Confirmed by Treefam	
WBGene00017016	*snap-1*	CBG11346	3	169.7781513	Unambiguous reciprocal BLAST	Supported by synteny	Confirmed by Treefam	
WBGene00004766	*sel-9*	CBG11296	3	115.2218487	Unambiguous reciprocal BLAST	Supported by synteny	Confirmed by Treefam	
WBGene00006935	*vars-1*	CBG11384	3	Inf	Unambiguous reciprocal BLAST	Supported by synteny	Confirmed by Treefam	
WBGene00020717	*T23B12.2*	CBG01522	3	Inf	Unambiguous reciprocal BLAST	Supported by synteny	Confirmed by Treefam	
WBGene00001040	*dnj-22*	CBG01519	3	153.8239087	Unambiguous reciprocal BLAST	Supported by synteny	Confirmed by Treefam	
WBGene00020718	*T23B12.3*	CBG01521	3	168.3424227	Unambiguous reciprocal BLAST	Supported by synteny	Confirmed by Treefam	
WBGene00017797	*symk-1*	CBG01489	3	Inf	Unambiguous reciprocal BLAST	Supported by synteny	Confirmed by Treefam	
WBGene00003395	*mom-2*	CBG11282	3	Inf	Unambiguous reciprocal BLAST	Supported by synteny	Confirmed by Treefam	
WBGene00009103	*F25B3.6*	CBG19313	3	Inf	Unambiguous reciprocal BLAST	Supported by synteny	Confirmed by Treefam	
WBGene00003160	*mdf-1*	CBG19302	3	Inf	Unambiguous reciprocal BLAST	Supported by synteny	Not confirmed by Treefam	
WBGene00004320	*rbx-1*	CBG19342	3	49.69897	Unambiguous reciprocal BLAST	Supported by synteny	Confirmed by Treefam	
WBGene00007784	*ruvb-1*	CBG19422	3	Inf	Unambiguous reciprocal BLAST	Supported by synteny	Confirmed by Treefam	
WBGene00007097	*B0024.4*	CBG09578	3	71.82390874	Unambiguous reciprocal BLAST	Supported by synteny	Confirmed by Treefam	

WBGene00006489	*tag-143*	CBG09548	3	Inf	Unambiguous reciprocal BLAST	Supported by synteny	Confirmed by Treefam	
WBGene00010629	*K07C5.6*	CBG09591	3	Inf	Unambiguous reciprocal BLAST	Supported by synteny	Confirmed by Treefam	
WBGene00000200	*arx-2*	CBG09586	3	Inf	Unambiguous reciprocal BLAST	Supported by synteny	Confirmed by Treefam	
WBGene00014112	*ZK856.11*	CBG09545	3	94.74036269	Unambiguous reciprocal BLAST	Supported by synteny	Confirmed by Treefam	
WBGene00003065	*lpd-9*	CBG09647	3	34	Unambiguous reciprocal BLAST	Supported by synteny	Not confirmed by Treefam	
WBGene00008380	*D1054.14*	CBG09713	3	159.4336556	Unambiguous reciprocal BLAST	Supported by synteny	Confirmed by Treefam	
WBGene00002985	*lig-1*	CBG09716	3	Inf	Unambiguous reciprocal BLAST	Supported by synteny	Confirmed by Treefam	
WBGene00009242	*sre-6*	CBG09696	3	Inf	Unambiguous reciprocal BLAST	Supported by synteny	Confirmed by Treefam	
WBGene00010405	*H19N07.1*	CBG23104	3	Inf	Unambiguous reciprocal BLAST	Supported by synteny	Confirmed by Treefam	
WBGene00002072	*ima-1*	CBG23145	3	Inf	Unambiguous reciprocal BLAST	Supported by synteny	Confirmed by Treefam	
WBGene00009966	*F53B7.3*	CBG23070	3	178.0253059	Unambiguous reciprocal BLAST	Supported by synteny	Confirmed by Treefam	
WBGene00011831	*T19B10.2*	CBG23138	3	Inf	Unambiguous reciprocal BLAST	Supported by synteny	Confirmed by Treefam	
WBGene00007385	*atp-5*	CBG23108	3	136.2491984	Unambiguous reciprocal BLAST	Supported by synteny	Confirmed by Treefam	
WBGene00009385	*sas-5*	CBG23306	3	161.09691	Unambiguous reciprocal BLAST	Supported by synteny	Confirmed by Treefam	
WBGene00000453	*ceh-32*	CBG23282	3	Inf	Unambiguous reciprocal BLAST	Supported by synteny	Confirmed by Treefam	
WBGene00008338	*C55A6.9*	CBG23232	3	Inf	Unambiguous reciprocal BLAST	Supported by synteny	Confirmed by Treefam	
WBGene00006373	*syx-5*	CBG23272	3	Inf	Unambiguous reciprocal BLAST	Supported by synteny	Confirmed by Treefam	
WBGene00004917	*snr-4*	CBG23345	3	48.69897	Unambiguous reciprocal BLAST	Supported by synteny	Confirmed by Treefam	
WBGene00004501	*rpt-1*	CBG23346	3	Inf	Unambiguous reciprocal BLAST	Supported by synteny	Confirmed by Treefam	
WBGene00008645	*F10C2.4*	CBG23360	3	Inf	Unambiguous reciprocal BLAST	Supported by synteny	Confirmed by Treefam	
WBGene00001088	*dpy-30*	CBG23403	3	49.30103	Unambiguous reciprocal BLAST	Supported by synteny	Not confirmed by Treefam	
WBGene00000876	*cyl-1*	CBG23348	3	100.5228787	Unambiguous reciprocal BLAST	Supported by synteny	Not confirmed by Treefam	
WBGene00010204	*F57F5.1*	CBG23351	3	Inf	Unambiguous reciprocal BLAST	Supported by synteny	Confirmed by Treefam	
WBGene00005018	*sqt-3*	CBG23461	3	Inf	Unambiguous reciprocal BLAST	Supported by synteny	Confirmed by Treefam	
WBGene00010093	*capg-2*	CBG23429	3	Inf	Unambiguous reciprocal BLAST	Supported by synteny	Confirmed by Treefam	
WBGene00008452	*mrps-5*	CBG23528	3	Inf	Unambiguous reciprocal BLAST	Supported by synteny	Confirmed by Treefam	
WBGene00010097	*F55C5.8*	CBG23435	3	Inf	Unambiguous reciprocal BLAST	Supported by synteny	Confirmed by Treefam	
WBGene00012244	*W04D2.5*	CBG23508	3	134.09691	Unambiguous reciprocal BLAST	Supported by synteny	Confirmed by Treefam	
WBGene00009092	*tomm-20*	CBG23457	3	109.5228787	Unambiguous reciprocal BLAST	Supported by synteny	Confirmed by Treefam	
WBGene00007585	*C14C10.2*	CBG23537	3	148.8750613	Unambiguous reciprocal BLAST	Supported by synteny	Not confirmed by Treefam	
WBGene00007587	*C14C10.4*	CBG23538	3	Inf	Unambiguous reciprocal BLAST	Supported by synteny	Confirmed by Treefam	
WBGene00010094	*tsfm-1*	CBG23428	3	Inf	Unambiguous reciprocal BLAST	Supported by synteny	Confirmed by Treefam	
WBGene00011015	*R04F11.2*	CBG23452	3	56.99122608	Unambiguous reciprocal BLAST	Supported by synteny	Confirmed by Treefam	
WBGene00011247	*R11D1.9*	CBG08570	3	108.80618	Unambiguous reciprocal BLAST	Supported by synteny	Confirmed by Treefam	
WBGene00004738	*scc-3*	CBG08584	3	Inf	Unambiguous reciprocal BLAST	Supported by synteny	Confirmed by Treefam	
WBGene00008343	*C56A3.4*	CBG06679	3	125.6478175	Unambiguous reciprocal BLAST	Supported by synteny	Confirmed by Treefam	
WBGene00006311	*sun-1*	CBG17722	3	Inf	Unambiguous reciprocal BLAST	Not supported by synteny	Confirmed by Treefam	
WBGene00009347	*F32H5.1*	CBG17499	3	Inf	Unambiguous reciprocal BLAST	Supported by synteny	Confirmed by Treefam	
WBGene00009980	*F53F1.2*	CBG06721	3	67	Unambiguous reciprocal BLAST	Supported by synteny	Confirmed by Treefam	
WBGene00009993	*F53F4.11*	CBG11519	3	Inf	Unambiguous reciprocal BLAST	Supported by synteny	Confirmed by Treefam	
WBGene00010425	*lpin-1*	CBG11512	3	Inf	Unambiguous reciprocal BLAST	Supported by synteny	Confirmed by Treefam	
WBGene00009811	*F47B8.10*	CBG04634	3	Inf	Unambiguous reciprocal BLAST	Not supported by synteny	Confirmed by Treefam	
WBGene00013025	*vha-13*	CBG04632	3	Inf	Unambiguous reciprocal BLAST	Supported by synteny	Confirmed by Treefam	
WBGene00013024	*Y49A3A.1*	CBG04633	3	Inf	Unambiguous reciprocal BLAST	Supported by synteny	Confirmed by Treefam	
WBGene00007616	*C15H11.8*	CBG04618	3	74.34678749	Unambiguous reciprocal BLAST	Supported by synteny	Confirmed by Treefam	
WBGene00006836	*unc-112*	CBG04558	3	Inf	Unambiguous reciprocal BLAST	Supported by synteny	Confirmed by Treefam	
WBGene00009084	*F23B12.7*	CBG04610	3	Inf	Unambiguous reciprocal BLAST	Supported by synteny	Confirmed by Treefam	
WBGene00009082	*F23B12.5*	CBG04612	3	Inf	Unambiguous reciprocal BLAST	Supported by synteny	Confirmed by Treefam	
WBGene00008262	*ril-1*	CBG04589	3	87.462398	Unambiguous reciprocal BLAST	Supported by synteny	Confirmed by Treefam	
WBGene00009650	*F43D2.1*	CBG04574	3	142.7781513	Unambiguous reciprocal BLAST	Supported by synteny	Confirmed by Treefam	
WBGene00007617	*rrbs-1*	CBG04617	3	Inf	Unambiguous reciprocal BLAST	Supported by synteny	Confirmed by Treefam	
WBGene00011318	*cdt-2*	CBG11595	3	Inf	Unambiguous reciprocal BLAST	Supported by synteny	Confirmed by Treefam	
WBGene00001423	*fib-1*	CBG11588	3	162.7781513	Unambiguous reciprocal BLAST	Supported by synteny	Confirmed by Treefam	
WBGene00004485	*rps-16*	CBG11589	3	100.3521825	Unambiguous reciprocal BLAST	Supported by synteny	Confirmed by Treefam	
WBGene00004452	*rpl-38*	CBG18585	3	42.30103	Unambiguous reciprocal BLAST	Not supported by synteny	Confirmed by Treefam	
WBGene00011687	*T10C6.5*	CBG22646	3	107.366423	Unambiguous reciprocal BLAST	Supported by synteny	Confirmed by Treefam	
WBGene00012316	*W06H3.3*	CBG23896	3	Inf	Unambiguous reciprocal BLAST	Supported by synteny	Confirmed by Treefam	
WBGene00000776	*cpl-1*	CBG22599	3	Inf	Unambiguous reciprocal BLAST	Supported by synteny	Confirmed by Treefam	
WBGene00013343	*Y59A8B.6*	CBG05596	3	Inf	Unambiguous reciprocal BLAST	Supported by synteny	Confirmed by Treefam	
WBGene00001258	*emb-4*	CBG20315	3	129.6532125	Unambiguous reciprocal BLAST	Supported by synteny	Not confirmed by Treefam	

WBGene00006945	*wars-1*	CBG20320	3	83.84509804	Unambiguous reciprocal BLAST	Not supported by synteny	Confirmed by Treefam	
WBGene00012692	*Y39B6A.33*	CBG23810	3	113.1949766	Unambiguous reciprocal BLAST	Not supported by synteny	Confirmed by Treefam	
WBGene00012676	*pro-3*	CBG23585	3	Inf	Unambiguous reciprocal BLAST	Supported by synteny	Confirmed by Treefam	
WBGene00012830	*Y43F8C.8*	CBG05694	3	75.43933269	Unambiguous reciprocal BLAST	Supported by synteny	Confirmed by Treefam	
WBGene00004754	*sec-23*	CBG12918	3	Inf	Unambiguous reciprocal BLAST	Supported by synteny	Confirmed by Treefam	
WBGene00004413	*rpl-2*	CBG05588	3	Inf	Unambiguous reciprocal BLAST	Supported by synteny	Confirmed by Treefam	
WBGene00003164	*mdt-6*	CBG19070	3	124.462398	Unambiguous reciprocal BLAST	Supported by synteny	Confirmed by Treefam	
WBGene00009289	*exos-7*	CBG05573	3	166.4259687	Unambiguous reciprocal BLAST	Supported by synteny	Confirmed by Treefam	
WBGene00022021	*Y61A9LA.10*	CBG08772	3	Inf	Unambiguous reciprocal BLAST	Supported by synteny	Confirmed by Treefam	
WBGene00022861	*dve-1*	CBG08165	3	Inf	Unambiguous reciprocal BLAST	Not supported by synteny	Confirmed by Treefam	
WBGene00020215	*T04G9.4*	CBG08117	3	Inf	Unambiguous reciprocal BLAST	Supported by synteny	Confirmed by Treefam	
WBGene00003968	*peb-1*	CBG14059	3	Inf	Unambiguous reciprocal BLAST	Supported by synteny	Confirmed by Treefam	
WBGene00001065	*dpy-3*	CBG14077	3	25.30103	Unambiguous reciprocal BLAST	Supported by synteny	Not confirmed by Treefam	
WBGene00018703	*sec-3*	CBG14132	3	Inf	Unambiguous reciprocal BLAST	Supported by synteny	Confirmed by Treefam	
WBGene00015791	*C15C7.5*	CBG14145	3	73.90308999	Unambiguous reciprocal BLAST	Supported by synteny	Not confirmed by Treefam	
WBGene00000207	*asb-2*	CBG14472	3	Inf	Unambiguous reciprocal BLAST	Supported by synteny	Confirmed by Treefam	
WBGene00006983	*zig-6*	CBG14634	3	138	Unambiguous reciprocal BLAST	Supported by synteny	Not confirmed by Treefam	
WBGene00004438	*rpl-25.1*	CBG14529	3	31.60205999	Unambiguous reciprocal BLAST	Supported by synteny	Confirmed by Treefam	
WBGene00019760	*calu-1*	CBG14576	3	Inf	Unambiguous reciprocal BLAST	Supported by synteny	Confirmed by Treefam	
WBGene00003056	*lon-2*	CBG14534	3	Inf	Unambiguous reciprocal BLAST	Supported by synteny	Confirmed by Treefam	
WBGene00001070	*dpy-8*	CBG14618	3	Inf	Unambiguous reciprocal BLAST	Supported by synteny	Not confirmed by Treefam	
WBGene00000039	*acn-1*	CBG14607	3	Inf	Unambiguous reciprocal BLAST	Supported by synteny	Confirmed by Treefam	
WBGene00019759	*M03F4.6*	CBG14575	3	Inf	Unambiguous reciprocal BLAST	Supported by synteny	Confirmed by Treefam	
WBGene00004245	*puf-9*	CBG14762	3	Inf	Unambiguous reciprocal BLAST	Supported by synteny	Confirmed by Treefam	
WBGene00004788	*sft-4*	CBG14749	3	Inf	Unambiguous reciprocal BLAST	Supported by synteny	Confirmed by Treefam	
WBGene00006826	*unc-97*	CBG14705	3	Inf	Unambiguous reciprocal BLAST	Supported by synteny	Confirmed by Treefam	
WBGene00021060	*dct-11*	CBG14761	3	100.8239087	Unambiguous reciprocal BLAST	Supported by synteny	Confirmed by Treefam	
WBGene00017716	*F22F4.1*	CBG14814	3	Inf	Unambiguous reciprocal BLAST	Supported by synteny	Not confirmed by Treefam	
WBGene00004219	*ptr-4*	CBG14821	3	Inf	Unambiguous reciprocal BLAST	Supported by synteny	Confirmed by Treefam	
WBGene00019620	*fah-1*	CBG05065	3	Inf	Unambiguous reciprocal BLAST	Supported by synteny	Confirmed by Treefam	
WBGene00003053	*lmp-1*	CBG05052	3	115	Unambiguous reciprocal BLAST	Supported by synteny	Confirmed by Treefam	
WBGene00015800	*C15H9.4*	CBG14831	3	Inf	Unambiguous reciprocal BLAST	Supported by synteny	Confirmed by Treefam	
WBGene00000991	*dhs-28*	CBG10899	3	Inf	Unambiguous reciprocal BLAST	Supported by synteny	Confirmed by Treefam	
WBGene00016913	*lam-2*	CBG10982	3	Inf	Unambiguous reciprocal BLAST	Supported by synteny	Confirmed by Treefam	
WBGene00004949	*sox-2*	CBG16696	3	129	Unambiguous reciprocal BLAST	Supported by synteny	Confirmed by Treefam	
WBGene00017814	*F26A10.2*	CBG16748	3	Inf	Unambiguous reciprocal BLAST	Supported by synteny	Not confirmed by Treefam	
WBGene00000238	*bar-1*	CBG10985	3	Inf	Unambiguous reciprocal BLAST	Supported by synteny	Confirmed by Treefam	
WBGene00004774	*sem-5*	CBG16856	3	131.69897	Unambiguous reciprocal BLAST	Supported by synteny	Not confirmed by Treefam	
WBGene00001068	*dpy-6*	CBG22772	3	110.3467875	Unambiguous reciprocal BLAST	Supported by synteny	Not confirmed by Treefam	
WBGene00003981	*pes-8*	CBG16180	3	130.30103	Unambiguous reciprocal BLAST	Supported by synteny	Confirmed by Treefam	
WBGene00000161	*apa-2*	CBG16158	3	Inf	Unambiguous reciprocal BLAST	Supported by synteny	Confirmed by Treefam	
WBGene00014141	*ZK899.2*	CBG16133	3	Inf	Unambiguous reciprocal BLAST	Supported by synteny	Confirmed by Treefam	
WBGene00013870	*ZC373.5*	CBG01968	3	29.54406804	Unambiguous reciprocal BLAST	Supported by synteny	Confirmed by Treefam	
WBGene00010340	*slcf-1*	CBG04487	3	Inf	Unambiguous reciprocal BLAST	Supported by synteny	Confirmed by Treefam	
WBGene00001250	*elt-2*	CBG17257	3	141.1249387	Unambiguous reciprocal BLAST	Supported by synteny	Confirmed by Treefam	
WBGene00008736	*gnrr-6*	CBG17407	3	Inf	Unambiguous reciprocal BLAST	Supported by synteny	Confirmed by Treefam	
WBGene00012261	*lpr-3*	CBG17509	3	Inf	Unambiguous reciprocal BLAST	Supported by synteny	Confirmed by Treefam	
WBGene00012256	*lpr-5*	CBG17507	3	Inf	Unambiguous reciprocal BLAST	Supported by synteny	Confirmed by Treefam	
WBGene00000750	*col-177*	CBG17531	3	Inf	Unambiguous reciprocal BLAST	Supported by synteny	Not confirmed by Treefam	
WBGene00018957	*F56C11.5*	CBG22009	3	134.756962	Unambiguous reciprocal BLAST	Supported by synteny	Confirmed by Treefam	
WBGene00022373	*Y92H12BR.8*	CBG15094	3	Inf	Unambiguous reciprocal BLAST	Not supported by synteny	Confirmed by Treefam	
WBGene00000275	*bub-1*	CBG09940	3	Inf	Unambiguous reciprocal BLAST	Not supported by synteny	Confirmed by Treefam	
WBGene00000496	*chs-1*	CBG11891	3	Inf	Unambiguous reciprocal BLAST	Supported by synteny	Confirmed by Treefam	
WBGene00004305	*ran-4*	CBG12512	3	80.07918125	Unambiguous reciprocal BLAST	Supported by synteny	Confirmed by Treefam	
WBGene00004408	*rla-0*	CBG02260	3	Inf	Unambiguous reciprocal BLAST	Supported by synteny	Confirmed by Treefam	
WBGene00001228	*eif-3.E*	CBG12457	3	Inf	Unambiguous reciprocal BLAST	Supported by synteny	Confirmed by Treefam	
WBGene00021329	*Y34D9A.3*	CBG22033	3	33.30103	Unambiguous reciprocal BLAST	Supported by synteny	Not confirmed by Treefam	
WBGene00016356	*C33F10.8*	CBG20764	3	102.0791812	Unambiguous reciprocal BLAST	Not supported by synteny	Not confirmed by Treefam	
WBGene00019022	*F58A6.1*	CBG02334	3	Inf	Unambiguous reciprocal BLAST	Supported by synteny	Confirmed by Treefam	
WBGene00019061	*F58F12.1*	CBG02643	3	47.52287875	Unambiguous reciprocal BLAST	Supported by synteny	Confirmed by Treefam	

WBGene00018395	*F43E2.7*	CBG13150	3	169.1139434	Unambiguous reciprocal BLAST	Supported by synteny	Confirmed by Treefam	
WBGene00007561	*C14A4.11*	CBG03050	3	109.39794	Unambiguous reciprocal BLAST	Supported by synteny	Confirmed by Treefam	
WBGene00004454	*rpl-41*	CBG02971	3	55.69897	Unambiguous reciprocal BLAST	Supported by synteny	Confirmed by Treefam	
WBGene00010809	*M01F1.3*	CBG09779	3	Inf	Unambiguous reciprocal BLAST	Supported by synteny	Not confirmed by Treefam	
WBGene00006946	*wars-2*	CBG21169	3	Inf	Unambiguous reciprocal BLAST	Supported by synteny	Confirmed by Treefam	
WBGene00000229	*atp-2*	CBG21173	3	Inf	Unambiguous reciprocal BLAST	Supported by synteny	Confirmed by Treefam	
WBGene00020441	*T12A2.7*	CBG17487	3	150.2710668	Unambiguous reciprocal BLAST	Supported by synteny	Not confirmed by Treefam	
WBGene00015104	*B0280.9*	CBG16596	3	Inf	Unambiguous reciprocal BLAST	Supported by synteny	Confirmed by Treefam	
WBGene00015160	*B0361.6*	CBG24142	3	Inf	Unambiguous reciprocal BLAST	Supported by synteny	Confirmed by Treefam	
WBGene00007000	*tufm-1*	CBG00491	3	Inf	Unambiguous reciprocal BLAST	Not supported by synteny	Confirmed by Treefam	
WBGene00004427	*rpl-15*	CBG21258	3	131.09691	Unambiguous reciprocal BLAST	Supported by synteny	Confirmed by Treefam	
WBGene00005662	*sars-2*	CBG01669	3	Inf	Unambiguous reciprocal BLAST	Not supported by synteny	Confirmed by Treefam	
WBGene00019162	*eif-1.A*	CBG11654	3	111.4983106	Unambiguous reciprocal BLAST	Supported by synteny	Confirmed by Treefam	
WBGene00009006	*F21D5.1*	CBG16451	3	Inf	Unambiguous reciprocal BLAST	Not supported by synteny	Confirmed by Treefam	
WBGene00003066	*lpl-1*	CBG21764	3	50	Unambiguous reciprocal BLAST	Supported by synteny	Not confirmed by Treefam	
WBGene00007108	*gspd-1*	CBG06072	3	Inf	Unambiguous reciprocal BLAST	Supported by synteny	Confirmed by Treefam	
WBGene00007110	*B0035.11*	CBG06074	3	Inf	Unambiguous reciprocal BLAST	Not supported by synteny	Confirmed by Treefam	
WBGene00007111	*B0035.12*	CBG06075	3	Inf	Unambiguous reciprocal BLAST	Supported by synteny	Confirmed by Treefam	
WBGene00013109	*Y51H4A.15*	CBG13670	3	77.30103	Unambiguous reciprocal BLAST	Not supported by synteny	Confirmed by Treefam	
WBGene00013998	*ZK550.4*	CBG00367	3	Inf	Unambiguous reciprocal BLAST	Supported by synteny	Not confirmed by Treefam	
WBGene00020269	*T05H4.6a*	CBG18934	3	Inf	Unambiguous reciprocal BLAST	Supported by synteny	Confirmed by Treefam	
WBGene00004453	*rpl-39*	CBG20586	3	29.07003787	Unambiguous reciprocal BLAST	Supported by synteny	Confirmed by Treefam	
WBGene00009223	*F28F8.5*	CBG18601	3	53.41497335	Unambiguous reciprocal BLAST	Not supported by synteny	Not confirmed by Treefam	
WBGene00008441	*E01B7.1*	CBG05586	3	142.6320232	Unambiguous reciprocal BLAST	Not supported by synteny	Not confirmed by Treefam	
WBGene00007938	*C34F6.1*	CBG17404	3	Inf	Unambiguous reciprocal BLAST	Supported by synteny	Confirmed by Treefam	
WBGene00004746	*sdc-2*	CBG17307	3	157.853872	Unambiguous reciprocal BLAST	Supported by synteny	Confirmed by Treefam	
WBGene00001981	*hnd-1*	CBG15519	3	20.69897	Unambiguous reciprocal BLAST	Not supported by synteny	Not confirmed by Treefam	
WBGene00009725	*F45E6.1*	CBG00119	3	53.462398	Unambiguous reciprocal BLAST	Supported by synteny	Not confirmed by Treefam	
WBGene00007287	*gck-4*	CBG07389	3	Inf	Unambiguous reciprocal BLAST	Supported by synteny	Confirmed by Treefam	
WBGene00007848	*C31E10.7*	CBG07320	3	53.17609126	Unambiguous reciprocal BLAST	Supported by synteny	Confirmed by Treefam	
WBGene00010993	*R03E1.2*	CBG16307	3	Inf	Unambiguous reciprocal BLAST	Not supported by synteny	Confirmed by Treefam	
WBGene00007529	*C11H1.3*	CBG07248	3	Inf	Unambiguous reciprocal BLAST	Supported by synteny	Confirmed by Treefam	
WBGene00006743	*unc-3*	CBG07590	3	Inf	Unambiguous reciprocal BLAST	Supported by synteny	Confirmed by Treefam	
WBGene00007175	*B0395.3*	CBG16408	3	Inf	Unambiguous reciprocal BLAST	Supported by synteny	Confirmed by Treefam	
WBGene00020848	*ztf-19*	CBG16356	3	Inf	Unambiguous reciprocal BLAST	Supported by synteny	Confirmed by Treefam	
WBGene00004259	*pyr-1*	CBG00515	3	Inf	Unambiguous reciprocal BLAST	Supported by synteny	Confirmed by Treefam	
WBGene00010458	*K01C8.6*	CBG00671	3	154.2730013	Unambiguous reciprocal BLAST	Supported by synteny	Confirmed by Treefam	
WBGene00001840	*hel-1*	CBG00694	3	Inf	Unambiguous reciprocal BLAST	Supported by synteny	Confirmed by Treefam	
WBGene00004421	*rpl-10*	CBG00744	3	149.447158	Unambiguous reciprocal BLAST	Supported by synteny	Confirmed by Treefam	
WBGene00011040	*R05H5.5*	CBG00899	3	116.1962946	Unambiguous reciprocal BLAST	Not supported by synteny	Confirmed by Treefam	
WBGene00010325	*exos-3*	CBG02224	3	142.9542425	Unambiguous reciprocal BLAST	Supported by synteny	Confirmed by Treefam	
WBGene00001102	*dsh-2*	CBG02318	3	Inf	Unambiguous reciprocal BLAST	Supported by synteny	Confirmed by Treefam	
WBGene00015916	*C17G10.2*	CBG02451	3	Inf	Unambiguous reciprocal BLAST	Supported by synteny	Confirmed by Treefam	
WBGene00000252	*bli-2*	CBG02463	3	62.30103	Unambiguous reciprocal BLAST	Supported by synteny	Confirmed by Treefam	
WBGene00004478	*rps-9*	CBG02962	3	124	Unambiguous reciprocal BLAST	Supported by synteny	Confirmed by Treefam	
WBGene00000251	*bli-1*	CBG03026	3	Inf	Unambiguous reciprocal BLAST	Supported by synteny	Confirmed by Treefam	
WBGene00001580	*gex-3*	CBG03329	3	Inf	Unambiguous reciprocal BLAST	Supported by synteny	Confirmed by Treefam	
WBGene00012342	*mtr-4*	CBG03473	3	Inf	Unambiguous reciprocal BLAST	Supported by synteny	Confirmed by Treefam	
WBGene00001076	*dpy-17*	CBG03553	3	Inf	Unambiguous reciprocal BLAST	Supported by synteny	Confirmed by Treefam	
WBGene00000788	*cpz-1*	CBG04105	3	Inf	Unambiguous reciprocal BLAST	Supported by synteny	Confirmed by Treefam	
WBGene00018772	*F53G12.4*	CBG05101	3	23.17609126	Unambiguous reciprocal BLAST	Supported by synteny	Not confirmed by Treefam	
WBGene00022043	*Y65B4BR.8*	CBG05113	3	103.3222193	Unambiguous reciprocal BLAST	Supported by synteny	Confirmed by Treefam	
WBGene00001074	*dpy-13*	CBG05353	3	47.69897	Unambiguous reciprocal BLAST	Supported by synteny	Confirmed by Treefam	
WBGene00001043	*dnj-25*	CBG05578	3	Inf	Unambiguous reciprocal BLAST	Supported by synteny	Confirmed by Treefam	
WBGene00001079	*dpy-20*	CBG06089	3	Inf	Unambiguous reciprocal BLAST	Supported by synteny	Confirmed by Treefam	
WBGene00002363	*let-92*	CBG06179	3	Inf	Unambiguous reciprocal BLAST	Supported by synteny	Confirmed by Treefam	
WBGene00015075	*B0238.11*	CBG06777	3	77.56427143	Unambiguous reciprocal BLAST	Supported by synteny	Not confirmed by Treefam	
WBGene00001234	*eif-6*	CBG07937	3	173.50515	Unambiguous reciprocal BLAST	Supported by synteny	Confirmed by Treefam	
WBGene00018213	*F39H12.2*	CBG08107	3	47.15490196	Unambiguous reciprocal BLAST	Supported by synteny	Confirmed by Treefam	
WBGene00020216	*trap-2*	CBG08120	3	106.30103	Unambiguous reciprocal BLAST	Supported by synteny	Confirmed by Treefam	

WBGene00000437	*ceh-13*	CBG09160	3	77.52287875	Unambiguous reciprocal BLAST	Supported by synteny	Confirmed by Treefam	
WBGene00022577	*ZC250.3*	CBG09317	3	Inf	Unambiguous reciprocal BLAST	Supported by synteny	Confirmed by Treefam	
WBGene00017772	*clec-1*	CBG09340	3	101.154902	Unambiguous reciprocal BLAST	Supported by synteny	Confirmed by Treefam	
WBGene00010627	*K07C5.4*	CBG09590	3	Inf	Unambiguous reciprocal BLAST	Supported by synteny	Confirmed by Treefam	
WBGene00005022	*sqv-4*	CBG09668	3	Inf	Unambiguous reciprocal BLAST	Supported by synteny	Confirmed by Treefam	
WBGene00006540	*tbg-1*	CBG10035	3	Inf	Unambiguous reciprocal BLAST	Supported by synteny	Confirmed by Treefam	
WBGene00003934	*pat-10*	CBG10771	3	61.60205999	Unambiguous reciprocal BLAST	Supported by synteny	Confirmed by Treefam	
WBGene00001064	*dpy-2*	CBG11229	3	Inf	Unambiguous reciprocal BLAST	Supported by synteny	Not confirmed by Treefam	
WBGene00004919	*snr-6*	CBG11756	3	54.90308999	Unambiguous reciprocal BLAST	Supported by synteny	Confirmed by Treefam	
WBGene00013709	*csnk-1*	CBG12215	3	Inf	Unambiguous reciprocal BLAST	Supported by synteny	Confirmed by Treefam	
WBGene00007707	*C25A1.5*	CBG12227	3	Inf	Unambiguous reciprocal BLAST	Supported by synteny	Confirmed by Treefam	
WBGene00016750	*C48E7.2*	CBG12577	3	Inf	Unambiguous reciprocal BLAST	Supported by synteny	Confirmed by Treefam	
WBGene00004273	*rab-10*	CBG12631	3	54.69897	Unambiguous reciprocal BLAST	Supported by synteny	Confirmed by Treefam	
WBGene00001627	*gly-2*	CBG12645	3	Inf	Unambiguous reciprocal BLAST	Not supported by synteny	Confirmed by Treefam	
WBGene00000896	*dad-1*	CBG12659	3	70.24303805	Unambiguous reciprocal BLAST	Supported by synteny	Confirmed by Treefam	
WBGene00019323	*teg-4*	CBG12757	3	Inf	Unambiguous reciprocal BLAST	Supported by synteny	Confirmed by Treefam	
WBGene00003012	*lin-26*	CBG13032	3	Inf	Unambiguous reciprocal BLAST	Supported by synteny	Confirmed by Treefam	
WBGene00007021	*immp-1*	CBG13223	3	64.05799195	Unambiguous reciprocal BLAST	Supported by synteny	Confirmed by Treefam	
WBGene00001186	*egl-18*	CBG13548	3	52.17609126	Unambiguous reciprocal BLAST	Supported by synteny	Confirmed by Treefam	
WBGene00001066	*dpy-4*	CBG13781	3	30.45593196	Unambiguous reciprocal BLAST	Supported by synteny	Confirmed by Treefam	
WBGene00000049	*acr-10*	CBG14329	3	Inf	Unambiguous reciprocal BLAST	Supported by synteny	Confirmed by Treefam	
WBGene00018893	*tag-345*	CBG14922	3	Inf	Unambiguous reciprocal BLAST	Supported by synteny	Confirmed by Treefam	
WBGene00021644	*Y47G6A.18*	CBG14940	3	Inf	Unambiguous reciprocal BLAST	Supported by synteny	Confirmed by Treefam	
WBGene00003162	*mdh-2*	CBG15213	3	Inf	Unambiguous reciprocal BLAST	Supported by synteny	Confirmed by Treefam	
WBGene00006728	*ubq-2*	CBG15773	3	42.82390874	Unambiguous reciprocal BLAST	Supported by synteny	Confirmed by Treefam	
WBGene00002845	*let-711*	CBG16483	3	Inf	Unambiguous reciprocal BLAST	Supported by synteny	Confirmed by Treefam	
WBGene00020112	*pfd-5*	CBG16620	3	98.14612804	Unambiguous reciprocal BLAST	Supported by synteny	Confirmed by Treefam	
WBGene00001069	*dpy-7*	CBG16719	3	93.65321251	Unambiguous reciprocal BLAST	Supported by synteny	Confirmed by Treefam	
WBGene00008021	*C39B10.1*	CBG17255	3	Inf	Unambiguous reciprocal BLAST	Supported by synteny	Confirmed by Treefam	
WBGene00011460	*ttr-14*	CBG17593	3	124.30103	Unambiguous reciprocal BLAST	Supported by synteny	Confirmed by Treefam	
WBGene00021714	*cyh-1*	CBG17764	3	Inf	Unambiguous reciprocal BLAST	Supported by synteny	Confirmed by Treefam	
WBGene00016449	*C35D10.13*	CBG17949	3	41.50060235	Unambiguous reciprocal BLAST	Not supported by synteny	Confirmed by Treefam	
WBGene00004470	*rps-1*	CBG18056	3	Inf	Unambiguous reciprocal BLAST	Supported by synteny	Confirmed by Treefam	
WBGene00017830	*rpb-8*	CBG18189	3	102.4313638	Unambiguous reciprocal BLAST	Supported by synteny	Confirmed by Treefam	
WBGene00016142	*C26E6.6*	CBG18195	3	Inf	Unambiguous reciprocal BLAST	Supported by synteny	Confirmed by Treefam	
WBGene00016166	*C27F2.4*	CBG18202	3	Inf	Unambiguous reciprocal BLAST	Supported by synteny	Confirmed by Treefam	
WBGene00003017	*lin-31*	CBG18439	3	62.30103	Unambiguous reciprocal BLAST	Supported by synteny	Confirmed by Treefam	
WBGene00004038	*plc-3*	CBG18715	3	Inf	Unambiguous reciprocal BLAST	Not supported by synteny	Confirmed by Treefam	
WBGene00020275	*atp-4*	CBG18931	3	85.62324929	Unambiguous reciprocal BLAST	Supported by synteny	Not confirmed by Treefam	
WBGene00004482	*rps-13*	CBG19687	3	105.1303338	Unambiguous reciprocal BLAST	Supported by synteny	Confirmed by Treefam	
WBGene00012354	*W09C5.8*	CBG19770	3	119.4842998	Unambiguous reciprocal BLAST	Supported by synteny	Confirmed by Treefam	
WBGene00016291	*C31H1.8*	CBG19851	3	Inf	Unambiguous reciprocal BLAST	Not supported by synteny	Confirmed by Treefam	
WBGene00007623	*C16C10.2*	CBG20157	3	167.8920946	Unambiguous reciprocal BLAST	Not supported by synteny	Confirmed by Treefam	
WBGene00019630	*apc-16*	CBG21326	3	39.69019608	Unambiguous reciprocal BLAST	Supported by synteny	Not confirmed by Treefam	
WBGene00022035	*Y65B4BL.3*	CBG22667	3	124.6690068	Unambiguous reciprocal BLAST	Supported by synteny	Not confirmed by Treefam	
WBGene00005237	*srh-11*	CBG22745	3	121.1760913	Unambiguous reciprocal BLAST	Supported by synteny	Confirmed by Treefam	
WBGene00004806	*skp-1*	CBG23295	3	Inf	Unambiguous reciprocal BLAST	Supported by synteny	Confirmed by Treefam	
WBGene00020181	*T02H6.11*	CBG23568	3	89.32221929	Unambiguous reciprocal BLAST	Not supported by synteny	Confirmed by Treefam	
WBGene00001872	*him-14*	CBG24743	3	Inf	Unambiguous reciprocal BLAST	Not supported by synteny	Confirmed by Treefam	
WBGene00003229	*mex-3*	CBG05100	2	0	Possible alternative BLAST hit	Supported by synteny	Confirmed by Treefam	
WBGene00001814	*haf-4*	CBG22668	2	0	Possible alternative BLAST hit	Supported by synteny	Confirmed by Treefam	
WBGene00004217	*ptr-2*	CBG12004	2	0	Possible alternative BLAST hit	Supported by synteny	Confirmed by Treefam	
WBGene00006751	*unc-11*	CBG12008	2	0	Possible alternative BLAST hit	Supported by synteny	Confirmed by Treefam	
WBGene00000197	*aars-2*	CBG12085	2	0	Possible alternative BLAST hit	Supported by synteny	Confirmed by Treefam	
WBGene00006820	*unc-89*	CBG12070	2	0	Possible alternative BLAST hit	Supported by synteny	Confirmed by Treefam	
WBGene00015697	*C10H11.8*	CBG03961	2	0	Possible alternative BLAST hit	Supported by synteny	Confirmed by Treefam	
WBGene00000150	*apm-1*	CBG12329	2	0	Possible alternative BLAST hit	Supported by synteny	Confirmed by Treefam	
WBGene00004087	*ppk-1*	CBG12324	2	0	Possible alternative BLAST hit	Supported by synteny	Confirmed by Treefam	
WBGene00001333	*erm-1*	CBG12867	2	0	Possible alternative BLAST hit	Supported by synteny	Confirmed by Treefam	
WBGene00003792	*npp-6*	CBG23742	2	0	Possible alternative BLAST hit	Supported by synteny	Confirmed by Treefam	

WBGene00015092	*B0261.4*	CBG12849	2	0.045757491	Possible alternative BLAST hit	Supported by synteny	Confirmed by Treefam	
WBGene00000230	*atp-3*	CBG12309	2	0.301029996	Possible alternative BLAST hit	Supported by synteny	Confirmed by Treefam	
WBGene00001497	*fars-1*	CBG12564	2	0	Possible alternative BLAST hit	Supported by synteny	Confirmed by Treefam	
WBGene00016422	*noah-1*	CBG04015	2	0	Possible alternative BLAST hit	Supported by synteny	Confirmed by Treefam	
WBGene00004068	*pnk-1*	CBG12583	2	0	Possible alternative BLAST hit	Supported by synteny	Confirmed by Treefam	
WBGene00019000	*let-607*	CBG12652	2	0	Possible alternative BLAST hit	Supported by synteny	Confirmed by Treefam	
WBGene00001596	*gld-2*	CBG12609	2	0	Possible alternative BLAST hit	Supported by synteny	Confirmed by Treefam	
WBGene00000871	*cye-1*	CBG12774	2	0	Possible alternative BLAST hit	Supported by synteny	Confirmed by Treefam	
WBGene00016493	*C37A2.7*	CBG12770	2	13.35218252	Possible alternative BLAST hit	Supported by synteny	Confirmed by Treefam	
WBGene00011064	*R06C7.5*	CBG21917	2	0	Possible alternative BLAST hit	Supported by synteny	Confirmed by Treefam	
WBGene00003622	*nhr-23*	CBG21929	2	0	Possible alternative BLAST hit	Supported by synteny	Confirmed by Treefam	
WBGene00006754	*unc-15*	CBG11932	2	0	Possible alternative BLAST hit	Supported by synteny	Confirmed by Treefam	
WBGene00003777	*nmy-2*	CBG08250	2	0	Possible alternative BLAST hit	Supported by synteny	Confirmed by Treefam	
WBGene00003183	*mei-1*	CBG04086	2	0	Possible alternative BLAST hit	Supported by synteny	Confirmed by Treefam	
WBGene00003596	*ngp-1*	CBG04042	2	0	Possible alternative BLAST hit	Supported by synteny	Confirmed by Treefam	
WBGene00011631	*T08G11.4*	CBG12390	2	0	Possible alternative BLAST hit	Supported by synteny	Confirmed by Treefam	
WBGene00000209	*asg-1*	CBG12341	2	11.39794001	Possible alternative BLAST hit	Supported by synteny	Confirmed by Treefam	
WBGene00001568	*gei-11*	CBG12402	2	0	Possible alternative BLAST hit	Supported by synteny	Confirmed by Treefam	
WBGene00006577	*tlf-1*	CBG12345	2	0	Possible alternative BLAST hit	Supported by synteny	Confirmed by Treefam	
WBGene00001167	*eef-2*	CBG16945	2	0	Possible alternative BLAST hit	Supported by synteny	Confirmed by Treefam	
WBGene00009498	*tat-5*	CBG16933	2	0	Possible alternative BLAST hit	Supported by synteny	Confirmed by Treefam	
WBGene00003026	*lin-41*	CBG22026	2	0	Possible alternative BLAST hit	Supported by synteny	Confirmed by Treefam	
WBGene00004887	*smn-1*	CBG24493	2	2.602059991	Possible alternative BLAST hit	Supported by synteny	Confirmed by Treefam	
WBGene00001574	*gei-17*	CBG12444	2	0	Possible alternative BLAST hit	Supported by synteny	Confirmed by Treefam	
WBGene00009939	*ztf-11*	CBG03854	2	0	Possible alternative BLAST hit	Supported by synteny	Confirmed by Treefam	
WBGene00001582	*gfi-2*	CBG03811	2	0	Possible alternative BLAST hit	Supported by synteny	Confirmed by Treefam	
WBGene00006528	*tba-1*	CBG03797	2	0	Possible alternative BLAST hit	Supported by synteny	Confirmed by Treefam	
WBGene00007054	*scpl-1*	CBG03870	2	0	Possible alternative BLAST hit	Supported by synteny	Confirmed by Treefam	
WBGene00003847	*blmp-1*	CBG02201	2	0	Possible alternative BLAST hit	Supported by synteny	Confirmed by Treefam	
WBGene00008666	*F10G8.8*	CBG03790	2	0	Possible alternative BLAST hit	Supported by synteny	Confirmed by Treefam	
WBGene00003902	*pab-1*	CBG02207	2	0	Possible alternative BLAST hit	Supported by synteny	Confirmed by Treefam	
WBGene00015235	*cdc-26*	CBG12458	2	2	Possible alternative BLAST hit	Supported by synteny	Confirmed by Treefam	
WBGene00004190	*pars-2*	CBG23667	2	19.69897	Possible alternative BLAST hit	Supported by synteny	Confirmed by Treefam	
WBGene00010419	*H28O16.1*	CBG08717	2	0	Possible alternative BLAST hit	Supported by synteny	Confirmed by Treefam	
WBGene00006529	*tba-2*	CBG07939	2	0	Possible alternative BLAST hit	Supported by synteny	Confirmed by Treefam	
WBGene00012386	*agef-1*	CBG19779	2	0	Possible alternative BLAST hit	Supported by synteny	Confirmed by Treefam	
WBGene00013676	*ekl-4*	CBG08755	2	0	Possible alternative BLAST hit	Supported by synteny	Confirmed by Treefam	
WBGene00009284	*F31C3.2*	CBG19735	2	0	Possible alternative BLAST hit	Supported by synteny	Confirmed by Treefam	
WBGene00022042	*Y65B4BR.5*	CBG05114	2	0.176091259	Possible alternative BLAST hit	Supported by synteny	Confirmed by Treefam	
WBGene00003836	*nxt-1*	CBG04218	2	5.756961951	Possible alternative BLAST hit	Supported by synteny	Confirmed by Treefam	
WBGene00001340	*etr-1*	CBG06972	2	0	Possible alternative BLAST hit	Supported by synteny	Confirmed by Treefam	
WBGene00004504	*rpt-4*	CBG07004	2	0	Possible alternative BLAST hit	Supported by synteny	Confirmed by Treefam	
WBGene00021097	*cdc-37*	CBG07091	2	0	Possible alternative BLAST hit	Supported by synteny	Confirmed by Treefam	
WBGene00003825	*ntl-2*	CBG03676	2	0	Possible alternative BLAST hit	Supported by synteny	Confirmed by Treefam	
WBGene00022765	*ZK546.14*	CBG02284	2	0	Possible alternative BLAST hit	Supported by synteny	Confirmed by Treefam	
WBGene00006997	*zyg-12*	CBG02287	2	0	Possible alternative BLAST hit	Supported by synteny	Confirmed by Treefam	
WBGene00020868	*eif-1*	CBG02293	2	0	Possible alternative BLAST hit	Supported by synteny	Confirmed by Treefam	
WBGene00003393	*mog-5*	CBG02387	2	0	Possible alternative BLAST hit	Supported by synteny	Confirmed by Treefam	
WBGene00022781	*pmt-1*	CBG02363	2	0	Possible alternative BLAST hit	Supported by synteny	Confirmed by Treefam	
WBGene00000548	*clr-1*	CBG02405	2	0	Possible alternative BLAST hit	Supported by synteny	Confirmed by Treefam	
WBGene00004267	*rab-3*	CBG02488	2	0.397940009	Possible alternative BLAST hit	Supported by synteny	Confirmed by Treefam	
WBGene00002094	*ins-11*	CBG02440	2	11	Possible alternative BLAST hit	Supported by synteny	Confirmed by Treefam	
WBGene00019608	*ani-2*	CBG02638	2	0	Possible alternative BLAST hit	Supported by synteny	Confirmed by Treefam	
WBGene00001194	*egl-27*	CBG11165	2	0	Possible alternative BLAST hit	Supported by synteny	Confirmed by Treefam	
WBGene00006831	*unc-104*	CBG11201	2	0	Possible alternative BLAST hit	Supported by synteny	Confirmed by Treefam	
WBGene00002238	*kars-1*	CBG24742	2	0	Possible alternative BLAST hit	Supported by synteny	Confirmed by Treefam	
WBGene00002008	*hsp-4*	CBG13144	2	0	Possible alternative BLAST hit	Supported by synteny	Confirmed by Treefam	
WBGene00019940	*npp-21*	CBG13007	2	0	Possible alternative BLAST hit	Supported by synteny	Confirmed by Treefam	
WBGene00014051	*tag-341*	CBG03288	2	0	Possible alternative BLAST hit	Supported by synteny	Confirmed by Treefam	
WBGene00011510	*T05H10.6*	CBG13339	2	0	Possible alternative BLAST hit	Supported by synteny	Confirmed by Treefam	

WBGene00003730	*nhx-2*	CBG13035	2	0	Possible alternative BLAST hit	Supported by synteny	Confirmed by Treefam	
WBGene00002066	*ifg-1*	CBG00709	2	0	Possible alternative BLAST hit	Supported by synteny	Confirmed by Treefam	
WBGene00003241	*mig-5*	CBG00722	2	0	Possible alternative BLAST hit	Supported by synteny	Confirmed by Treefam	
WBGene00008053	*cdc-48.2*	CBG00746	2	0	Possible alternative BLAST hit	Supported by synteny	Confirmed by Treefam	
WBGene00002065	*iff-2*	CBG00567	2	2.301029996	Possible alternative BLAST hit	Supported by synteny	Confirmed by Treefam	
WBGene00010042	*bcs-1*	CBG00560	2	0	Possible alternative BLAST hit	Supported by synteny	Confirmed by Treefam	
WBGene00003196	*mel-11*	CBG00870	2	0	Possible alternative BLAST hit	Supported by synteny	Confirmed by Treefam	
WBGene00011674	*cyp-13A8*	CBG01028	2	0	Possible alternative BLAST hit	Supported by synteny	Confirmed by Treefam	
WBGene00000796	*crn-3*	CBG03060	2	0	Possible alternative BLAST hit	Supported by synteny	Confirmed by Treefam	
WBGene00007352	*cdc-48.1*	CBG03070	2	0	Possible alternative BLAST hit	Supported by synteny	Confirmed by Treefam	
WBGene00004134	*pqn-47*	CBG03089	2	0	Possible alternative BLAST hit	Supported by synteny	Confirmed by Treefam	
WBGene00002297	*ect-2*	CBG03012	2	0	Possible alternative BLAST hit	Supported by synteny	Confirmed by Treefam	
WBGene00010051	*F54D5.5*	CBG02859	2	0	Possible alternative BLAST hit	Supported by synteny	Confirmed by Treefam	
WBGene00006617	*tars-1*	CBG03144	2	0	Possible alternative BLAST hit	Supported by synteny	Confirmed by Treefam	
WBGene00007028	*trr-1*	CBG02835	2	0	Possible alternative BLAST hit	Supported by synteny	Confirmed by Treefam	
WBGene00012469	*Y17G7B.18*	CBG21009	2	0	Possible alternative BLAST hit	Supported by synteny	Confirmed by Treefam	
WBGene00013217	*ddl-3*	CBG11100	2	0	Possible alternative BLAST hit	Supported by synteny	Confirmed by Treefam	
WBGene00002996	*lin-7*	CBG11102	2	2.301029996	Possible alternative BLAST hit	Supported by synteny	Confirmed by Treefam	
WBGene00006787	*unc-52*	CBG11064	2	0	Possible alternative BLAST hit	Supported by synteny	Confirmed by Treefam	
WBGene00018794	*F54C4.3*	CBG00463	2	0	Possible alternative BLAST hit	Supported by synteny	Confirmed by Treefam	
WBGene00019212	*H19M22.3*	CBG09830	2	0	Possible alternative BLAST hit	Supported by synteny	Confirmed by Treefam	
WBGene00002915	*let-805*	CBG09834	2	0	Possible alternative BLAST hit	Supported by synteny	Confirmed by Treefam	
WBGene00004414	*rpl-3*	CBG03612	2	0	Possible alternative BLAST hit	Supported by synteny	Confirmed by Treefam	
WBGene00004765	*sel-8*	CBG09768	2	0.204119983	Possible alternative BLAST hit	Supported by synteny	Confirmed by Treefam	
WBGene00006537	*tbb-2*	CBG20196	2	0	Possible alternative BLAST hit	Supported by synteny	Confirmed by Treefam	
WBGene00007135	*cdtl-7*	CBG18089	2	0	Possible alternative BLAST hit	Supported by synteny	Confirmed by Treefam	
WBGene00003401	*mpk-1*	CBG21247	2	0	Possible alternative BLAST hit	Supported by synteny	Confirmed by Treefam	
WBGene00000380	*cct-5*	CBG18052	2	0	Possible alternative BLAST hit	Supported by synteny	Confirmed by Treefam	
WBGene00003915	*pan-1*	CBG18042	2	0	Possible alternative BLAST hit	Supported by synteny	Confirmed by Treefam	
WBGene00011409	*T04A8.7*	CBG18010	2	0	Possible alternative BLAST hit	Supported by synteny	Confirmed by Treefam	
WBGene00016140	*rpb-2*	CBG18197	2	0	Possible alternative BLAST hit	Supported by synteny	Confirmed by Treefam	
WBGene00004679	*rars-1*	CBG18188	2	0	Possible alternative BLAST hit	Supported by synteny	Confirmed by Treefam	
WBGene00020097	*larp-1*	CBG16526	2	0	Possible alternative BLAST hit	Supported by synteny	Confirmed by Treefam	
WBGene00001744	*gars-1*	CBG21320	2	0	Possible alternative BLAST hit	Supported by synteny	Confirmed by Treefam	
WBGene00018967	*F56D2.6*	CBG08977	2	0	Possible alternative BLAST hit	Supported by synteny	Confirmed by Treefam	
WBGene00017300	*F09F7.3*	CBG24045	2	0	Possible alternative BLAST hit	Supported by synteny	Confirmed by Treefam	
WBGene00006590	*toc-1*	CBG21191	2	0	Possible alternative BLAST hit	Supported by synteny	Confirmed by Treefam	
WBGene00000874	*cyk-3*	CBG09021	2	0	Possible alternative BLAST hit	Supported by synteny	Confirmed by Treefam	
WBGene00007016	*mdt-15*	CBG09077	2	0	Possible alternative BLAST hit	Supported by synteny	Confirmed by Treefam	
WBGene00006727	*ubq-1*	CBG09037	2	0	Possible alternative BLAST hit	Supported by synteny	Confirmed by Treefam	
WBGene00000381	*cct-6*	CBG09073	2	0	Possible alternative BLAST hit	Supported by synteny	Confirmed by Treefam	
WBGene00003918	*par-3*	CBG08987	2	0	Possible alternative BLAST hit	Supported by synteny	Confirmed by Treefam	
WBGene00021534	*mvk-1*	CBG21109	2	0	Possible alternative BLAST hit	Supported by synteny	Confirmed by Treefam	
WBGene00002201	*kin-18*	CBG21107	2	0	Possible alternative BLAST hit	Supported by synteny	Confirmed by Treefam	
WBGene00003796	*npp-10*	CBG09022	2	0	Possible alternative BLAST hit	Supported by synteny	Confirmed by Treefam	
WBGene00001166	*eftu-2*	CBG09023	2	0	Possible alternative BLAST hit	Supported by synteny	Confirmed by Treefam	
WBGene00017166	*aldo-2*	CBG09060	2	0	Possible alternative BLAST hit	Supported by synteny	Confirmed by Treefam	
WBGene00016977	*C56G2.1*	CBG09108	2	0	Possible alternative BLAST hit	Supported by synteny	Confirmed by Treefam	
WBGene00002891	*let-767*	CBG19695	2	0	Possible alternative BLAST hit	Supported by synteny	Confirmed by Treefam	
WBGene00004459	*rpn-2*	CBG24264	2	0	Possible alternative BLAST hit	Supported by synteny	Confirmed by Treefam	
WBGene00006463	*nduf-2.2*	CBG19683	2	0	Possible alternative BLAST hit	Supported by synteny	Confirmed by Treefam	
WBGene00003214	*mel-32*	CBG19673	2	0	Possible alternative BLAST hit	Supported by synteny	Confirmed by Treefam	
WBGene00004503	*rpt-3*	CBG09101	2	0	Possible alternative BLAST hit	Supported by synteny	Confirmed by Treefam	
WBGene00004462	*rpn-6.1*	CBG16487	2	0	Possible alternative BLAST hit	Supported by synteny	Confirmed by Treefam	
WBGene00003819	*let-765*	CBG15212	2	0	Possible alternative BLAST hit	Supported by synteny	Confirmed by Treefam	
WBGene00000872	*cyk-1*	CBG16504	2	0	Possible alternative BLAST hit	Supported by synteny	Confirmed by Treefam	
WBGene00015159	*psd-1*	CBG24141	2	0	Possible alternative BLAST hit	Supported by synteny	Confirmed by Treefam	
WBGene00001503	*fum-1*	CBG09180	2	0	Possible alternative BLAST hit	Supported by synteny	Confirmed by Treefam	
WBGene00004189	*pars-1*	CBG24153	2	0	Possible alternative BLAST hit	Supported by synteny	Confirmed by Treefam	
WBGene00015579	*C07H6.2*	CBG09169	2	14.04575749	Possible alternative BLAST hit	Supported by synteny	Confirmed by Treefam	

WBGene00003497	*mup-4*	CBG09178	2	0	Possible alternative BLAST hit	Supported by synteny	Confirmed by Treefam	
WBGene00000041	*aco-2*	CBG22943	2	0	Possible alternative BLAST hit	Supported by synteny	Confirmed by Treefam	
WBGene00006818	*unc-86*	CBG16662	2	0	Possible alternative BLAST hit	Supported by synteny	Confirmed by Treefam	
WBGene00004042	*plk-1*	CBG09233	2	0	Possible alternative BLAST hit	Supported by synteny	Confirmed by Treefam	
WBGene00007008	*rfp-1*	CBG18162	2	0	Possible alternative BLAST hit	Supported by synteny	Confirmed by Treefam	
WBGene00001028	*dnj-10*	CBG22923	2	0	Possible alternative BLAST hit	Supported by synteny	Confirmed by Treefam	
WBGene00002998	*lin-9*	CBG06880	2	0	Possible alternative BLAST hit	Supported by synteny	Confirmed by Treefam	
WBGene00001609	*glp-1*	CBG06809	2	0	Possible alternative BLAST hit	Supported by synteny	Confirmed by Treefam	
WBGene00006768	*unc-32*	CBG06881	2	0	Possible alternative BLAST hit	Supported by synteny	Confirmed by Treefam	
WBGene00006579	*tlk-1*	CBG10016	2	0	Possible alternative BLAST hit	Supported by synteny	Confirmed by Treefam	
WBGene00001263	*emb-9*	CBG10116	2	0	Possible alternative BLAST hit	Supported by synteny	Confirmed by Treefam	
WBGene00003389	*mog-1*	CBG09937	2	0	Possible alternative BLAST hit	Supported by synteny	Confirmed by Treefam	
WBGene00002064	*iff-1*	CBG10008	2	0.079181246	Possible alternative BLAST hit	Supported by synteny	Confirmed by Treefam	
WBGene00003158	*mcm-6*	CBG09994	2	0	Possible alternative BLAST hit	Supported by synteny	Confirmed by Treefam	
WBGene00003482	*mua-3*	CBG09798	2	0	Possible alternative BLAST hit	Supported by synteny	Confirmed by Treefam	
WBGene00000366	*cbp-1*	CBG09974	2	0	Possible alternative BLAST hit	Supported by synteny	Confirmed by Treefam	
WBGene00009654	*F43D9.3*	CBG18370	2	0	Possible alternative BLAST hit	Supported by synteny	Confirmed by Treefam	
WBGene00000836	*cul-1*	CBG18382	2	0	Possible alternative BLAST hit	Supported by synteny	Confirmed by Treefam	
WBGene00006395	*taf-11.3*	CBG18373	2	15.84509804	Possible alternative BLAST hit	Supported by synteny	Confirmed by Treefam	
WBGene00012972	*rsa-2*	CBG13166	2	0	Possible alternative BLAST hit	Supported by synteny	Confirmed by Treefam	
WBGene00004873	*smc-3*	CBG13206	2	0	Possible alternative BLAST hit	Supported by synteny	Confirmed by Treefam	
WBGene00012936	*Y47D3A.29*	CBG13184	2	0	Possible alternative BLAST hit	Supported by synteny	Confirmed by Treefam	
WBGene00010923	*rle-1*	CBG18273	2	0	Possible alternative BLAST hit	Supported by synteny	Confirmed by Treefam	
WBGene00004736	*sca-1*	CBG18305	2	0	Possible alternative BLAST hit	Supported by synteny	Confirmed by Treefam	
WBGene00006536	*tbb-1*	CBG18319	2	0	Possible alternative BLAST hit	Supported by synteny	Confirmed by Treefam	
WBGene00004506	*rpt-6*	CBG11771	2	0	Possible alternative BLAST hit	Supported by synteny	Confirmed by Treefam	
WBGene00006439	*ant-1.1*	CBG21201	2	0	Possible alternative BLAST hit	Supported by synteny	Confirmed by Treefam	
WBGene00003048	*lit-1*	CBG18286	2	0	Possible alternative BLAST hit	Supported by synteny	Confirmed by Treefam	
WBGene00010665	*K08E3.5*	CBG18265	2	0	Possible alternative BLAST hit	Supported by synteny	Confirmed by Treefam	
WBGene00022164	*Y71H2AL.1*	CBG09836	2	0	Possible alternative BLAST hit	Supported by synteny	Confirmed by Treefam	
WBGene00000160	*apb-1*	CBG09822	2	0	Possible alternative BLAST hit	Supported by synteny	Confirmed by Treefam	
WBGene00022048	*fln-1*	CBG10541	2	0	Possible alternative BLAST hit	Supported by synteny	Confirmed by Treefam	
WBGene00022049	*fln-1*	CBG10542	2	0	Possible alternative BLAST hit	Supported by synteny	Confirmed by Treefam	
WBGene00017926	*F29C4.2*	CBG01648	2	16.52287875	Possible alternative BLAST hit	Supported by synteny	Confirmed by Treefam	
WBGene00001999	*hrp-1*	CBG05352	2	0	Possible alternative BLAST hit	Supported by synteny	Confirmed by Treefam	
WBGene00017121	*cyc-2.1*	CBG01744	2	11.30103	Possible alternative BLAST hit	Supported by synteny	Confirmed by Treefam	
WBGene00019953	*wapl-1*	CBG01692	2	0	Possible alternative BLAST hit	Supported by synteny	Confirmed by Treefam	
WBGene00020679	*T22B11.5*	CBG01737	2	0	Possible alternative BLAST hit	Supported by synteny	Confirmed by Treefam	
WBGene00001093	*drp-1*	CBG19923	2	0	Possible alternative BLAST hit	Supported by synteny	Confirmed by Treefam	
WBGene00006974	*zen-4*	CBG05519	2	0	Possible alternative BLAST hit	Supported by synteny	Confirmed by Treefam	
WBGene00002245	*lag-1*	CBG05522	2	0	Possible alternative BLAST hit	Supported by synteny	Confirmed by Treefam	
WBGene00001241	*elo-3*	CBG05815	2	0	Possible alternative BLAST hit	Supported by synteny	Confirmed by Treefam	
WBGene00001187	*egl-19*	CBG05858	2	0	Possible alternative BLAST hit	Supported by synteny	Confirmed by Treefam	
WBGene00003914	*pam-1*	CBG05905	2	0	Possible alternative BLAST hit	Supported by synteny	Confirmed by Treefam	
WBGene00017641	*csr-1*	CBG17720	2	0	Possible alternative BLAST hit	Supported by synteny	Confirmed by Treefam	
WBGene00000994	*dic-1*	CBG19655	2	0	Possible alternative BLAST hit	Supported by synteny	Confirmed by Treefam	
WBGene00002046	*icl-1*	CBG21766	2	11.75696195	Possible alternative BLAST hit	Supported by synteny	Confirmed by Treefam	
WBGene00003787	*npp-1*	CBG03470	2	0	Possible alternative BLAST hit	Supported by synteny	Confirmed by Treefam	
WBGene00006699	*uba-1*	CBG03440	2	0	Possible alternative BLAST hit	Supported by synteny	Confirmed by Treefam	
WBGene00001249	*elt-1*	CBG20056	2	0.698970004	Possible alternative BLAST hit	Supported by synteny	Confirmed by Treefam	
WBGene00008149	*pyp-1*	CBG03439	2	0	Possible alternative BLAST hit	Supported by synteny	Confirmed by Treefam	
WBGene00011775	*T14G10.5*	CBG03408	2	0	Possible alternative BLAST hit	Supported by synteny	Confirmed by Treefam	
WBGene00004297	*rad-51*	CBG04405	2	0	Possible alternative BLAST hit	Supported by synteny	Confirmed by Treefam	
WBGene00001328	*epi-1*	CBG04423	2	0	Possible alternative BLAST hit	Supported by synteny	Confirmed by Treefam	
WBGene00002001	*hars-1*	CBG17616	2	0	Possible alternative BLAST hit	Supported by synteny	Confirmed by Treefam	
WBGene00011480	*T05E11.3*	CBG06014	2	0	Possible alternative BLAST hit	Supported by synteny	Confirmed by Treefam	
WBGene00006490	*tag-144*	CBG05992	2	0	Possible alternative BLAST hit	Supported by synteny	Confirmed by Treefam	
WBGene00008505	*F01G4.6*	CBG06020	2	0	Possible alternative BLAST hit	Supported by synteny	Confirmed by Treefam	
WBGene00001017	*dnc-1*	CBG05960	2	0	Possible alternative BLAST hit	Supported by synteny	Confirmed by Treefam	
WBGene00007444	*C08F8.2*	CBG06022	2	0	Possible alternative BLAST hit	Supported by synteny	Confirmed by Treefam	

WBGene00003415	*mars-1*	CBG06108	2	0	Possible alternative BLAST hit	Supported by synteny	Confirmed by Treefam	
WBGene00014087	*ZK809.5*	CBG06100	2	0	Possible alternative BLAST hit	Supported by synteny	Confirmed by Treefam	
WBGene00002827	*let-653*	CBG06185	2	0	Possible alternative BLAST hit	Supported by synteny	Confirmed by Treefam	
WBGene00006759	*unc-22*	CBG06205	2	0	Possible alternative BLAST hit	Supported by synteny	Confirmed by Treefam	
WBGene00006733	*ufd-1*	CBG03313	2	0	Possible alternative BLAST hit	Supported by synteny	Confirmed by Treefam	
WBGene00003037	*lin-54*	CBG01805	2	0	Possible alternative BLAST hit	Supported by synteny	Confirmed by Treefam	
WBGene00004410	*rla-2*	CBG01783	2	0	Possible alternative BLAST hit	Supported by synteny	Confirmed by Treefam	
WBGene00001007	*dli-1*	CBG01826	2	0	Possible alternative BLAST hit	Supported by synteny	Confirmed by Treefam	
WBGene00012769	*Y41E3.11*	CBG23721	2	0	Possible alternative BLAST hit	Supported by synteny	Confirmed by Treefam	
WBGene00002005	*hsp-1*	CBG00457	2	0	Possible alternative BLAST hit	Supported by synteny	Confirmed by Treefam	
WBGene00001177	*egl-8*	CBG06373	2	0	Possible alternative BLAST hit	Supported by synteny	Confirmed by Treefam	
WBGene00020391	*cct-7*	CBG21817	2	0	Possible alternative BLAST hit	Supported by synteny	Confirmed by Treefam	
WBGene00021562	*nuo-5*	CBG06645	2	0	Possible alternative BLAST hit	Supported by synteny	Confirmed by Treefam	
WBGene00004422	*rpl-11.1*	CBG01314	2	8	Possible alternative BLAST hit	Supported by synteny	Confirmed by Treefam	
WBGene00019275	*H43I07.2*	CBG01436	2	0	Possible alternative BLAST hit	Supported by synteny	Confirmed by Treefam	
WBGene00002010	*hsp-6*	CBG08827	2	0	Possible alternative BLAST hit	Supported by synteny	Confirmed by Treefam	
WBGene00006392	*taf-10*	CBG08794	2	2.15490196	Possible alternative BLAST hit	Supported by synteny	Confirmed by Treefam	
WBGene00016509	*C37H5.6*	CBG08829	2	0	Possible alternative BLAST hit	Supported by synteny	Confirmed by Treefam	
WBGene00019762	*M03F8.3*	CBG09283	2	0	Possible alternative BLAST hit	Supported by synteny	Confirmed by Treefam	
WBGene00002148	*gon-14*	CBG18977	2	0	Possible alternative BLAST hit	Supported by synteny	Confirmed by Treefam	
WBGene00018488	*acs-1*	CBG18959	2	0	Possible alternative BLAST hit	Supported by synteny	Confirmed by Treefam	
WBGene00006803	*unc-70*	CBG19038	2	0	Possible alternative BLAST hit	Supported by synteny	Confirmed by Treefam	
WBGene00016498	*mig-6*	CBG20566	2	0	Possible alternative BLAST hit	Supported by synteny	Confirmed by Treefam	
WBGene00016496	*C37C3.2*	CBG20562	2	0	Possible alternative BLAST hit	Supported by synteny	Confirmed by Treefam	
WBGene00002632	*let-413*	CBG08615	2	0	Possible alternative BLAST hit	Supported by synteny	Confirmed by Treefam	
WBGene00006760	*unc-23*	CBG19136	2	0	Possible alternative BLAST hit	Supported by synteny	Confirmed by Treefam	
WBGene00015274	*ztf-12*	CBG00762	2	0	Possible alternative BLAST hit	Supported by synteny	Confirmed by Treefam	
WBGene00008239	*fshr-1*	CBG19394	2	0	Possible alternative BLAST hit	Supported by synteny	Confirmed by Treefam	
WBGene00012528	*pap-1*	CBG09607	2	0	Possible alternative BLAST hit	Supported by synteny	Confirmed by Treefam	
WBGene00001747	*gsp-1*	CBG09676	2	0	Possible alternative BLAST hit	Supported by synteny	Confirmed by Treefam	
WBGene00006481	*tag-135*	CBG09714	2	0	Possible alternative BLAST hit	Supported by synteny	Confirmed by Treefam	
WBGene00008920	*eef-1G*	CBG23066	2	0	Possible alternative BLAST hit	Supported by synteny	Confirmed by Treefam	
WBGene00007586	*ril-2*	CBG23539	2	0	Possible alternative BLAST hit	Supported by synteny	Confirmed by Treefam	
WBGene00003916	*par-1*	CBG04756	2	0	Possible alternative BLAST hit	Supported by synteny	Confirmed by Treefam	
WBGene00001834	*hda-1*	CBG04588	2	0	Possible alternative BLAST hit	Supported by synteny	Confirmed by Treefam	
WBGene00000868	*cyb-3*	CBG05553	2	0	Possible alternative BLAST hit	Supported by synteny	Confirmed by Treefam	
WBGene00013075	*Y51A2D.7*	CBG12925	2	0	Possible alternative BLAST hit	Supported by synteny	Confirmed by Treefam	
WBGene00000498	*chk-1*	CBG06670	2	0	Possible alternative BLAST hit	Supported by synteny	Confirmed by Treefam	
WBGene00002150	*irk-2*	CBG08126	2	0	Possible alternative BLAST hit	Supported by synteny	Confirmed by Treefam	
WBGene00020507	*vha-15*	CBG14055	2	0	Possible alternative BLAST hit	Supported by synteny	Confirmed by Treefam	
WBGene00004423	*rpl-11.2*	CBG14053	2	10.82390874	Possible alternative BLAST hit	Supported by synteny	Confirmed by Treefam	
WBGene00003776	*nmy-1*	CBG02025	2	0	Possible alternative BLAST hit	Supported by synteny	Confirmed by Treefam	
WBGene00006810	*unc-78*	CBG14204	2	0	Possible alternative BLAST hit	Supported by synteny	Confirmed by Treefam	
WBGene00004729	*sax-3*	CBG14221	2	0	Possible alternative BLAST hit	Supported by synteny	Confirmed by Treefam	
WBGene00018533	*F47B7.2*	CBG14281	2	0	Possible alternative BLAST hit	Supported by synteny	Confirmed by Treefam	
WBGene00003963	*pdi-2*	CBG14484	2	0	Possible alternative BLAST hit	Supported by synteny	Confirmed by Treefam	
WBGene00006921	*vha-12*	CBG14362	2	0	Possible alternative BLAST hit	Supported by synteny	Confirmed by Treefam	
WBGene00003412	*mrp-6*	CBG14361	2	0	Possible alternative BLAST hit	Supported by synteny	Confirmed by Treefam	
WBGene00001082	*dpy-23*	CBG14461	2	0	Possible alternative BLAST hit	Supported by synteny	Confirmed by Treefam	
WBGene00000149	*apl-1*	CBG14611	2	0	Possible alternative BLAST hit	Supported by synteny	Confirmed by Treefam	
WBGene00020734	*bus-8*	CBG14694	2	0	Possible alternative BLAST hit	Supported by synteny	Confirmed by Treefam	
WBGene00002190	*kin-2*	CBG14791	2	0	Possible alternative BLAST hit	Supported by synteny	Confirmed by Treefam	
WBGene00002007	*hsp-3*	CBG14829	2	0	Possible alternative BLAST hit	Supported by synteny	Confirmed by Treefam	
WBGene00003877	*pept-1*	CBG05068	2	0	Possible alternative BLAST hit	Supported by synteny	Confirmed by Treefam	
WBGene00003530	*nas-11*	CBG10873	2	0	Possible alternative BLAST hit	Supported by synteny	Confirmed by Treefam	
WBGene00000210	*asg-2*	CBG10906	2	11.47712125	Possible alternative BLAST hit	Supported by synteny	Confirmed by Treefam	
WBGene00000083	*adt-2*	CBG16735	2	0	Possible alternative BLAST hit	Supported by synteny	Confirmed by Treefam	
WBGene00015391	*sdha-1*	CBG22795	2	0	Possible alternative BLAST hit	Supported by synteny	Confirmed by Treefam	
WBGene00016006	*fln-2*	CBG16142	2	0	Possible alternative BLAST hit	Supported by synteny	Confirmed by Treefam	
WBGene00003232	*mgl-1*	CBG01949	2	0	Possible alternative BLAST hit	Supported by synteny	Confirmed by Treefam	

WBGene00003553	*nas-37*	CBG01954	2	0	Possible alternative BLAST hit	Supported by synteny	Confirmed by Treefam	
WBGene00009617	*F41E7.1*	CBG17199	2	0	Possible alternative BLAST hit	Supported by synteny	Confirmed by Treefam	
WBGene00004036	*plc-1*	CBG17587	2	0	Possible alternative BLAST hit	Supported by synteny	Confirmed by Treefam	
WBGene00004888	*smo-1*	CBG22301	2	0	Possible alternative BLAST hit	Supported by synteny	Confirmed by Treefam	
WBGene00000466	*cel-1*	CBG03700	2	0	Possible alternative BLAST hit	Supported by synteny	Confirmed by Treefam	
WBGene00000410	*cdk-9*	CBG20416	2	0	Possible alternative BLAST hit	Supported by synteny	Confirmed by Treefam	
WBGene00022743	*mlt-7*	CBG03680	2	0	Possible alternative BLAST hit	Supported by synteny	Confirmed by Treefam	
WBGene00016115	*mdt-26*	CBG02470	2	0	Possible alternative BLAST hit	Supported by synteny	Confirmed by Treefam	
WBGene00015512	*C06A8.1*	CBG12960	2	0	Possible alternative BLAST hit	Supported by synteny	Confirmed by Treefam	
WBGene00001358	*evl-20*	CBG24969	2	0	Possible alternative BLAST hit	Supported by synteny	Confirmed by Treefam	
WBGene00018239	*sec-20*	CBG15723	2	0	Possible alternative BLAST hit	Supported by synteny	Confirmed by Treefam	
WBGene00003001	*lin-12*	CBG06826	2	0	Possible alternative BLAST hit	Supported by synteny	Confirmed by Treefam	
WBGene00001155	*ech-6*	CBG10003	2	0	Possible alternative BLAST hit	Supported by synteny	Confirmed by Treefam	
WBGene00013236	*Y56A3A.18*	CBG13319	2	0	Possible alternative BLAST hit	Supported by synteny	Confirmed by Treefam	
WBGene00016384	*C33H5.18*	CBG17684	2	0	Possible alternative BLAST hit	Supported by synteny	Confirmed by Treefam	
WBGene00010047	*F54D1.6*	CBG06054	2	0	Possible alternative BLAST hit	Supported by synteny	Confirmed by Treefam	
WBGene00015461	*C05C8.2*	CBG19114	2	0	Possible alternative BLAST hit	Supported by synteny	Confirmed by Treefam	
WBGene00000098	*air-1*	CBG24785	2	0	Possible alternative BLAST hit	Supported by synteny	Confirmed by Treefam	
WBGene00000064	*act-2*	CBG23091	2	0	Possible alternative BLAST hit	Supported by synteny	Confirmed by Treefam	
WBGene00001184	*egl-15*	CBG17516	2	0	Possible alternative BLAST hit	Supported by synteny	Confirmed by Treefam	
WBGene00003003	*lin-14*	CBG17326	2	0	Possible alternative BLAST hit	Supported by synteny	Confirmed by Treefam	
WBGene00008205	*sams-1*	CBG15526	2	0	Possible alternative BLAST hit	Supported by synteny	Confirmed by Treefam	
WBGene00011938	*alh-13*	CBG00074	2	0	Possible alternative BLAST hit	Supported by synteny	Confirmed by Treefam	
WBGene00003623	*nhr-25*	CBG16911	2	0	Possible alternative BLAST hit	Supported by synteny	Confirmed by Treefam	
WBGene00000105	*alg-1*	CBG07340	2	0	Possible alternative BLAST hit	Supported by synteny	Confirmed by Treefam	
WBGene00003411	*mrp-5*	CBG07659	2	0	Possible alternative BLAST hit	Supported by synteny	Confirmed by Treefam	
WBGene00008605	*mlt-9*	CBG07630	2	0	Possible alternative BLAST hit	Supported by synteny	Confirmed by Treefam	
WBGene00001520	*gas-1*	CBG07730	2	0	Possible alternative BLAST hit	Supported by synteny	Confirmed by Treefam	
WBGene00007463	*C08H9.2*	CBG00996	2	0	Possible alternative BLAST hit	Supported by synteny	Confirmed by Treefam	
WBGene00012186	*mlt-11*	CBG05636	2	0	Possible alternative BLAST hit	Supported by synteny	Confirmed by Treefam	
WBGene00004181	*pri-2*	CBG08720	2	0	Possible alternative BLAST hit	Supported by synteny	Confirmed by Treefam	
WBGene00003795	*npp-9*	CBG09863	2	0	Possible alternative BLAST hit	Supported by synteny	Confirmed by Treefam	
WBGene00044072	*tag-246*	CBG09873	2	0	Possible alternative BLAST hit	Supported by synteny	Confirmed by Treefam	
WBGene00004027	*pie-1*	CBG11755	2	14.87506126	Possible alternative BLAST hit	Supported by synteny	Confirmed by Treefam	
WBGene00000938	*dcp-66*	CBG11888	2	0	Possible alternative BLAST hit	Supported by synteny	Confirmed by Treefam	
WBGene00000800	*cars-1*	CBG04128	1	0	Possible alternative BLAST hit	Supported by synteny	Confirmed by Treefam	
WBGene00016907	*C53H9.2*	CBG22213	1	0	Possible alternative BLAST hit	Not supported by synteny	Confirmed by Treefam	
WBGene00006805	*unc-73*	CBG12078	1	0	Possible alternative BLAST hit	Not supported by synteny	Confirmed by Treefam	
WBGene00000962	*dhc-1*	CBG12193	1	0	Possible alternative BLAST hit	Not supported by synteny	Confirmed by Treefam	
WBGene00019001	*F57B10.3*	CBG12654	1	0	Possible alternative BLAST hit	Not supported by synteny	Confirmed by Treefam	
WBGene00001214	*ego-1*	CBG00321	1	0	Possible alternative BLAST hit	Not supported by synteny	Confirmed by Treefam	
WBGene00003071	*lrp-1*	CBG11882	1	0	Possible alternative BLAST hit	Not supported by synteny	Confirmed by Treefam	
WBGene00009188	*lsy-22*	CBG00300	1	2.301029996	Possible alternative BLAST hit	Supported by synteny	Not confirmed by Treefam	
WBGene00004378	*rme-8*	CBG11852	1	0	Possible alternative BLAST hit	Supported by synteny	Not confirmed by Treefam	
WBGene00009743	*sptf-1*	CBG21350	1	0.124938737	Possible alternative BLAST hit	Supported by synteny	Not confirmed by Treefam	
WBGene00009371	*F33H2.8*	CBG19733	1	1	Possible alternative BLAST hit	Supported by synteny	Not confirmed by Treefam	
WBGene00004775	*sep-1*	CBG14956	1	0	Possible alternative BLAST hit	Supported by synteny	Not confirmed by Treefam	
WBGene00016074	*C24H12.5*	CBG07061	1	0	Possible alternative BLAST hit	Not supported by synteny	Confirmed by Treefam	
WBGene00004076	*pod-2*	CBG07699	1	0	Possible alternative BLAST hit	Not supported by synteny	Confirmed by Treefam	
WBGene00017369	*F10G7.5*	CBG13412	1	0	Possible alternative BLAST hit	Not supported by synteny	Confirmed by Treefam	
WBGene00019888	*R05F9.1*	CBG02271	1	0	Possible alternative BLAST hit	Not supported by synteny	Confirmed by Treefam	
WBGene00006923	*vhp-1*	CBG02372	1	0	Possible alternative BLAST hit	Not supported by synteny	Confirmed by Treefam	
WBGene00002053	*ifb-1*	CBG02495	1	0	Possible alternative BLAST hit	Not supported by synteny	Confirmed by Treefam	
WBGene00001072	*dpy-10*	CBG11227	1	10.30103	Possible alternative BLAST hit	Supported by synteny	Not confirmed by Treefam	
WBGene00022855	*tcer-1*	CBG11210	1	0	Possible alternative BLAST hit	Supported by synteny	Not confirmed by Treefam	
WBGene00000894	*dab-1*	CBG00708	1	0	Possible alternative BLAST hit	Not supported by synteny	Confirmed by Treefam	
WBGene00000289	*cam-1*	CBG20223	1	0	Possible alternative BLAST hit	Not supported by synteny	Confirmed by Treefam	
WBGene00007332	*C05C10.5*	CBG09062	1	0.602059991	Possible alternative BLAST hit	Not supported by synteny	Confirmed by Treefam	
WBGene00008546	*F07A11.2*	CBG02850	1	0	Possible alternative BLAST hit	Supported by synteny	Not confirmed by Treefam	
WBGene00006915	*vha-6*	CBG02894	1	0	Possible alternative BLAST hit	Not supported by synteny	Confirmed by Treefam	

WBGene00003015	*lin-29*	CBG02753	1	0	Possible alternative BLAST hit	Not supported by synteny	Confirmed by Treefam	
WBGene00002076	*imb-2*	CBG11089	1	0	Possible alternative BLAST hit	Not supported by synteny	Confirmed by Treefam	
WBGene00012179	*W01D2.1*	CBG04239	1	4	Possible alternative BLAST hit	Supported by synteny	Not confirmed by Treefam	
WBGene00012896	*Y46G5A.4*	CBG07909	1	0	Possible alternative BLAST hit	Not supported by synteny	Confirmed by Treefam	
WBGene00003134	*mat-3*	CBG12598	1	0	Possible alternative BLAST hit	Not supported by synteny	Confirmed by Treefam	
WBGene00003577	*ndg-4*	CBG18058	1	0	Possible alternative BLAST hit	Not supported by synteny	Confirmed by Treefam	
WBGene00020093	*R144.3*	CBG16523	1	1.77815125	Possible alternative BLAST hit	Supported by synteny	Not confirmed by Treefam	
WBGene00016197	*pxl-1*	CBG09041	1	0	Possible alternative BLAST hit	Not supported by synteny	Confirmed by Treefam	
WBGene00020549	*nmt-1*	CBG21106	1	0	Possible alternative BLAST hit	Not supported by synteny	Confirmed by Treefam	
WBGene00000549	*cls-2*	CBG09877	1	0	Possible alternative BLAST hit	Not supported by synteny	Confirmed by Treefam	
WBGene00002219	*klp-7*	CBG18307	1	0	Possible alternative BLAST hit	Supported by synteny	Not confirmed by Treefam	
WBGene00001077	*dpy-18*	CBG13195	1	0	Possible alternative BLAST hit	Not supported by synteny	Confirmed by Treefam	
WBGene00004161	*pqn-80*	CBG20130	1	0	Possible alternative BLAST hit	Supported by synteny	Not confirmed by Treefam	
WBGene00003406	*mrg-1*	CBG15768	1	3.096910013	Possible alternative BLAST hit	Not supported by synteny	Confirmed by Treefam	
WBGene00000834	*cua-1*	CBG21197	1	0	Possible alternative BLAST hit	Not supported by synteny	Confirmed by Treefam	
WBGene00020297	*T07A9.9*	CBG13493	1	0	Possible alternative BLAST hit	Not supported by synteny	Confirmed by Treefam	
WBGene00022310	*Y77E11A.7*	CBG13944	1	0	Possible alternative BLAST hit	Supported by synteny	Not confirmed by Treefam	
WBGene00001983	*hoe-1*	CBG01741	1	0	Possible alternative BLAST hit	Not supported by synteny	Confirmed by Treefam	
WBGene00001132	*alp-1*	CBG24239	1	0	Possible alternative BLAST hit	Not supported by synteny	Confirmed by Treefam	
WBGene00001650	*gon-1*	CBG03449	1	0	Possible alternative BLAST hit	Not supported by synteny	Confirmed by Treefam	
WBGene00001398	*fat-6*	CBG06190	1	0	Possible alternative BLAST hit	Supported by synteny	Not confirmed by Treefam	
WBGene00003230	*mex-5*	CBG12031	1	0	Possible alternative BLAST hit	Supported by synteny	Not confirmed by Treefam	
WBGene00001336	*qars-1*	CBG13773	1	0	Possible alternative BLAST hit	Not supported by synteny	Confirmed by Treefam	
WBGene00001330	*eps-8*	CBG13858	1	0	Possible alternative BLAST hit	Not supported by synteny	Confirmed by Treefam	
WBGene00021935	*Y55F3BL.1*	CBG23913	1	0	Possible alternative BLAST hit	Supported by synteny	Not confirmed by Treefam	
WBGene00022642	*lipl-5*	CBG01370	1	0	Possible alternative BLAST hit	Not supported by synteny	Confirmed by Treefam	
WBGene00006796	*unc-62*	CBG24578	1	0	Possible alternative BLAST hit	Not supported by synteny	Confirmed by Treefam	
WBGene00003515	*myo-3*	CBG23416	1	0	Possible alternative BLAST hit	Not supported by synteny	Confirmed by Treefam	
WBGene00004013	*pha-4*	CBG05577	1	0	Possible alternative BLAST hit	Not supported by synteny	Confirmed by Treefam	
WBGene00002056	*ifc-2*	CBG08136	1	0	Possible alternative BLAST hit	Not supported by synteny	Confirmed by Treefam	
WBGene00001863	*him-4*	CBG16045	1	0	Possible alternative BLAST hit	Supported by synteny	Not confirmed by Treefam	
WBGene00002261	*ldb-1*	CBG17513	1	0	Possible alternative BLAST hit	Not supported by synteny	Confirmed by Treefam	
WBGene00002073	*ima-2*	CBG18786	1	0	Possible alternative BLAST hit	Not supported by synteny	Confirmed by Treefam	
WBGene00001230	*eif-3.G*	CBG24970	1	0	Possible alternative BLAST hit	Not supported by synteny	Confirmed by Treefam	
WBGene00001561	*gei-4*	CBG15706	1	0	Possible alternative BLAST hit	Supported by synteny	Not confirmed by Treefam	
WBGene00004340	*rfc-4*	CBG24567	1	0	Possible alternative BLAST hit	Not supported by synteny	Confirmed by Treefam	
WBGene00012361	*W09D10.3*	CBG18380	1	0	Possible alternative BLAST hit	Not supported by synteny	Confirmed by Treefam	
WBGene00000067	*act-5*	CBG18256	1	0	Possible alternative BLAST hit	Not supported by synteny	Confirmed by Treefam	
WBGene00003392	*mog-4*	CBG20862	1	0	Possible alternative BLAST hit	Not supported by synteny	Confirmed by Treefam	
WBGene00001945	*his-71*	CBG16863	1	0.096910013	Possible alternative BLAST hit	Supported by synteny	Not confirmed by Treefam	
WBGene00016007	*fln-2*	CBG16145	1	0	Possible alternative BLAST hit	Supported by synteny	Not confirmed by Treefam	
WBGene00015676	*C10E2.6*	CBG16244	1	0	Possible alternative BLAST hit	Not supported by synteny	Confirmed by Treefam	
WBGene00006522	*abcx-1*	CBG02686	1	0	Possible alternative BLAST hit	Supported by synteny	Not confirmed by Treefam	
WBGene00010785	*top-2*	CBG02746	1	0	Possible alternative BLAST hit	Supported by synteny	Not confirmed by Treefam	
WBGene00006527	*tax-6*	CBG08362	1	0	Possible alternative BLAST hit	Not supported by synteny	Confirmed by Treefam	
WBGene00001946	*his-72*	CBG11770	1	0.823908741	Possible alternative BLAST hit	Supported by synteny	Not confirmed by Treefam	
WBGene00000203	*arx-5*	CBG15746	1	10.69897	Possible alternative BLAST hit	Not supported by synteny	Confirmed by Treefam	
WBGene00002083	*inf-1*	CBG16478	1	0	Possible alternative BLAST hit	Supported by synteny	Not confirmed by Treefam	
WBGene00012666	*Y39B6A.3*	CBG20529	1	12	Possible alternative BLAST hit	Not supported by synteny	Confirmed by Treefam	
WBGene00000253	*bli-3*	CBG05103	0	0	Possible alternative BLAST hit	Not supported by synteny	Not confirmed by Treefam	
WBGene00002994	*lin-5*	CBG23227	0	0	Possible alternative BLAST hit	Not supported by synteny	Not confirmed by Treefam	
WBGene00006780	*unc-44*	CBG09473	0	0	Possible alternative BLAST hit	Not supported by synteny	Not confirmed by Treefam	
WBGene00002173	*itr-1*	CBG05938	0	0	Possible alternative BLAST hit	Not supported by synteny	Not confirmed by Treefam	
WBGene00019086	*F59A6.4*	CBG20869	0	0	Possible alternative BLAST hit	Not supported by synteny	Not confirmed by Treefam	
WBGene00020279	*glb-25*	CBG24801	0	0	Possible alternative BLAST hit	Not supported by synteny	Not confirmed by Treefam	
WBGene00008266	*C53A5.6*	CBG23781	0	0.176091259	Possible alternative BLAST hit	Not supported by synteny	Not confirmed by Treefam	
WBGene00000066	*act-4*	CBG23090	0	0	Possible alternative BLAST hit	Not supported by synteny	Not confirmed by Treefam	
WBGene00000231	*atx-2*	CBG18377	0	0	Possible alternative BLAST hit	Not supported by synteny	Not confirmed by Treefam	
