# Supplementary material for: Comparative RNAi Screens in C. elegans and C. briggsae Reveal the Impact of Developmental System Drift on Gene Function
Source: PLoS Genet. 2014 Feb 6;10(2):e1004077. doi: 10.1371/journal.pgen.1004077 (PMC3916228; doi:10.1371/journal.pgen.1004077)
Supplement: Table S2 — Genes identified by eye as having a different phenotype between C. elegans and C. briggsae. The number of supporting observations in the primary screen (out of 4) and the secondary screen (out of 2) is shown. Functional annotations are from the manual annotation in Kamath et al. (PDF) [file pgen.1004077.s008.pdf]

Gene(WBID)	Gene(Common)	*C.elegans* phenotype	*C.briggsae* phenotype	Primary clone (out of 4 c	Secondary clone (out of	Functional Class	Description
WBGene00000079	*adr-1*	Ste;Lvl	Wt	4	2	RNA Synthesis	Adenosine deaminase that acts on RNA (ADAR)
WBGene00000156	*apr-1*	Unc;Bmd;Lvl	Wt	4	2	Signalling	Orthologue of APC
WBGene00000255	*bli-5*	Bli	Wt	4	2	Proteases	Kunitz family serine protease inhibitor
WBGene00000814	*csn-2*	50-80%_Emb;Gro	Wt	4	2	Degradation	Subunit of COP9 signalosome complex
WBGene00001130	*dyn-1*	100%_Emb;Unc;Mlt;Clr	Wt	4	2	Cell Architecture	Dynamin GTPase
WBGene00001345	*fos-1*	Ste;Rup;Pvl	Gro	4	2	Transcription Factor	Basic region-leucine zipper TF
WBGene00001979	*hmp-2*	100%_Emb;Unc;Dpy;Bmd	Wt	4	2	Signalling	Beta-catenin
WBGene00001980	*hmr-1*	10%_Emb;Unc;Bmd;Dpy	10%_Emb	4	2	Cell Architecture	Cadherin
WBGene00003044	*lir-1*	10%_Emb;Lvl;Unc;Mlt	Wt	4	2	Transcription Factor	LIN-26-like zinc-finger protein
WBGene00003123	*mag-1*	90%_Emb;Gro;Dpy	Gro;Dpy	4	2	RNA Synthesis	EJC member, mago orthologue
WBGene00003209	*mel-26*	100%_Emb	Wt	4	2	Degradation	Adaptor of CUL-3-containing E3 ubiquitin ligase
WBGene00003210	*mel-28*	100.00%_Emb	Gro	4	2	Cell Architecture	Required for nuclear envelope assembly
WBGene00003651	*nhr-61*	20-40%_Emb;Gro;Pch;Sma	Wt	4	2	Transcription Factor	Nuclear hormone receptor
WBGene00004198	*prx-13*	Gro	Wt	4	2	Cell Architecture	Peroxisome assembly factor
WBGene00004430	*rpl-18*	Ste;Sck	Gro	4	2	Protein Synthesis	Ribosomal protein L18
WBGene00004450	*rpl-36*	Ste;Sck	Red	4	2	Protein Synthesis	Robosomal protein L36
WBGene00004857	*sma-3*	Sma	Wt	4	2	Cell Architecture	Beta-H spectrin
WBGene00006428	*tag-49*	Ste;Sck	Wt	4	2	Neuro	Neuropeptide receptor
WBGene00006647	*tsr-1*	100%_Emb	50%_Emb	4	2	Cell Architecture	Nuclear transport receptor
WBGene00007275	*C03D6.1*	Gro	Wt	4	2	Unknown	Protein of unknown function
WBGene00008670	*F11A3.2*	20-40%_Emb;Gro	Wt	4	2	Protein Synthesis	Translation initiation factor eIF-2B subunit delta
WBGene00009264	*sac-1*	Gro	Wt	4	2	Cell Architecture	Inositol polyphosphate-5-phosphatase
WBGene00009626	*F42A8.1*	50-80%_Emb;Lva;Dpy;Bmd;	Wt	4	2	Unknown	Protein of unknown function
WBGene00010941	*M176.2*	20-40%_Emb;Gro;Clr;Thn;P	20-40%_Emb;Weak Gro	4	2	Metabolism	Putative glutathione synthetase
WBGene00012235	*W04A4.6*	90%_Emb;Gro;Unc;Bmd	Wt	4	2	Unknown	Protein of unknown function
WBGene00012803	*Y43F4B.5*	Gro;Lvl	Wt	4	2	Metabolism	Putative phosphomannomutase
WBGene00014066	*rev-1*	100%_Emb	Wt	4	2	DNA/Cell Cycle	Translesion DNA polymerase
WBGene00016020	*sptl-1*	Gro;Lvl;Unc	Unc	4	2	Metabolism	Putative subunit of serine palmitoyltransferase
WBGene00016323	*C32E8.5*	100%_Emb;1-May;Gro	20%_Emb	4	2	RNA Synthesis	Similarity to Smad nuclear-interacting protein
WBGene00016721	*C46G7.1*	Gro	Wt	4	2	Unknown	Protein of unknown function
WBGene00017358	*F10E9.7*	Ste;Gro;Sck	Wt	4	2	Unknown	Protein of unknown function
WBGene00017769	*F25B4.6*	20-40%_Emb;Gro;Lvl;Bmd;U	Wt	4	2	Metabolism	Hydroxymethylglutaryl-CoA synthase
WBGene00017853	*F27C1.3*	Gro;Stp	Wt	4	2	Unknown	Protein of unknown function
WBGene00018793	*F54C4.1*	20-40%_Emb;Gro	20-40%_Emb	4	2	Protein Synthesis	Putative mitochondrial ribosome L30
WBGene00018961	*F56D1.3*	10%_Emb;Gro	Wt	4	2	Protein Synthesis	Subinit of mitochondrial ribosome (S)
WBGene00019126	*F59E12.11*	100%_Emb	Wt	4	2	Unknown	Protein of unknown function
WBGene00019455	*K06H7.1*	100%_Emb;Adl;Lvl;Pvl	Wt	4	2	Unknown	Protein of unknown function
WBGene00021365	*smgl-2*	100%_Emb;Unc;Lvl;Dpy	Wt	4	2	NA Binding	DEAH helicase
WBGene00021465	*Y39G10AR.7*	100%_Emb;1-May;Lva;Dpy;	Wt	4	2	Unknown	Protein of unknown function
WBGene00021468	*epg-2*	100%_Emb;Lvl	Wt	4	2	Unknown	Protein of unknown function
WBGene00021626	*Y47D7A.14*	90%_Emb	Wt	4	2	Metabolism	Putative riboflavin transporter
WBGene00022027	*vps-20*	Gro;Unc;Prz	Weak Gro;Unc;Prz	4	2	Cell Architecture	VPS-20 orthologue; likely role in trafficking
WBGene00022117	*Y71F9AL.12*	20-40%_Emb;Ste	Wt	4	2	Unknown	Protein of unknown function
WBGene00000254	*bli-4*	Mlt;Dpy;Lvl	Mlt	4	1	Proteases	KEX2/subtilisin serine endoprotease
WBGene00000431	*ceh-6*	Unc;Mlt	Wt	4	1	Transcription Factor	POU family homeodomain protein
WBGene00002152	*iars-1*	50-80%_Emb;Red;Gro	Gro	4	1	Protein Synthesis	Isoleucyl-tRNA synthetase
WBGene00003904	*pabp-2*	90%_Emb;Lva;Unc	Wt	4	1	RNA Synthesis	Polyadenylate-binding protein
WBGene00003912	*pal-1*	100%_Emb	Wt	4	1	Transcription Factor	Homeodomain protein; C. elegans Caudal orthologue
WBGene00004194	*prx-5*	Gro;Clr;Thn	Wt	4	1	Cell Architecture	Receptor for peroxisomal targeting signal
WBGene00004705	*rsp-8*	Gro	Wt	4	1	RNA Synthesis	Likely splicing factor
WBGene00004786	*sex-1*	20-40%_Emb;mult;Dpy;Bmd	Wt	4	1	Transcription Factor	Nuclear hormone receptor
WBGene00004951	*spc-1*	mult;Bmd;Dpy	Wt	4	1	Cell Architecture	Alpha spectrin
WBGene00014229	*ZK1128.3*	Gro;Unc;Rup;Pvl	Wt	4	1	Unknown	Protein of unknown function
WBGene00017916	*F29A7.6*	Gro;Stp;Pch;Pvl	Weak Gro	4	1	Unknown	Protein of unknown function

WBGene00000817	*csn-5*	50-80%_Emb	Wt	3	2	Degradation	Subunit 5 of the COP9 signalosome complex
WBGene00001086	*dpy-27*	Dpy;Egl	Wt	3	2	Chromatin	SMC family member, dosage compensation
WBGene00001662	*gop-3*	100%_Emb;Gro	Gro	3	2	Metabolism	Similarity to mitochondrial SAM50
WBGene00004374	*rme-2*	Ste	Gro	3	2	Cell Architecture	LDL-receptor related
WBGene00004700	*rsp-3*	100%_Emb	50%_Emb	3	2	RNA Synthesis	Likely SR-family splice factor
WBGene00004855	*sma-1*	Gro;Bmd;Sma;Rol	Wt	3	2	Cell Architecture	Beta-H spectrin
WBGene00008166	*saps-1*	50-80%_Emb;Unc;Clr;Stp	50-80%_Emb	3	2	Unknown	Protein of unknown function
WBGene00011538	*T06E6.1*	Gro	Weak Gro	3	2	Unknown	Protein of unknown function
WBGene00015146	*abi-1*	50-80%_Emb;Bmd;Adl	Wt	3	2	Signalling	Orthologue of human Abl interactor SSH3BP1
WBGene00015298	*C01F1.3*	Lvl;Unc;Pch	Wt	3	2	Metabolism	Putative thymidine diphosphoglucose 4
WBGene00016442	*C35D10.5*	20-40%_Emb;Gro	Wt	3	2	Metabolism	Ubiquinol-cytochrome c reductase complex chaperone
WBGene00017852	*F27C1.2*	Him	Wt	3	2	Small Molecule Transport	Similarity to copper transporter
WBGene00017982	*F32D1.2*	50-80%_Emb;Gro;Stp	Gro	3	2	Metabolism	Hypersensitive to pore forming toxin
WBGene00018144	*F37C4.4*	50-80%_Emb	50%_Emb	3	2	Unknown	Protein of unknown function
WBGene00019400	*K04G7.1*	50-80%_Emb;Gro;Thn;Stp	Gro	3	2	Unknown	Protein of unknown function
WBGene00020149	*T01D1.4*	Unc;Dpy	Wt	3	2	Metabolism	Similarity to methylthiopentene dioxygenase
WBGene00020705	*T22H9.1*	Gro;Pch;Stp	Wt	3	2	Protein Synthesis	Similarity to Ribosomal RNA processing protein 36
WBGene00022201	*Y71H10B.1*	Gro;Clr;Thn;Stp	Wt	3	2	Metabolism	Purine nucleotidase
WBGene00000675	*col-101*	Gro;Clr;Sma	Wt	3	1	Collagen	Cuticle collagen
WBGene00001465	*flr-1*	Gro;Clr	Weak Gro	3	1	Small Molecule Transport	Amiloride-sensitive sodium channel
WBGene00001824	*hbl-1*	20-40%_Emb;mult;Pvl;Dpy	20-40%_Emb	3	1	Transcription Factor	C2H2 zinc finger TF related to Drosophila hunchback
WBGene00001974	*hmg-4*	50-80%_Emb;Gro;Unc;Prz;L	50-80%_Emb;Gro	3	1	Chromatin	Putative FACT complex subunit
WBGene00003159	*mcm-7*	100%_Emb	50-80%_Emb	3	1	DNA/Cell Cycle	MCM complex subunit
WBGene00004197	*prx-12*	Gro;Clr	Weak Gro;Clr	3	1	Cell Architecture	Peroxisome assembly factor
WBGene00004201	*prx-19*	Gro	Wt	3	1	Cell Architecture	Peroxisome assembly factor
WBGene00004271	*rab-7*	20-40%_Emb;Prz;Bmd;Dpy	Prz;Bmd;Dpy	3	1	Cell Architecture	Rab GTPase
WBGene00004735	*sbp-1*	6-Oct;Gro;Clr;Sck	Wt	3	1	Transcription Factor	Basic helix-loop-helix (bHLH) related to SREBPs
WBGene00006914	*vha-5*	10%_Emb;Gro;mult;Unc;Clr	10%_Emb	3	1	Small Molecule Transport	Subunit of vacuolar proton-translocating ATPase
WBGene00009259	*F29G6.3*	Gro;Clr	Wt	3	1	Unknown	Protein of unknown function
WBGene00009504	*F37B12.1*	Pvl;Rup;Egl	Wt	3	1	Unknown	Protein of unknown function
WBGene00009880	*F49C12.11*	Unc;Dpy	Wt	3	1	Unknown	Protein of unknown function
WBGene00012704	*Y39C12A.1*	Unc;Lvl;Clr	Wt	3	1	Signalling	Protein of unknown function
WBGene00012885	*Y45F10D.4*	Gro	Wt	3	1	Metabolism	Iron-sulfur cluster assembly enzyme
WBGene00013585	*cyp-42A1*	Unc	Wt	3	1	Metabolism	Cytochrome P450
WBGene00016169	*C27F2.7*	Gro;Bmd	Wt	3	1	Unknown	Protein of unknown function
WBGene00018492	*F46E10.11*	Gro;Lvl;Prz	Wt	3	1	Unknown	Protein of unknown function
WBGene00022631	*nekl-2*	Unc;Lvl;Rol	Unc,Sck	3	1	Signalling	Serine/threonine protein kinase
