# Supplementary material for: Comparative RNAi Screens in C. elegans and C. briggsae Reveal the Impact of Developmental System Drift on Gene Function
Source: PLoS Genet. 2014 Feb 6;10(2):e1004077. doi: 10.1371/journal.pgen.1004077 (PMC3916228; doi:10.1371/journal.pgen.1004077)
Supplement: Table S3 — Quantitative analysis of brood size RNAi phenotypes for genes screened on the worm sorter in C. elegans and C. briggsae. Shown are the brood size scores as described in the methods section as well as an empirical p-value calculated from the distribution of GFP negative control RNAi replicates. (PDF) [file pgen.1004077.s009.pdf]

Gene(WBID)	Gene(Common)	Manual Phenotype	*C. elegans* Fitness	*C. briggsae* Fitness	Log2(*C. elegans* Fitness / *C. briggsae* Fitness)	Empirical P-value
WBGene00000041	*aco-2*	Different Phenotype	0.340243818	0.244541485	0.476489722	0.935483871
WBGene00000156	*apr-1*	Same Phenotype	0.496669134	1.050617284	-1.080880227	0.009677419
WBGene00000207	*asb-2*	Different Phenotype	0.244614315	0.074682814	1.711660637	1
WBGene00000252	*bli-2*	Different Phenotype	0.851588431	1.027068178	-0.270303698	0.193548387
WBGene00000254	*bli-4*	Same Phenotype	0.049707467	0.065946245	-0.407827939	0.106451613
WBGene00000254	*bli-4*	Same Phenotype	0.06082218	0.069548351	-0.193418789	0.25483871
WBGene00000255	*bli-5*	Same Phenotype	1.043742868	0.926251243	0.172290861	0.716129032
WBGene00000263	*F23H11.5*	Different Phenotype	0.268389662	0.330661323	-0.301025202	0.158064516
WBGene00000275	*bub-1*	Different Phenotype	0.799621033	0.193714051	2.045387817	1
WBGene00000286	*cal-2*	Different Phenotype	1.214638971	0.856552538	0.503913919	0.951612903
WBGene00000292	*cap-1*	Different Phenotype	0.441671678	0.646990741	-0.550770743	0.058064516
WBGene00000413	*cdt-1*	Different Phenotype	0.220588235	0.215289982	0.035074658	0.509677419
WBGene00000431	*ceh-6*	Same Phenotype	0.384900074	0.994567901	-1.369585922	0.001612903
WBGene00000453	*ceh-32*	Different Phenotype	0.821756534	1.056137899	-0.362015291	0.135483871
WBGene00000502	*chp-1*	Different Phenotype	0.247571294	0.107521043	1.203225012	1
WBGene00000615	*col-38*	Different Phenotype	0.841320072	0.242712671	1.793405337	1
WBGene00000705	*col-131*	Different Phenotype	0.224420768	1.043197225	-2.216733844	0.001612903
WBGene00000750	*col-177*	Different Phenotype	1.205157204	1.028978389	0.228008666	0.803225806
WBGene00000779	*cpn-3*	Different Phenotype	0.783140081	1.021178387	-0.382892616	0.129032258
WBGene00000817	*csn-5*	Same Phenotype	0.952076677	0.320977918	1.568603719	1
WBGene00000868	*cyb-3*	Different Phenotype	1.314523059	1.11550152	0.236846971	0.812903226
WBGene00000874	*cyk-3*	Different Phenotype	0.603841537	0.828405235	-0.456166671	0.087096774
WBGene00001065	*dpy-3*	Different Phenotype	0.586402266	1.176617537	-1.00468286	0.016129032
WBGene00001066	*dpy-4*	Different Phenotype	0.852440409	0.686796471	0.311716357	0.851612903
WBGene00001070	*dpy-8*	Different Phenotype	0.068797776	0.873990773	-3.66718421	0.001612903
WBGene00001073	*dpy-11*	Different Phenotype	0.931183571	0.896139557	0.055342181	0.541935484
WBGene00001076	*dpy-17*	Different Phenotype	1.320078962	0.81587473	0.694204667	0.990322581
WBGene00001077	*dpy-18*	Different Phenotype	0.462282032	0.409767198	0.173968789	0.719354839
WBGene00001079	*dpy-20*	Different Phenotype	0.842075578	0.224082073	1.909922486	1
WBGene00001086	*dpy-27*	Same Phenotype	0.867820464	1.165561816	-0.425557009	0.106451613
WBGene00001184	*egl-15*	Different Phenotype	1.219480519	0.998777693	0.288031207	0.838709677
WBGene00001207	*egl-43*	Different Phenotype	0.228486647	0.497638725	-1.122988902	0.009677419
WBGene00001214	*ego-1*	Different Phenotype	0.487293363	0.948395062	-0.960697578	0.016129032
WBGene00001227	*eif-3.D*	Different Phenotype	0.259708738	0.288639366	-0.15237352	0.316129032
WBGene00001229	*eif-3.F*	Different Phenotype	0.152324431	1.003541913	-2.719881613	0.001612903
WBGene00001230	*eif-3.G*	Different Phenotype	0.979393652	0.90221857	0.118411864	0.64516129
WBGene00001231	*eif-3.H*	Different Phenotype	0.105131039	0.775935982	-2.883748956	0.001612903
WBGene00001284	*emb-30*	Different Phenotype	0.734663866	0.449490662	0.708793172	0.993548387
WBGene00001330	*eps-8*	Different Phenotype	1	0.95238846	0.070377956	0.564516129
WBGene00001345	*fos-1*	Same Phenotype	0.664384796	0.868034243	-0.385732899	0.125806452

WBGene00001385	*far-1*	Different Phenotype	0.501213592	0.731175694	-0.544792573	0.061290323
WBGene00001411	*fem-1*	Different Phenotype	0.084185207	0.054976852	0.614742457	0.977419355
WBGene00001423	*fib-1*	Different Phenotype	0.509683794	0.354985192	0.521843636	0.95483871
WBGene00001561	*gei-4*	Different Phenotype	0.041244403	0.972448755	-4.559351983	0.001612903
WBGene00001570	*gei-13*	Different Phenotype	0.281553398	0.966314399	-1.779084096	0.001612903
WBGene00001627	*gly-2*	Different Phenotype	0.824712178	0.871767569	-0.080052824	0.383870968
WBGene00001832	*hcp-4*	Different Phenotype	0.05202131	0.077654086	-0.577959117	0.05483871
WBGene00001834	*hda-1*	Different Phenotype	0.299407115	0.049555775	2.594983409	1
WBGene00001840	*hel-1*	Different Phenotype	1.090524535	1.136069114	-0.059028378	0.406451613
WBGene00001862	*him-3*	Different Phenotype	0.065544197	0.51244213	-2.966849123	0.001612903
WBGene00001872	*him-14*	Different Phenotype	1.004460019	0.892584593	0.170359331	0.716129032
WBGene00001980	*hmr-1*	Same Phenotype	0.482664483	0.812826249	-0.751926324	0.029032258
WBGene00001981	*hnd-1*	Different Phenotype	0.657657658	0.83116224	-0.337793326	0.141935484
WBGene00002007	*hsp-3*	Different Phenotype	0.744854105	0.974214145	-0.387281058	0.125806452
WBGene00002047	*icp-1*	Different Phenotype	0.903846154	0.307557118	1.555222864	1
WBGene00002068	*ify-1*	Different Phenotype	0.537256763	2.236624204	-2.057639232	0.001612903
WBGene00002072	*ima-1*	Different Phenotype	0.918766188	0.914279059	0.007063188	0.493548387
WBGene00002081	*ina-1*	Different Phenotype	0.667475728	1.228533686	-0.880150135	0.019354839
WBGene00002094	*ins-11*	Different Phenotype	1.355532787	1.161754134	0.222555231	0.803225806
WBGene00002148	*gon-14*	Different Phenotype	1.067014952	0.900823776	0.244263581	0.812903226
WBGene00002152	*iars-1*	Same Phenotype	1.136428571	1.188986641	-0.065225497	0.403225806
WBGene00002229	*klp-19*	Different Phenotype	0.592146363	0.837714534	-0.500504887	0.070967742
WBGene00002231	*knl-1*	Different Phenotype	0.230043391	0.289665211	-0.332480415	0.141935484
WBGene00002245	*lag-1*	Different Phenotype	0.909744409	0.199526814	2.18887864	1
WBGene00002368	*let-99*	Different Phenotype	0.922274563	0.376935555	1.290878421	1
WBGene00002915	*let-805*	Different Phenotype	0.210735586	0.352705411	-0.743029749	0.029032258
WBGene00003001	*lin-12*	Different Phenotype	0.671930238	0.564910734	0.250288542	0.812903226
WBGene00003003	*lin-14*	Different Phenotype	0.761781076	0.458823529	0.731437072	0.996774194
WBGene00003009	*lin-23*	Different Phenotype	1.236680328	0.315600288	1.970302198	1
WBGene00003012	*lin-26*	Different Phenotype	0.932868494	0.968055978	-0.053416755	0.412903226
WBGene00003024	*lin-39*	Different Phenotype	0.994558854	1.358806405	-0.450211272	0.096774194
WBGene00003026	*lin-41*	Different Phenotype	0.103467103	0.438478747	-2.08333477	0.001612903
WBGene00003026	*lin-41*	Different Phenotype	0.125580126	0.44643301	-1.829835547	0.001612903
WBGene00003037	*lin-54*	Different Phenotype	0.808882907	0.837192155	-0.049627917	0.412903226
WBGene00003044	*lir-1*	Same Phenotype	0.190448457	0.654305928	-1.780564746	0.001612903
WBGene00003053	*lmp-1*	Different Phenotype	0.465731735	0.497298625	-0.094613251	0.370967742
WBGene00003056	*lon-2*	Different Phenotype	0.096942321	0.301326413	-1.636128609	0.001612903
WBGene00003065	*lpd-9*	Different Phenotype	0.54320697	0.150011647	1.856427475	1
WBGene00003066	*lpl-1*	Different Phenotype	0.378935223	0.274867231	0.463216326	0.925806452
WBGene00003071	*lrp-1*	Different Phenotype	0.063903282	0.554814815	-3.118044389	0.001612903
WBGene00003080	*lsm-6*	Different Phenotype	0.775609756	0.764817836	0.020214783	0.506451613

WBGene00003134	*mat-3*	Different Phenotype	0.195332136	0.460931119	-1.238621848	0.001612903
WBGene00003160	*mdf-1*	Different Phenotype	1.083318716	1.175188895	-0.117434917	0.348387097
WBGene00003209	*mel-26*	Same Phenotype	0.395390071	0.908952187	-1.200927765	0.003225806
WBGene00003210	*mel-28*	Same Phenotype	0.992513539	0.827933765	0.261571426	0.822580645
WBGene00003214	*mel-32*	Different Phenotype	1.268852459	0.62961211	1.010989129	1
WBGene00003221	*mes-3*	Different Phenotype	0.789407709	0.72549552	0.121803903	0.65483871
WBGene00003229	*mex-3*	Different Phenotype	0.548784338	0.142326379	1.947036232	1
WBGene00003367	*mix-1*	Different Phenotype	0.068361087	0.097053171	-0.505600019	0.067741935
WBGene00003392	*mog-4*	Different Phenotype	0.728861052	0.346203531	1.07402337	1
WBGene00003406	*mrg-1*	Different Phenotype	0.486836234	0.069302629	2.812454566	1
WBGene00003576	*ndc-80*	Different Phenotype	1.129642857	1.109644835	0.025768742	0.509677419
WBGene00003792	*npp-6*	Different Phenotype	0.047633873	0.086575022	-0.861962993	0.019354839
WBGene00003836	*nxt-1*	Different Phenotype	0.06902439	0.07096248	-0.039950184	0.422580645
WBGene00003901	*paa-1*	Different Phenotype	0.763505402	0.188075618	2.021325534	1
WBGene00003912	*pal-1*	Same Phenotype	0.899901865	0.910698497	-0.017205821	0.470967742
WBGene00003917	*par-2*	Different Phenotype	0.545421903	0.271712497	1.005291718	1
WBGene00003920	*par-5*	Different Phenotype	1.055854643	0.921692966	0.196053091	0.758064516
WBGene00003927	*pas-6*	Different Phenotype	1.021617727	0.985624293	0.051745743	0.541935484
WBGene00003947	*pbs-1*	Different Phenotype	1.013375295	0.725680934	0.481761292	0.938709677
WBGene00003950	*pbs-4*	Different Phenotype	0.06378276	0.156044584	-1.290719861	0.001612903
WBGene00004013	*pha-4*	Different Phenotype	1.120022916	0.72112462	0.635207747	0.987096774
WBGene00004027	*pie-1*	Different Phenotype	0.384465705	0.215089747	0.837916153	0.996774194
WBGene00004038	*plc-3*	Different Phenotype	0.283848359	0.223182145	0.346898787	0.874193548
WBGene00004125	*mdt-30*	Different Phenotype	0.849253731	1.177727784	-0.471738561	0.080645161
WBGene00004134	*pqn-47*	Different Phenotype	0.073619632	1.076233184	-3.869756349	0.001612903
WBGene00004161	*pqn-80*	Different Phenotype	0.4194556	0.458831197	-0.129445379	0.335483871
WBGene00004166	*pqn-85*	Different Phenotype	0.907360862	0.776476994	0.224733251	0.803225806
WBGene00004271	*rab-7*	Same Phenotype	0.419750177	0.812431122	-0.952714573	0.016129032
WBGene00004374	*rme-2*	Same Phenotype	0.607028754	0.281545741	1.108395528	1
WBGene00004397	*rol-6*	Different Phenotype	1.078209765	1.57138608	-0.543399805	0.061290323
WBGene00004416	*rpl-5*	Different Phenotype	0.108047016	0.081201666	0.412078006	0.9
WBGene00004425	*rpl-13*	Different Phenotype	0.054310073	0.076307516	-0.49060538	0.074193548
WBGene00004427	*rpl-15*	Different Phenotype	0.068732487	0.039328262	0.805425742	0.996774194
WBGene00004432	*rpl-20*	Different Phenotype	0.327476038	0.56466877	-0.786015495	0.025806452
WBGene00004438	*rpl-25.1*	Different Phenotype	0.213336239	0.167874094	0.345749436	0.870967742
WBGene00004452	*rpl-38*	Different Phenotype	0.124033228	0.083333333	0.573761074	0.964516129
WBGene00004453	*rpl-39*	Different Phenotype	1.272356215	1.046142093	0.282423811	0.835483871
WBGene00004456	*rpl-43*	Different Phenotype	0.063606794	0.070075758	-0.139734556	0.325806452
WBGene00004483	*rps-14*	Different Phenotype	0.490070423	0.175014732	1.485512712	1
WBGene00004489	*rps-20*	Different Phenotype	0.209276018	0.214411248	-0.034973594	0.432258065
WBGene00004700	*rsp-3*	Same Phenotype	0.522365428	0.490110275	0.091953026	0.6

WBGene00004704	*rsp-7*	Different Phenotype	0.093339176	0.14061499	-0.591195764	0.05483871
WBGene00004726	*sas-4*	Different Phenotype	0.929850746	0.367829021	1.337963851	1
WBGene00004729	*sax-3*	Different Phenotype	0.054696012	0.099675244	-0.865799573	0.019354839
WBGene00004735	*sbp-1*	Same Phenotype	0.653904473	0.628041309	0.058220437	0.54516129
WBGene00004786	*sex-1*	Same Phenotype	0.504737091	0.057518488	3.13343445	1
WBGene00004804	*skn-1*	Different Phenotype	0.94828623	0.574652778	0.722632086	0.996774194
WBGene00004949	*sox-2*	Different Phenotype	0.787830808	0.55697446	0.50027466	0.948387097
WBGene00004951	*spc-1*	Same Phenotype	0.548266972	1.183890578	-1.110585271	0.009677419
WBGene00004953	*spd-2*	Different Phenotype	0.089007092	0.074262462	0.261287154	0.819354839
WBGene00004955	*spd-5*	Different Phenotype	0.059464508	0.45861363	-2.947178486	0.001612903
WBGene00004981	*spl-1*	Different Phenotype	0.880562952	1.08973306	-0.307476725	0.151612903
WBGene00005018	*sqt-3*	Different Phenotype	0.759635309	0.913389588	-0.265923378	0.193548387
WBGene00005237	*srh-11*	Different Phenotype	0.945606695	1.03712297	-0.133274808	0.332258065
WBGene00006059	*stc-1*	Different Phenotype	0.226039783	0.663593099	-1.553722175	0.001612903
WBGene00006379	*sys-1*	Different Phenotype	0.049413049	0.045239871	0.127297284	0.658064516
WBGene00006381	*tac-1*	Different Phenotype	0.775671406	2.642857143	-1.768580919	0.001612903
WBGene00006463	*nduf-2.2*	Different Phenotype	0.492307692	0.291015625	0.758463667	0.996774194
WBGene00006481	*tag-135*	Different Phenotype	1.097175934	1.134598489	-0.048386965	0.412903226
WBGene00006565	*tfg-1*	Different Phenotype	0.228506787	1.608963093	-2.81582231	0.001612903
WBGene00006577	*tlf-1*	Different Phenotype	0.44787234	0.272889115	0.714772722	0.996774194
WBGene00006579	*tlk-1*	Different Phenotype	0.312815126	0.184210526	0.763954772	0.996774194
WBGene00006617	*tars-1*	Different Phenotype	0.152087994	0.17398374	-0.194046211	0.25483871
WBGene00006647	*tsr-1*	Same Phenotype	1.087272727	0.894672382	0.281282479	0.835483871
WBGene00006773	*unc-37*	Different Phenotype	0.992121553	0.091121495	3.444653561	1
WBGene00006780	*unc-44*	Different Phenotype	0.307507987	0.068611987	2.164091323	1
WBGene00006933	*vps-35*	Different Phenotype	1.244877049	0.465851905	1.418059963	1
WBGene00006943	*wrm-1*	Different Phenotype	1.08	0.484375	1.156835002	1
WBGene00006983	*zig-6*	Different Phenotype	0.573763347	0.894579066	-0.640753188	0.04516129
WBGene00006994	*zyg-9*	Different Phenotype	1.688659794	0.86548913	0.964291098	1
WBGene00007008	*rfp-1*	Different Phenotype	1.004477612	1.70303712	-0.761664472	0.029032258
WBGene00007012	*mdt-4*	Different Phenotype	0.802088277	0.33343949	1.266336049	1
WBGene00007016	*mdt-15*	Different Phenotype	0.252230769	0.216796875	0.218400317	0.8
WBGene00007097	*B0024.4*	Different Phenotype	0.703736187	0.528026709	0.414423793	0.9
WBGene00007110	*B0035.11*	Different Phenotype	0.715015658	0.600114827	0.252736262	0.812903226
WBGene00007171	*B0393.6*	Different Phenotype	0.863591757	1.274977896	-0.562050858	0.05483871
WBGene00007192	*B0491.5*	Different Phenotype	0.102999639	0.055871212	0.88246225	0.996774194
WBGene00007275	*C03D6.1*	Same Phenotype	0.061425061	0.886403182	-3.851063759	0.001612903
WBGene00007332	*C05C10.5*	Different Phenotype	0.867659947	1.151825753	-0.408720845	0.106451613
WBGene00007385	*atp-5*	Different Phenotype	0.078408288	0.074307011	0.077507817	0.570967742
WBGene00007585	*C14C10.2*	Different Phenotype	0.803771239	0.897786977	-0.159588213	0.306451613
WBGene00007586	*ril-2*	Different Phenotype	0.359683794	0.66041461	-0.876642876	0.019354839

WBGene00007587	*C14C10.4*	Different Phenotype	0.29071695	0.106989333	1.442148225	1
WBGene00007684	*C18E9.4*	Different Phenotype	0.426310584	0.237898465	0.841558908	0.996774194
WBGene00007744	*C26D10.3*	Different Phenotype	1.169072165	0.970108696	0.26914568	0.832258065
WBGene00007880	*C33A12.1*	Different Phenotype	0.82408898	0.356672158	1.208201516	1
WBGene00007999	*tag-297*	Different Phenotype	0.529225908	1.781632653	-1.751244309	0.001612903
WBGene00008021	*C39B10.1*	Different Phenotype	0.556546671	0.23412527	1.249222016	1
WBGene00008061	*C41G7.3*	Different Phenotype	0.760638298	0.888351984	-0.223920836	0.219354839
WBGene00008166	*saps-1*	Same Phenotype	1.624906786	1.0899729	0.576064694	0.967741935
WBGene00008262	*ril-1*	Different Phenotype	0.031620553	0.549654492	-4.119590517	0.001612903
WBGene00008363	*D1046.2*	Different Phenotype	1.079759988	0.94055464	0.199126999	0.764516129
WBGene00008413	*D2030.3*	Different Phenotype	0.162595608	0.441481481	-1.441064634	0.001612903
WBGene00008441	*E01B7.1*	Different Phenotype	0.72690167	0.587012987	0.308367798	0.848387097
WBGene00008605	*mlt-9*	Different Phenotype	0.965702545	0.309930638	1.639633501	1
WBGene00008640	*F10B5.3*	Different Phenotype	0.243298969	0.27513587	-0.177414029	0.287096774
WBGene00008684	*mig-32*	Different Phenotype	0.941453567	0.334316472	1.4936755	1
WBGene00008736	*gnrr-6*	Different Phenotype	0.904547608	0.861339359	0.070614679	0.564516129
WBGene00008990	*smgl-1*	Different Phenotype	0.045863815	0.426791277	-3.218102408	0.001612903
WBGene00009013	*F21D5.8*	Different Phenotype	0.95057979	0.28101197	1.758176147	1
WBGene00009039	*F22B3.8*	Different Phenotype	0.724107143	0.368849788	0.973169775	1
WBGene00009092	*tomm-20*	Different Phenotype	0.484044758	0.059385448	3.026959092	1
WBGene00009159	*F26E4.4*	Different Phenotype	0.050778051	0.072334079	-0.510470495	0.067741935
WBGene00009223	*F28F8.5*	Different Phenotype	0.396846011	0.235905271	0.750371649	0.996774194
WBGene00009242	*sre-6*	Different Phenotype	0.812138221	0.90318046	-0.153288988	0.312903226
WBGene00009246	*F29C12.4*	Different Phenotype	0.555457174	0.075994318	2.86971222	1
WBGene00009254	*capg-1*	Different Phenotype	0.870005627	1.110851506	-0.352569338	0.141935484
WBGene00009259	*F29G6.3*	Same Phenotype	0.885727833	0.376078498	1.235829632	1
WBGene00009264	*sac-1*	Same Phenotype	0.880496454	0.921922686	-0.066328575	0.4
WBGene00009284	*F31C3.2*	Different Phenotype	0.824631101	1.098368522	-0.413541407	0.106451613
WBGene00009347	*F32H5.1*	Different Phenotype	0.085375494	0.051530109	0.72840639	0.996774194
WBGene00009364	*F33H1.3*	Different Phenotype	1.114373357	0.919602819	0.277149877	0.832258065
WBGene00009368	*F33H2.5*	Different Phenotype	0.563348416	0.911247803	-0.693815964	0.032258065
WBGene00009371	*F33H2.8*	Different Phenotype	0.988687783	1.228471002	-0.313276893	0.148387097
WBGene00009454	*F36A2.7*	Different Phenotype	0.118430792	0.059012346	1.00495552	1
WBGene00009477	*tag-214*	Different Phenotype	0.077304965	0.12792472	-0.726662106	0.029032258
WBGene00009504	*F37B12.1*	Same Phenotype	1.075173096	1.325265643	-0.301712627	0.15483871
WBGene00009505	*F37B12.3*	Different Phenotype	0.700296736	0.340023613	1.042331426	1
WBGene00009587	*F40F11.2*	Different Phenotype	0.991071429	0.696480938	0.50890517	0.95483871
WBGene00009588	*F40F11.3*	Different Phenotype	1.08375	1.024600847	0.080970004	0.577419355
WBGene00009626	*F42A8.1*	Same Phenotype	0.836717428	1.116090713	-0.4156419	0.106451613
WBGene00009649	*F43C1.5*	Different Phenotype	1.489065606	0.998496994	0.576577329	0.967741935
WBGene00009661	*patr-1*	Different Phenotype	1.101379567	0.917615176	0.263350592	0.822580645

WBGene00009688	*F44E5.1*	Different Phenotype	0.649439827	0.178030303	1.867073033	1
WBGene00009701	*egg-3*	Different Phenotype	0.425055928	0.378319783	0.168046462	0.709677419
WBGene00009725	*F45E6.1*	Different Phenotype	0.776039197	0.815259685	-0.071130152	0.396774194
WBGene00009881	*F49C12.12*	Different Phenotype	0.078133628	0.070184984	0.154781208	0.687096774
WBGene00009915	*F52A8.1*	Different Phenotype	0.999718627	0.964174455	0.052227895	0.541935484
WBGene00009925	*F52B11.2*	Different Phenotype	0.997294098	0.949550686	0.070774	0.564516129
WBGene00010051	*F54D5.5*	Different Phenotype	0.66442953	0.291056911	1.190814923	1
WBGene00010094	*tsfm-1*	Different Phenotype	0.556775798	0.454863875	0.291661639	0.84516129
WBGene00010266	*dct-18*	Different Phenotype	0.935229068	1.553061224	-0.731723028	0.029032258
WBGene00010267	*lips-9*	Different Phenotype	1.194233807	1.918367347	-0.683793692	0.035483871
WBGene00010340	*slcf-1*	Different Phenotype	0.62434865	0.155094495	2.00920441	1
WBGene00010437	*JC8.5*	Different Phenotype	1.001691189	0.982973356	0.027213591	0.509677419
WBGene00010458	*K01C8.6*	Different Phenotype	0.954878737	0.46787257	1.029201881	1
WBGene00010638	*K07F5.14*	Different Phenotype	0.993999707	0.444535969	1.160945269	1
WBGene00010665	*K08E3.5*	Different Phenotype	0.043507363	0.060612644	-0.478359204	0.077419355
WBGene00010993	*R03E1.2*	Different Phenotype	0.951793899	0.902723735	0.076364666	0.567741935
WBGene00011109	*R07E5.1*	Different Phenotype	1.130218688	0.115230461	3.294007903	1
WBGene00011115	*R07E5.7*	Different Phenotype	0.574092247	0.736958444	-0.360300697	0.135483871
WBGene00011223	*R10H10.4*	Different Phenotype	0.796607143	0.529814272	0.588381709	0.974193548
WBGene00011350	*T01H3.4*	Different Phenotype	0.532615026	0.375945353	0.502570164	0.948387097
WBGene00011559	*umps-1*	Different Phenotype	0.981235785	1.019604411	-0.055337766	0.412903226
WBGene00011634	*T09A5.5*	Different Phenotype	0.769020619	0.323369565	1.249838377	1
WBGene00011637	*T09A5.9*	Different Phenotype	1.246391753	0.580842391	1.101538938	1
WBGene00011674	*cyp-13A8*	Different Phenotype	1.024539877	1.182254965	-0.206565062	0.241935484
WBGene00011687	*T10C6.5*	Different Phenotype	1.322543684	1.239361702	0.093718083	0.603225806
WBGene00011747	*sna-2*	Different Phenotype	0.430557588	0.766611752	-0.832289899	0.019354839
WBGene00011768	*oac-46*	Different Phenotype	0.27079566	0.11600238	1.223050218	1
WBGene00011831	*T19B10.2*	Different Phenotype	0.624440782	0.969718146	-0.635000719	0.04516129
WBGene00011885	*T21B10.3*	Different Phenotype	1.174502712	0.811719215	0.532997375	0.958064516
WBGene00012166	*nuo-6*	Different Phenotype	0.295723129	0.061266874	2.271067926	1
WBGene00012235	*W04A4.6*	Same Phenotype	0.04738933	1.05206334	-4.472515481	0.001612903
WBGene00012244	*W04D2.5*	Different Phenotype	0.61230833	0.107626174	2.508229333	1
WBGene00012256	*lpr-5*	Different Phenotype	0.064917439	0.049607073	0.388060271	0.9
WBGene00012261	*lpr-3*	Different Phenotype	0.882153359	0.98870334	-0.164508221	0.296774194
WBGene00012354	*W09C5.8*	Different Phenotype	1.017202931	0.889560835	0.193442347	0.758064516
WBGene00012433	*Y11D7A.9*	Different Phenotype	0.927264745	1.040087864	-0.1656522	0.296774194
WBGene00012469	*Y17G7B.18*	Different Phenotype	0.94036863	0.116003788	3.019054498	1
WBGene00012602	*Y38E10A.24*	Different Phenotype	0.827804107	1.887755102	-1.189310304	0.009677419
WBGene00012704	*Y39C12A.1*	Same Phenotype	0.914199192	1.055449049	-0.207276485	0.238709677
WBGene00012888	*sas-6*	Different Phenotype	0.285641806	0.345420148	-0.274145091	0.193548387
WBGene00012966	*exos-1*	Different Phenotype	0.908453374	0.969368108	-0.093632148	0.370967742

WBGene00012972	*rsa-2*	Different Phenotype	0.032410917	0.98582181	-4.926775138	0.001612903
WBGene00013021	*Y48G10A.4*	Different Phenotype	0.290862656	0.078934741	1.881605774	1
WBGene00013040	*Y49E10.21*	Different Phenotype	0.512717537	0.97718879	-0.930473069	0.016129032
WBGene00013075	*Y51A2D.7*	Different Phenotype	0.925296443	1.179269497	-0.349905903	0.141935484
WBGene00013109	*Y51H4A.15*	Different Phenotype	0.924675325	0.98579068	-0.092334451	0.374193548
WBGene00013267	*Y57A10A.27*	Different Phenotype	1.039754246	0.074810606	3.796855948	1
WBGene00013558	*Y75B8A.25*	Different Phenotype	0.05243195	0.060612644	-0.209172572	0.238709677
WBGene00013605	*Y95D11A.1*	Different Phenotype	0.904923383	0.217850288	2.054458628	1
WBGene00014087	*ZK809.5*	Different Phenotype	1.046601615	0.437693556	1.257719343	1
WBGene00014112	*ZK856.11*	Different Phenotype	0.643042147	0.714185884	-0.151386319	0.316129032
WBGene00014153	*vab-23*	Different Phenotype	0.640566741	0.965853659	-0.592455721	0.05483871
WBGene00014224	*ZK1098.7*	Different Phenotype	0.825	0.130093379	2.664846585	1
WBGene00014229	*ZK1128.3*	Same Phenotype	0.381092437	1.058786078	-1.474198248	0.001612903
WBGene00015075	*B0238.11*	Different Phenotype	0.61050754	0.512777609	0.251675848	0.812903226
WBGene00015091	*B0261.1*	Different Phenotype	0.254779066	0.069237035	1.879630894	1
WBGene00015162	*B0361.8*	Different Phenotype	1.011267606	1.136122569	-0.167953667	0.296774194
WBGene00015164	*ykt-6*	Different Phenotype	0.4	0.409546258	-0.034026413	0.432258065
WBGene00015185	*mrpl-41*	Different Phenotype	0.667542214	0.106035889	2.654306428	1
WBGene00015235	*cdc-26*	Different Phenotype	0.684449489	0.634836852	0.108558199	0.632258065
WBGene00015297	*sco-1*	Different Phenotype	0.819333333	0.155541947	2.397146804	1
WBGene00015487	*C05D11.10*	Different Phenotype	0.809230769	0.37109375	1.124767473	1
WBGene00015513	*C06A8.2*	Different Phenotype	0.606185567	0.43138587	0.490780582	0.94516129
WBGene00015515	*spdl-1*	Different Phenotype	0.611531741	0.530861186	0.204092722	0.770967742
WBGene00015591	*C08C3.4*	Different Phenotype	0.837523245	0.915574964	-0.12854878	0.335483871
WBGene00015791	*C15C7.5*	Different Phenotype	0.952449155	1.257345491	-0.400667142	0.106451613
WBGene00015809	*C16A3.4*	Different Phenotype	0.407692308	0.201171875	1.019052114	1
WBGene00015811	*C16A3.6*	Different Phenotype	0.250819672	0.163670766	0.615853844	0.977419355
WBGene00016015	*C23G10.8*	Different Phenotype	0.221311475	0.138126774	0.680085267	0.990322581
WBGene00016074	*C24H12.5*	Same Phenotype	1.016135084	0.480967917	1.079079638	1
WBGene00016115	*mdt-26*	Different Phenotype	0.888993637	1.037690104	-0.223130663	0.219354839
WBGene00016140	*rpb-2*	Different Phenotype	0.233562316	0.116268789	1.006343652	1
WBGene00016169	*C27F2.7*	Same Phenotype	0.829244357	1.082670203	-0.38472465	0.125806452
WBGene00016170	*C27F2.8*	Different Phenotype	0.903961585	1.369849733	-0.599684275	0.051612903
WBGene00016243	*tag-319*	Different Phenotype	0.888177053	0.415955111	1.094419454	1
WBGene00016250	*C30C11.4*	Different Phenotype	0.686893204	1.503963012	-1.130611371	0.009677419
WBGene00016291	*C31H1.8*	Different Phenotype	1.277672119	1.169870733	0.127168529	0.658064516
WBGene00016319	*C32D5.12*	Different Phenotype	1.019467213	0.762041697	0.419873533	0.906451613
WBGene00016356	*C33F10.8*	Different Phenotype	0.7643177	1.101608993	-0.527367897	0.061290323
WBGene00016449	*C35D10.13*	Different Phenotype	0.24577891	0.926133909	-1.913859679	0.001612903
WBGene00016493	*C37A2.7*	Different Phenotype	0.265053461	0.748182762	-1.497107349	0.001612903
WBGene00016581	*C42C1.3*	Different Phenotype	0.247420937	0.288034053	-0.219271799	0.222580645

WBGene00016638	*C44B12.5*	Different Phenotype	0.139977178	0.249486653	-0.833771012	0.019354839
WBGene00016676	*C45G9.5*	Different Phenotype	0.679097154	0.50132626	0.437868181	0.912903226
WBGene00016721	*C46G7.1*	Same Phenotype	0.106764082	1.170376901	-3.454474925	0.001612903
WBGene00016793	*arp-11*	Different Phenotype	0.58420762	0.307943417	0.92381589	1
WBGene00016955	*C55C3.5*	Different Phenotype	0.74201278	0.240536278	1.625189537	1
WBGene00016968	*epg-5*	Different Phenotype	0.864880606	0.910465967	-0.074104105	0.396774194
WBGene00016977	*C56G2.1*	Different Phenotype	1.08352459	1.126300851	-0.055860349	0.412903226
WBGene00016989	*CD4.3*	Different Phenotype	1.065393623	0.873687611	0.286197112	0.838709677
WBGene00017132	*mel-47*	Different Phenotype	0.802088277	0.36910828	1.119716925	1
WBGene00017166	*aldo-2*	Different Phenotype	1.321538462	1.050130208	0.331650191	0.858064516
WBGene00017237	*pole-2*	Different Phenotype	0.844577784	0.973503569	-0.20495597	0.24516129
WBGene00017289	*F09E5.11*	Different Phenotype	1.146179402	1.029299363	0.155170236	0.687096774
WBGene00017313	*cpsf-2*	Different Phenotype	0.434134362	0.980495519	-1.175369419	0.009677419
WBGene00017328	*F10C5.2*	Different Phenotype	1.116319569	1.096378982	0.026003511	0.509677419
WBGene00017348	*F10E7.6*	Different Phenotype	0.851776354	0.229568187	1.891551947	1
WBGene00017356	*F10E9.4*	Different Phenotype	0.505970149	1.010123735	-0.997407848	0.016129032
WBGene00017358	*F10E9.7*	Same Phenotype	0.441791045	1.087739033	-1.29989639	0.001612903
WBGene00017369	*F10G7.5*	Different Phenotype	1.013333333	0.881812615	0.200564802	0.764516129
WBGene00017605	*F19F10.9*	Different Phenotype	0.493422207	0.396942541	0.31389246	0.851612903
WBGene00017641	*csr-1*	Different Phenotype	0.494202098	0.480685528	0.040007768	0.529032258
WBGene00017683	*F21H12.1*	Different Phenotype	1.06557377	1.005751258	0.083356933	0.587096774
WBGene00017716	*F22F4.1*	Different Phenotype	0.061153579	0.055651672	0.136011888	0.667741935
WBGene00017853	*F27C1.3*	Same Phenotype	0.511125039	1.06000543	-1.052323482	0.009677419
WBGene00017916	*F29A7.6*	Same Phenotype	0.867818182	0.765462339	0.181061414	0.729032258
WBGene00017925	*F29B9.11*	Different Phenotype	1.321793863	0.51459144	1.36099784	1
WBGene00017926	*F29C4.2*	Different Phenotype	0.469379992	0.059753593	2.973658967	1
WBGene00017982	*F32D1.2*	Same Phenotype	0.488379759	0.634132338	-0.37678054	0.132258065
WBGene00017997	*F33D4.5*	Different Phenotype	0.337934843	0.078346028	2.108813062	1
WBGene00018144	*F37C4.4*	Same Phenotype	0.102281668	0.5	-2.289380502	0.001612903
WBGene00018151	*F37C12.3*	Different Phenotype	0.237957746	0.558043606	-1.22967244	0.001612903
WBGene00018213	*F39H12.2*	Different Phenotype	0.705205124	1.002585945	-0.507611052	0.067741935
WBGene00018350	*F42C5.10*	Different Phenotype	1.221888154	0.874710648	0.482234472	0.938709677
WBGene00018492	*F46E10.11*	Same Phenotype	0.524410016	0.958972702	-0.870794507	0.019354839
WBGene00018609	*F48E8.2*	Different Phenotype	1.226890756	1.21376636	0.015516055	0.503225806
WBGene00018703	*sec-3*	Different Phenotype	0.645238614	1.157631776	-0.843271745	0.019354839
WBGene00018772	*F53G12.4*	Different Phenotype	1.053863508	0.195464363	2.430710524	1
WBGene00018794	*F54C4.3*	Different Phenotype	1.079712747	0.095289954	3.502179609	1
WBGene00018846	*eef-1B.1*	Different Phenotype	0.412676056	0.935769004	-1.181142708	0.009677419
WBGene00018957	*F56C11.5*	Different Phenotype	0.06631928	0.384141331	-2.534136948	0.001612903
WBGene00018961	*F56D1.3*	Same Phenotype	0.924537257	1.207006369	-0.384629926	0.125806452
WBGene00019001	*F57B10.3*	Different Phenotype	0.826587796	0.671541386	0.299691749	0.84516129

WBGene00019076	*F59A3.3*	Different Phenotype	0.613636364	0.084235156	2.86488959	1
WBGene00019126	*F59E12.11*	Same Phenotype	0.492827869	1.724658519	-1.807154992	0.001612903
WBGene00019353	*K03B4.1*	Different Phenotype	0.241274659	0.839697065	-1.79919255	0.001612903
WBGene00019380	*K04C2.2*	Different Phenotype	0.936065574	0.189214759	2.306584975	1
WBGene00019400	*K04G7.1*	Same Phenotype	0.809859155	0.485562758	0.738013254	0.996774194
WBGene00019432	*knl-2*	Different Phenotype	0.067614505	0.076752563	-0.18288216	0.280645161
WBGene00019455	*K06H7.1*	Same Phenotype	0.243283582	1.262654668	-2.375749252	0.001612903
WBGene00019680	*K12H4.5*	Different Phenotype	0.443556719	0.407569141	0.122073775	0.65483871
WBGene00019759	*M03F4.6*	Different Phenotype	0.941626129	0.580449827	0.697982986	0.990322581
WBGene00019823	*fnta-1*	Different Phenotype	0.813617345	0.571663244	0.509184902	0.95483871
WBGene00019836	*R02F2.7*	Different Phenotype	0.456182473	0.37809016	0.270880715	0.832258065
WBGene00020105	*R148.7*	Different Phenotype	1.080516899	1.054108216	0.035698651	0.512903226
WBGene00020279	*glb-25*	Different Phenotype	0.90555464	0.924644754	-0.030097498	0.44516129
WBGene00020392	*knl-3*	Different Phenotype	1.04552352	0.730514358	0.517240984	0.95483871
WBGene00020558	*T19B4.5*	Different Phenotype	0.876400996	0.581456566	0.591919695	0.974193548
WBGene00020601	*T20B12.3*	Different Phenotype	0.309754098	0.166035951	0.899627717	1
WBGene00020705	*T22H9.1*	Same Phenotype	1.107359636	0.903124014	0.294127828	0.84516129
WBGene00020827	*T26A8.4*	Different Phenotype	0.748205411	0.269314472	1.474142642	1
WBGene00020915	*nol-5*	Different Phenotype	0.069150616	0.077736496	-0.168849984	0.296774194
WBGene00021000	*W03F9.2*	Different Phenotype	1.009863429	0.07573367	3.73708155	1
WBGene00021021	*W04B5.4*	Different Phenotype	0.564612326	0.189378758	1.575986111	1
WBGene00021060	*dct-11*	Different Phenotype	0.756080612	1.033448674	-0.450854774	0.096774194
WBGene00021061	*W06E11.1*	Different Phenotype	0.158348294	0.066975225	1.241401909	1
WBGene00021095	*mlt-8*	Different Phenotype	1.290909091	0.421922841	1.613336309	1
WBGene00021156	*Y4C6B.2*	Different Phenotype	0.743235117	0.697337963	0.091960646	0.6
WBGene00021270	*Y23H5A.3*	Different Phenotype	0.597410799	0.324949986	0.878505632	0.996774194
WBGene00021292	*Y25C1A.5*	Different Phenotype	0.219393939	0.153704838	0.5133611	0.95483871
WBGene00021465	*Y39G10AR.7*	Same Phenotype	0.057035647	1.005981512	-4.140596095	0.001612903
WBGene00021514	*Y41D4B.11*	Different Phenotype	0.079286798	0.060902493	0.380579384	0.9
WBGene00021626	*Y47D7A.14*	Same Phenotype	1.099772382	1.013568949	0.11776073	0.641935484
WBGene00021952	*vha-19*	Different Phenotype	0.526357199	0.964007782	-0.873002612	0.019354839
WBGene00022046	*Y66H1A.4*	Different Phenotype	2.00393391	1.438715953	0.478053141	0.935483871
WBGene00022117	*Y71F9AL.12*	Same Phenotype	0.064165103	0.970092442	-3.918261438	0.001612903
WBGene00022169	*Y71H2AM.4*	Different Phenotype	0.620007607	0.959137577	-0.62945185	0.04516129
WBGene00022182	*Y71H2AM.17*	Different Phenotype	0.533853176	0.531211499	0.00715664	0.493548387
WBGene00022201	*Y71H10B.1*	Same Phenotype	0.963172805	1.191606295	-0.307041088	0.151612903
WBGene00022310	*Y77E11A.7*	Different Phenotype	1.439811172	1.054474708	0.449355126	0.919354839
WBGene00022458	*Y110A7A.8*	Different Phenotype	0.057269614	0.081602808	-0.510848916	0.067741935
WBGene00022591	*cuti-1*	Different Phenotype	0.620571481	0.075921308	3.031020649	1
WBGene00022598	*ztf-8*	Different Phenotype	0.905162065	1.234125061	-0.44724057	0.096774194
WBGene00022631	*nekl-2*	Same Phenotype	0.195433705	0.857024106	-2.132656483	0.001612903

WBGene00022631	*nekl-2*	Same Phenotype	0.276324117	0.774175672	-1.486299487	0.001612903
WBGene00022742	*ZK430.7*	Different Phenotype	0.081632653	1.114649682	-3.771300207	0.001612903
WBGene00022793	*ZK686.3*	Different Phenotype	0.644672498	0.335516739	0.942181693	1
WBGene00022864	*ZK1236.5*	Different Phenotype	1.008495146	0.392998679	1.359607771	1
WBGene00044318	*tag-267*	Different Phenotype	1.004739336	0.810204082	0.310464008	0.848387097
WBGene00044321	*tag-264*	Different Phenotype	0.061861521	0.056381958	0.133808732	0.664516129
